# Enantioselective Michael/Hemiketalization Cascade Reactions between Hydroxymaleimides and 2-Hydroxynitrostyrenes for the Construction of Chiral Chroman-Fused Pyrrolidinediones

**DOI:** 10.3390/molecules27165081

**Published:** 2022-08-10

**Authors:** Dong-Hua Xie, Cheng Niu, Da-Ming Du

**Affiliations:** School of Chemistry and Chemical Engineering, Beijing Institute of Technology, No. 5 Zhongguancun South Street, Beijing 100081, China

**Keywords:** organocatalysis, asymmetric catalysis, cascade reaction, hydroxymaleimide, chroman, pyrrolidinedione

## Abstract

In this paper, the organocatalytic asymmetric Michael addition/hemiketalization cascade reactions between hydroxymaleimides and 2-hydroxynitrostyrenes were developed, which provided a new protocol for building a chiral ring-fused chroman skeleton. This squaramide-catalyzed cascade reaction provided chiral chroman-fused pyrrolidinediones with three contiguous stereocenters in good to high yields (up to 88%), with excellent diastereoselectivities (up to >20:1 dr) and enantioselectivities (up to 96% ee) at −16 °C. Moreover, a scale-up synthesis was also carried out, and a possible reaction mechanism was proposed.

## 1. Introduction

**Figure 1:**
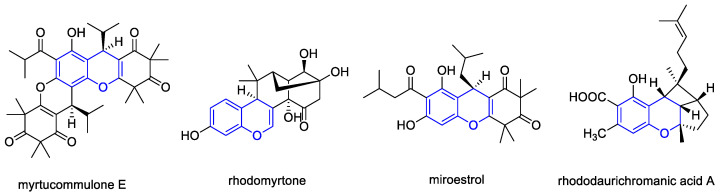
Representative examples of natural products and biologically active compounds with a ring-fused chroman skeleton.

The development of pharmaceutical science is inseparable from the discovery of lead compounds, primarily derived from natural products and analogues with biological activity. Ring-fused chroman skeletons are widely present in many natural products and analogues with bioactivity [1,2,3,4,5,6,7,8,9,10,11] (Figure 1). For example, myrtucommulone E, isolated from the Mediterranean folk herb Myrtucommulone, has α-glucosidase inhibitory activity [1]; rhodomyrtone, isolated from the leaves of myrtle, a small Indonesian shrub, has antibacterial activity [2]; miroestrol, isolated from the root of kudzu, a Thai herb, has estrogenic activity [3]; rhododaurichromanic acid A, isolated from the shoots and leaves of azalea from northern China and eastern Siberia, has anti-HIV activity [4] and so on. In addition to specific actions in the field of biomedicine, the ring-fused chroman skeleton also shows particular potential in the area of modern pesticide development. For example, greveichromenol, isolated from geronniang, the traditional herbal medicine of the Dai ethnic group, showed antitobacco mosaic virus activity, providing a lead compound for the development of antitobacco mosaic virus pesticide [5]. Therefore, the construction of the chroman skeleton has received great attention from organic and medicinal chemists. In recent years, a large number of synthetic strategies of chroman derivatives have been consistently reported [12,13,14,15,16,17,18,19,20,21,22,23,24,25,26].

**Scheme 1:**
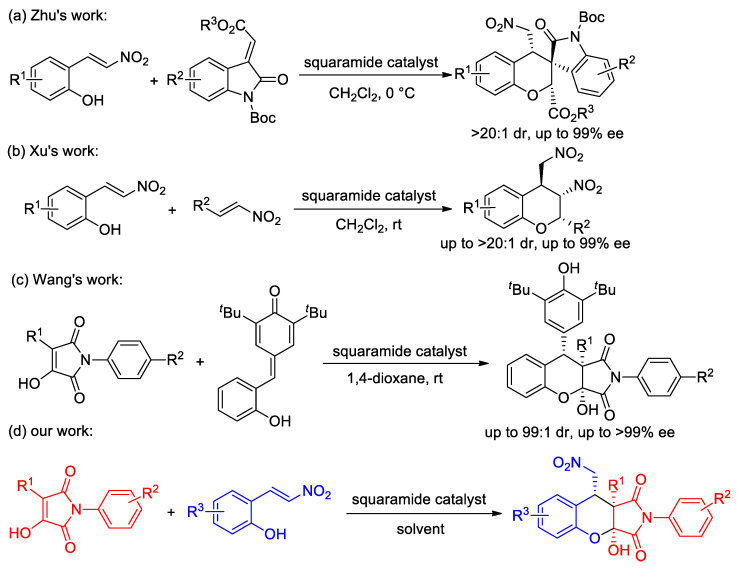
Previous reports and our work. ((**a**) oxa-Michael/Michael cascade reaction [27]; (**b**) oxa-Michael/nitro-Michael reaction [28]; (**c**) [4+2] cyclization reaction, [31]; (**d**) our research plan.).

2-Hydroxynitrostyrene plays an essential role in these reactions in the construction of chiral chroman derivatives. For example, in 2013, Zhu’s group reported a new asymmetric oxa-Michael/Michael cascade reaction for the construction of enantiomerically enriched indolinone spiro-fused chromans; this protocol offered excellent stereo control under mild conditions [27] (Scheme 1a). In 2018, Xu’s group developed an asymmetric catalytic method for the synthesis of polysubstituted chromans through an oxa-Michael-nitro-Michael reaction, and the squaramide-catalyzed domino reaction of 2-hydroxynitrostyrenes with *trans*-β-nitroolefins produced chiral chromans with excellent enantioselectivities, diastereoselectivities and yields [28] (Scheme 1b). At the same time, because of the importance of the 2,5-pyrrolidinedione framework in biomedicine [29,30], Wang’s group synthesized chiral chroman-fused pyrrolidinediones for the first time in excellent yields with excellent stereoselectivities using organocatalytic enantioselective [4+2] cyclization reaction [31] (Scheme 1c). Inspired by their work and continuing with our project with organocatalyzed domino or cascade reactions for the synthesis of bioactive heterocycles, we intend to synthesize chiral chroman-fused pyrrolidinediones using 2-hydroxynitrostyrenes as substrates to consolidate and develop this research result (Scheme 1d).

## 2. Results and Discussion

### 2.1. Optimization of Reaction Conditions

**Figure 2:**
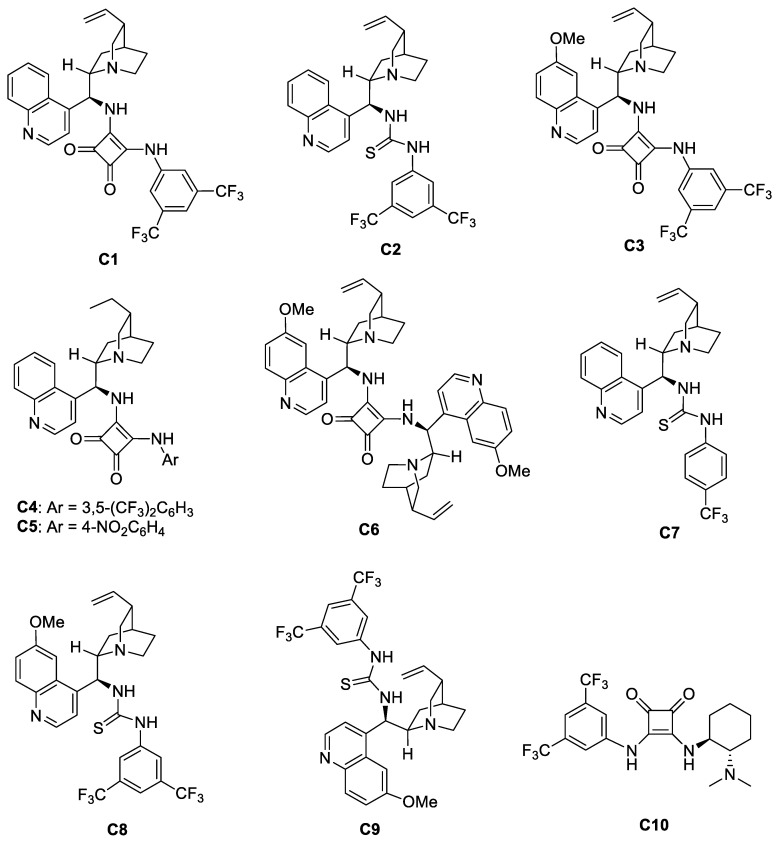
Organocatalysts selected.

Initially, we started our study with hydroxymaleimide **1a** (3-ethyl-4-hydroxy-1-phenyl-1H-pyrrole-2,5-dione) and 2-hydroxynitrostyrene **2a** as model substrates. We first tested the feasibility of the model reaction in the presence of 10 mol% cinchona-derived squaramide bifunctional catalyst **C1** (Figure 2) in dichloromethane (DCM) at room temperature. Under these conditions, the desired product **3aa** was obtained in high yield (82%) with excellent diastereoselectivity (>20:1 dr), although with moderate enantioselectivity (53% ee). Encouraged by this important result and inspired by our previous work [32,33], we tried to reduce the reaction temperature to −16 °C to improve the enantioselectivity. Luckily, the results improved to 80% yield, >20:1 dr, and 87% ee. Furthermore, we tried to screen several catalysts, reaction solvent, and catalyst loading to further improve the outcome and enantioselectivity. The results are outlined in Table 1.

Temperature is a vital factor to affect the stereoselectivity in asymmetric organic reaction, so we evaluated reaction temperature at first. Unexpectedly, a lower temperature can lead to higher yield and enantioselectivity. To avoid the contingency of the case, we took another catalyst **C2** to confirm this. We can easily find that a lower temperature is better for reaction from entries 1–4. Given the temperature conditions, we evaluated several catalysts (Table 1, entries 2, 4–12) next; the cinchona-derived bifunctional thiourea catalyst **C9** had the best yield, while the enantioselectivity was ordinary and the diastereoselectivity was not good enough (entry 11, 90% yield, −69% ee, 16:1 dr). The cinchona-derived squaramide catalyst **C6** has almost the best yield, as well as diastereoselectivity, but enantioselectivity is so low that we do not consider it (entry 8, 89% yield, 59% ee, >20:1 dr). In terms of enantioselectivity, cinchona-derived squaramide catalysts are better, compared to the diaminocyclohexane-derived squaramide catalyst **C10** (entry 12, 81% yield, 59% ee, 9:1 dr). When taking into account entry 2 (80% yield, 87% ee, >20:1 dr) and entry 4 (83% yield, 73% ee, >20:1 dr), we easily find that the cinchona-derived squaramide catalyst **C1** is superior to the cinchona-derived thiourea catalyst **C2**. Taking into account entry 2 (80% yield, 87% ee, >20:1 dr) and entry 5 (88% yield, 96% ee, >20:1 dr), we can easily find that the quinine-derived squaramide catalyst **C3** is superior to the cinchonidine-derived squaramide catalyst **C1**. Eventually, we chose the quinine-derived squaramide catalyst **C3** (entry 5, 88% yield, 96% ee, >20:1 dr) as the best catalyst in this reaction.

After a preliminary screening of the catalysts, a survey of the solvent effect using **C3** as the organocatalyst concluded that dichloromethane (DCM) was still the best solvent among 1,2-dichloroethane (DCE), chloroform, toluene, acetonitrile, tetrahydrofuran (THF). Some solvents, such as 1,4-dioxane, will freeze at −16 °C; we do not consider these (Table 1, entries 13–17). It seems unexpected that when the reaction was carried out in THF, only trace products was detected by TLC. In general, THF will have an effect on stereoselectivity owing to compete hydrogen bond formation with catalyst, but the great influence on product yield may have other reason. We finally discovered that hydroxymaleimide **1a**, one of the substrates, cannot dissolve well in THF, which could shed light on this phenomenon.

Afterwards, we further investigated the reaction with 5 and 2.5 mol% catalyst loading, respectively (Table 1, entries 18 and 19), and no improvements were obtained. Therefore, the optimal reaction conditions for this Michael/hemiketalization cascade reaction were to use a catalyst loading of 10 mol% of squaramide **C3** in DCM at −16 °C for 24 h.

### 2.2. Substrate Scope

**Scheme 2:**
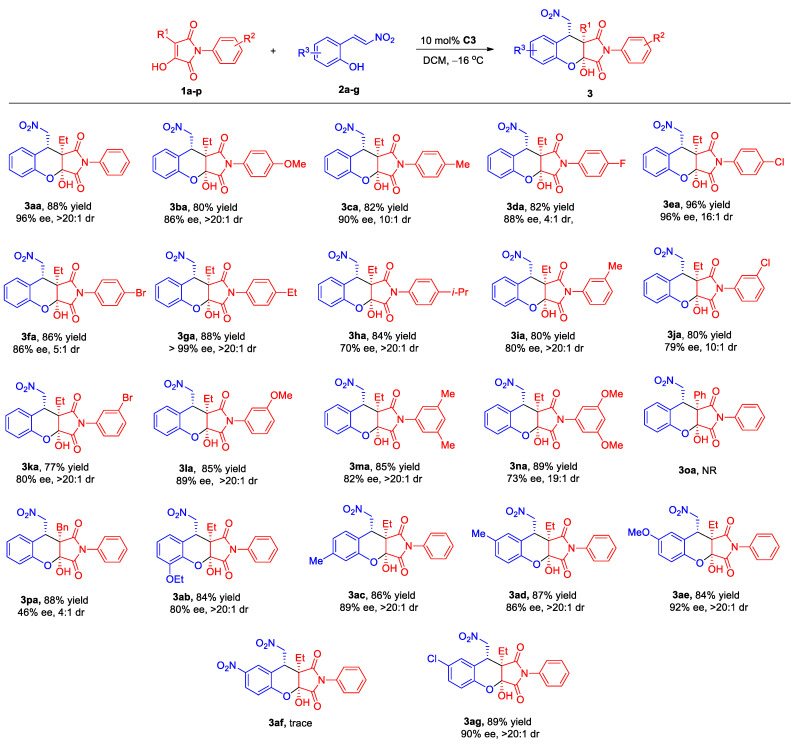
Substrate scope for chroman-fused pyrrolidinediones **3**. The reactions were carried out with **1** (0.10 mmol), **2** (0.12 mmol), and catalyst **C3** (10 mol%) in DCM (1.0 mL) at −16 °C for 24 h. The yields were isolated after column chromatography. The dr values were determined by ^1^H NMR and the ee values were determined by HPLC analysis.

With the optimized conditions in hand, we then began to investigate the substrate scope and limitation of this reaction, and the results are summarized in Scheme 2.

Firstly, we examined the tolerance of various hydroxymaleimides **1** under the optimized conditions. Various hydroxymaleimides with electron withdrawing and electron donating substituents at the 4-position on the benzene ring participated in the reaction easily, and the corresponding products **3ba**–**3ha** could be generated in high yields (79–88%) with excellent stereoselectivities (up to >20:1 dr and up to >99% ee), except products **3da** and **3fa**, whose diastereoselectivity was 4:1 and 5:1, respectively. There is no clear rule of the influence of substituents on stereoselectivity. The effect of substituents on stereoselectivity cannot be explained by the electronic effect, because the enantioselectivity and diastereoselectivity are affected by many factors. Afterwards, the cascade process gave the desired products **3ia**–**3na** with high stereoselectivity and good yield, even with the substituents at the 3-position or 3,5-position on the benzene ring. Unfortunately, substrate **1o** substituted with phenyl did not work with **2a**; no other addition was observed. The starting materials were recovered unaltered, probably because of the steric hindrance and the phenyl delocalization of the negative charge, so that the intramolecular Michael addition step cannot occur. Furthermore, we tested some substituents at different positions on 2-hydroxynitrostyrenes; most of them showed excellent results, including **3ab**–**3ae** and **3ag** (up to >20:1 dr, 92% ee). However, when the substituent is a nitro group, such as **2f**, the reaction cannot work well, probably due to the strong electron-withdrawing effect of the nitro group, which lowers the nucleophilic reactivity of the corresponding phenoxy anion. The nitro group in **2f** can block the catalyst by hydrogen bonding, and this may also hinder the reaction from proceeding.

We also tried to lower the temperature to increase the stereo control of the reaction. One can take **3ca** as an example. When the reaction was carried out at −30 °C, the trace product was detected even after 72 h; when the reaction was carried out at −20 °C, the yield decreased to 78% and the diastereoselectivity remained at 10:1 dr.

To expand the synthetic application and the substrate scope, we also tried other types of Michael accepter, such as (*E*)-3-(2-hydroxyphenyl)-1-phenylprop-2-en-1-one, (*E*)-methyl 4-(2-hydroxyphenyl)-2-oxobut-3-enoate, and 2-benzylidenemalononitrile, but the corresponding reaction results were not satisfactory, no new products were observed when these Michael acceptors reacted with **1a**, and the starting materials were recovered unchanged.

### 2.3. Scaled-Up Synthesis

**Scheme 3:**
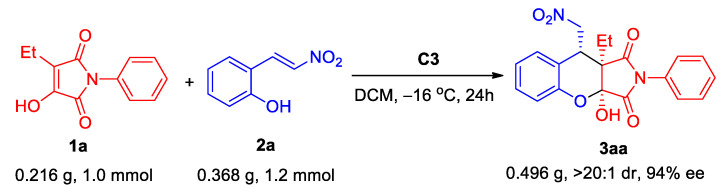
Scaled-up synthesis of **3aa**.

To prove the synthetic value of this cascade reaction, a scaled reaction of **1a** and **2a** with amplification ten times was carried out under standard conditions. As shown in Scheme 3, the desired product **3aa** was obtained in slightly reduced yield (85%), with maintained diastereoselectivity and slightly lower enantioselectivity (>20:1 dr, 94% ee). This result shows that this asymmetric catalytic strategy has broad prospects for mass production.

### 2.4. X-ray Diffraction Analysis

**Figure 3:**
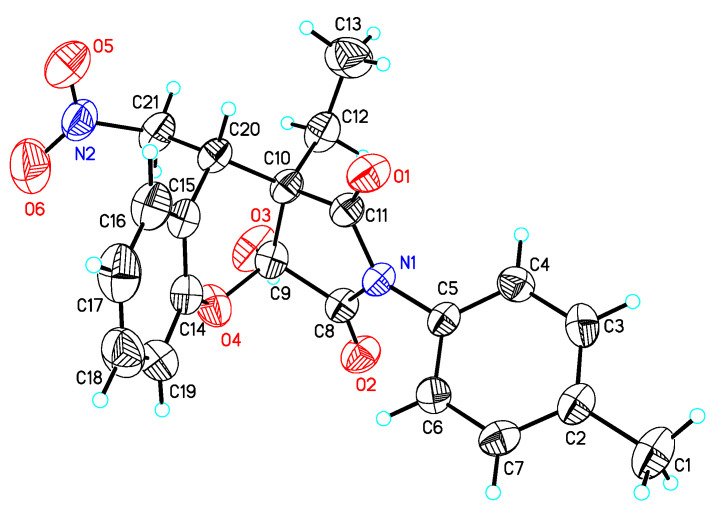
X-ray crystal structure of **3ca**.

A single crystal of **3ca** was obtained from the slow evaporation from the mixed solvents of methanol and dichloromethane. The absolute configuration of product **3ca** was unambiguously determined by single-crystal X-ray diffraction analysis as (3a*S*,9*S*,9a*R*) (Figure 3) [34] (See Supplementary Materials). The absolute configurations of the other products were assigned by analogy.

### 2.5. Controlled Reactions and Plausible Mechanism

**Scheme 4:**
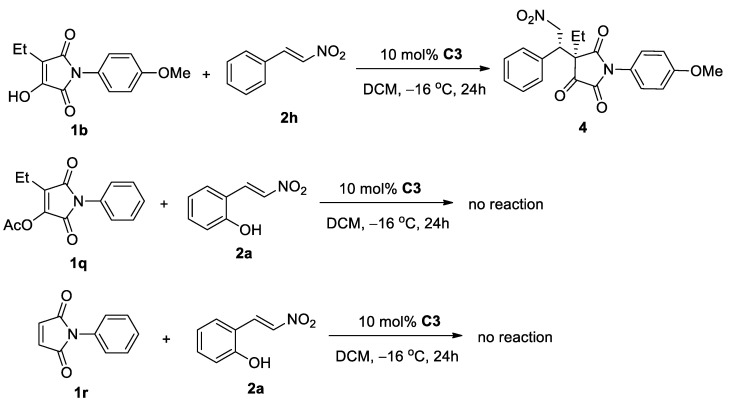
Controlled reactions to explore the mechanism.

For a better understanding of the mechanism of this cascade reaction, we designed three controlled reactions (Scheme 4). When hydroxymaleimide **1b a**nd β-nitrostyrene **2h** were used under the optimized conditions, the corresponding Michael addition product **4** was obtained with a yield of 20%. This result indicated that **1a** is a suitable Michael donor to trigger the first Michael addition step of the Michael/hemiketalization cascade reaction. When the OH group in hydroxymaleimide **1a** was protected with the acetyl group, as in substrate **1q**, no reaction was observed when **1q r**eacted with 2-hydroxynitrostyrene **2a**. Meanwhile, *N*-phenylmaleimide **1r** also could not react with **2a**. The last two control experiments indicated that the OH group in hydroxymaleimides is essential, and the hemiketalization reaction was the second step of the cascade reaction, instead of the oxa-Michael reaction as the first step.

**Scheme 5:**
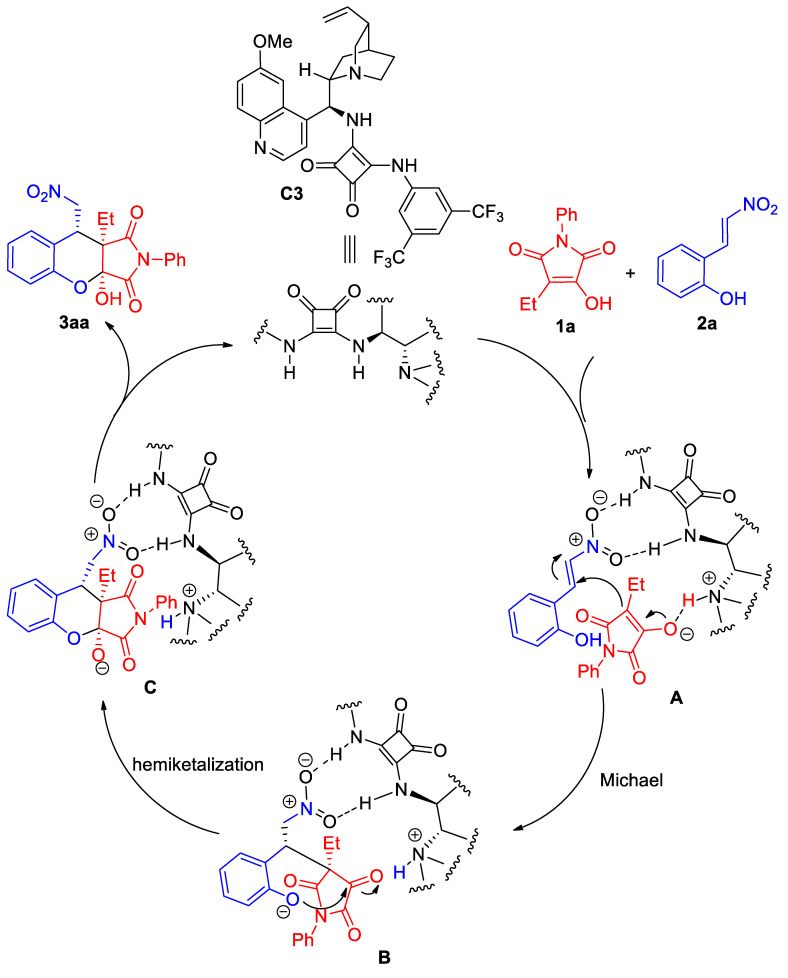
Proposed reaction mechanism.

Based on these experimental results and previous work [33], we proposed a plausible mechanism based on the absolute configuration of **3aa** (Scheme 5). In the first step of Michael addition, the squaramide catalyst **C3** initially promotes the formation of transition state **A**, and catalyst **C3** works in a double activation model. 2-Hydroxynitrostyrene **2a** is oriented and activated by the squaramide moiety through double hydrogen bonding and the OH group in **1a** is deprotoned by the tertiary amine unit to form enolate, which is oriented by another hydrogen bond. 2-Hydroxynitrostyrene **2a** is attacked by the enolate of **1a** from the *Si*-face. In the second step of the hemiketalization reaction, the OH group in 2-hydroxynitrostyrene **2a** is deprotoned by the tertiary amine unit in squaramide, and the newly formed carbonyl group in **1a** is attacked by the deprotonated phenolic hydroxyl of **2a** from the *Si*-face via transition state **B**, leading to the formation of (3a*S*,9*S*,9a*R*)-configured **3aa** and regenerates the bifunctional catalyst **C3** after a protonation process.

## 3. Conclusions

In conclusion, we have successfully developed novel Michael addition/hemiketalization cascade reactions between hydroxymaleimides and 2-hydroxynitrostyrenes to synthesize chiral ring-fused chromans. Under mild conditions, a range of structurally diverse chiral chroman-fused pyrrolidinediones, containing hemiketals, were obtained in good to high yields with excellent stereoselectivities. Additionally, the potential utility of this methodology has been demonstrated by scaling-up synthesis. This cascade synthetic strategy is bound to be a powerful tool for medicinal chemistry studies.

## 4. Materials and Methods

### 4.1. General Information

Commercially available compounds were used without further purification. Solvents were dried according to standard procedures. Column chromatography was performed with silica gel (200–300 mesh). The melting points were determined with an XT-4 melting point apparatus and were not corrected. ^1^H NMR spectra were measured with a Bruker Ascend 400 MHz spectrometer (Karlsurhe, Germany) and the chemical shifts were reported in δ (ppm) relative to tetramethylsilane (TMS) as the internal standard. ^13^C NMR spectra were measured at 100 MHz with a 400 MHz spectrometer, and the chemical shifts were reported in ppm relative to tetramethylsilane and referenced to the solvent peak (CDCl_3_, δC = 77.00 ppm; CD_3_OD, δC = 49.05 ppm; acetone-d6, δC = 30.83 ppm). High-resolution mass spectra were measured with an Agilent 6520 Accurate-Mass Q-TOF MS system (Beijing, China), equipped with an electrospray ionization (ESI) source. Optical rotations were measured with a Krüss P8000 polarimeter (Beijing, China) at the indicated concentration with units of g/100 mL. Enantiomeric excesses were determined by chiral HPLC analysis, using an Agilent 1200 LC instrument (Beijing, China) with a Daicel Chiralpak IA, IB, IC, or AD-H column.

### 4.2. Materials

Materials **1a**–**1p** were prepared according to the literature reported by Wang et al. [35] and **2a**–**2g** were prepared according to the literature [36]. The chiral organocatalysts were prepared following the procedures reported [37,38,39,40].

### 4.3. Procedure for the Asymmetric Synthesis of Compounds **3**

In a small dried bottle, **1** (0.10 mmol), **2** (0.12 mmol), chiral organocatalyst **C3** (6.0 mg, 0.01 mmol, 10 mol%) and DCM (1.0 mL) were added. The mixture was stirred at −16 °C for 24 h. After completion of the reaction, the residue was purified by flash column chromatography on silica gel to obtain the pure products **3** as solids. Racemates were prepared following a similar procedure with Et_3_N (20 mol%).

(3a*S*,9*S*,9a*R*)-9a-Ethyl-3a-hydroxy-9-(nitromethyl)-2-phenyl-9,9a-dihydrochromeno [2,3-*c*]pyrrole-1,3(2*H*,3a*H*)-dione (**3aa**). From **1a** (21.6 mg, 0.10 mmol) and **2a** (19.8 mg, 0.12 mmol), purified by silica gel (200–300 mesh) column chromatography using ethyl acetate/petroleum ether (1:8) as eluent to obtain 33.6 mg (88% yield) compound **3aa** as a white solid, m.p. 215–218 °C. HPLC (Daicel Chiralpak AD-H, n-hexane/2-propanol = 95:5, flow rate 1.0 mL/min, detection at 254 nm): *t*_R_ = 32.9 min (major), *t*_R_ = 35.3 min (minor); 96% ee. [α]_D_^25^ = +10.8 (*c* = 1.68, CH_2_Cl_2_). ^1^H NMR (400 MHz, CDCl_3_): 7.38–7.36 (m, 3H, ArH), 7.29 (dd, *J*_1_ = 1.2 Hz, *J*_2_ = 7.8 Hz, 1H, ArH), 7.18 (m, *J*_1_ = 1.2 Hz, *J*_2_ = 7.4 Hz, 1H, ArH), 7.06 (td, *J*_1_ = 7.2 Hz, *J*_2_ = 1.2 Hz, 1H, ArH), 6.99 (d, *J* = 8.0 Hz, 1H, ArH), 6.90–6.87 (m, 2H, ArH), 4.85–4.71 (m, 3H, CH_2_ + OH), 4.24 (dd, *J*_1_ = 4.4 Hz, *J*_2_ = 10.8 Hz, 1H, CH), 2.20–2.01 (m, 2H, CH_2_), 1.12 (t, *J* = 7.4 Hz, 3H, CH_3_) ppm. ^13^C NMR (100 MHz, acetone-*d*_6_): 174.1, 171.9, 150.6, 130.5, 130.3, 129.6, 129.4, 129.3, 125.9, 124.5, 124.1, 118.0, 98.1, 74.4, 55.0, 39.7, 24.8, 9.4 ppm. HRMS (ESI): *m*/*z* calcd. for C_20_H_19_N_2_O_6_ [M + H]^+^ 383.1238, found 383.1233.

(3a*S*,9*S*,9a*R*)-9a-Ethyl-3a-hydroxy-2-(4-methoxyphenyl)-9-(nitromethyl)-9,9a-dihydrochromeno[2,3-*c*]pyrrole-1,3(2*H*,3a*H*)-dione (**3ba**). From **1b** (24.6 mg, 0.10 mmol) and **2a** (19.8 mg, 0.12 mmol), purified by silica gel (200–300 mesh) column chromatography using ethyl acetate/petroleum ether (1:8) as eluent to obtain 33.0 mg (80% yield) compound **3ba** as a white solid, m.p. 184–186 °C. HPLC (Daicel Chiralpak IA, n-hexane/2-propanol = 90:10, flow rate 1.0 mL/min, detection at 254 nm): *t*_R_ = 20.0 min (minor), *t*_R_ = 22.0 min (major); 86% ee. [α]_D_^25^ = +80.9 (*c* = 1.65, CH_2_Cl_2_). ^1^H NMR (400 MHz, acetone-*d*_6_): 8.12 (s, 1H, OH), 7.32 (td, *J*_1_ = 7.6 Hz, *J*_2_ = 2.0 Hz, 1H, ArH), 7.14 (dd, *J*_1_ = 1.8 Hz, *J*_2_ = 7.4 Hz, 1H, ArH), 7.08–7.04 (m, 1H, ArH), 7.00 (d, *J* = 8.0 Hz, 1H, ArH), 6.95–6.92 (m, 2H, ArH), 6.82–6.78 (m, 2H, ArH), 5.22 (dd, *J*_1_ = 4.4 Hz, *J*_2_ = 12.8 Hz, 1H, CH_2_), 4.79 (dd, *J*_1_ = 11.6 Hz, *J*_2_ = 12.8 Hz, 1H, CH), 4.16 (dd, *J*_1_ = 4.4 Hz, *J*_2_ = 11.6 Hz, 1H, CH_2_), 3.79 (s, 3H, CH_3_), 2.30–2.21 (m, 1H, CH_2_), 2.13–2.07 (m, 1H, CH_2_), 1.11 (t, *J* = 7.4 Hz, 3H, CH_3_) ppm. ^13^C NMR (100 MHz, acetone-*d*_6_): 176.4, 173.4, 161.8, 153.2, 132.1, 131.3, 129.4, 126.7, 125.6, 125.5, 119.7, 116.2, 101.0, 76.5, 56.84, 56.79, 41.7, 26.3, 10.7 ppm. HRMS (ESI): *m*/*z* calcd. for C_21_H_21_N_2_O_7_ [M + H]^+^ 413.1343, found 413.1331.

(3a*S*,9*S*,9a*R*)-9a-Ethyl-3a-hydroxy-9-(nitromethyl)-2-(p-tolyl)-9,9a-dihydrochromeno[2,3-*c*]pyrrole-1,3(2*H*,3a*H*)-dione (**3ca**). From **1c** (23.0 mg, 0.10 mmol) and **2a** (19.8 mg, 0.12 mmol), purified by silica gel (200–300 mesh) column chromatography using ethyl acetate/petroleum ether (1:8) as eluent to obtain 32.6 mg (82% yield) compound **3ca** as a white solid, m.p. 183–185 °C. HPLC (Daicel Chiralpak IA, n-hexane/2-propanol = 94:6, flow rate 1.0 mL/min, detection at 254 nm): *t*_R_ = 22.8 min (minor), *t*_R_ = 25.0 min (major); 90% ee. [α]_D_^25^ = +58.6 (*c* = 1.63, CH_2_Cl_2_). ^1^H NMR (400 MHz, acetone-*d*_6_): 8.12, 2.80 (s, 1H, OH), 7.33 (td, *J*_1_ = 8.0 Hz, *J*_2_ = 1.6 Hz, 1H, ArH), 7.21 (d, *J* = 8.4 Hz, 2H, ArH), 7.14 (dd, 1H, *J*_1_ = 1.6 Hz, *J*_2_ = 7.2 Hz, ArH), 7.06 (td, *J*_1_ = 7.6 Hz, *J*_2_ = 0.8 Hz, 1H, ArH), 7.02–6.99 (m, 1H, ArH), 6.78 (d, *J* = 8.0 Hz, 2H, ArH), 5.22 (dd, *J*_1_ = 4.4 Hz, *J*_2_ = 12.8 Hz, 1H, CH_2_), 4.79 (dd, *J*_1_ = 11.4 Hz, *J*_2_ = 12.6 Hz, 1H, CH), 4.17 (dd, *J*_1_ = 4.2 Hz, *J*_2_ = 11.4 Hz, 1H, CH_2_), 2.32 (s, 3H, CH_3_), 2.29–2.21 (m, 1H, CH_2_), 2.15–2.08 (m, 1H, CH_2_), 1.11 (t, *J* = 7.4 Hz, 3H, CH_3_) ppm. ^13^C NMR (100 MHz, acetone-*d*_6_): 176.2, 173.3, 153.2, 140.9, 132.1, 131.5, 131.4, 130.4, 128.0, 126.7, 125.7, 119.7, 101.1, 76.5, 56.8, 41.7, 26.3, 22.1, 10.7 ppm. HRMS (ESI): *m*/*z* calcd. for C_21_H_21_N_2_O_6_ [M + H]^+^ 397.1394, found 397.1385.

(3a*S*,9*S*,9a*R*)-9a-Ethyl-2-(4-fluorophenyl)-3a-hydroxy-9-(nitromethyl)-9,9a-dihydrochromeno[2,3-*c*]pyrrole-1,3(2*H*,3a*H*)-dione (**3da**). From **1d** (23.5 mg, 0.10 mmol) and **2a** (19.8 mg, 0.12 mmol), purified by silica gel (200–300 mesh) column chromatography using ethyl acetate/petroleum ether (1:8) as eluent to obtain 32.9 mg (82% yield) compound **3da** as a white solid, m.p. 205–206 °C. HPLC (Daicel Chiralpak AD-H, n-hexane/2-propanol = 90:10, flow rate 1.0 mL/min, detection at 254 nm): *t*_R_ = 17.0 min (major), *t*_R_ = 19.2 min (minor); 88% ee. [α]_D_^25^ = +84.8 (*c* = 1.65, CH_2_Cl_2_). ^1^H NMR (400 MHz, acetone-*d*_6_): 8.28, 2.93 (s, 1H, OH), 7.33 (td, *J*_1_ = 7.6 Hz, *J*_2_ = 1.7 Hz, 1H, ArH), 7.23–7.18 (m, 2H, ArH), 7.15 (dd, *J*_1_ = 1.8 Hz, *J*_2_ = 7.4 Hz, 1H, ArH), 7.07 (td, *J*_1_ = 7.6 Hz, *J*_2_ = 0.8 Hz, 1H, ArH), 7.02–6.95 (m, 3H, ArH), 5.23 (dd, *J*_1_ = 4.4 Hz, *J*_2_ = 12.8 Hz, 1H, CH_2_), 4.80 (dd, *J*_1_ = 11.6 Hz, *J*_2_ = 12.8 Hz, 1H, CH), 4.18 (dd, *J*_1_ = 4.4 Hz, *J*_2_ = 11.6 Hz, 1H, CH_2_), 2.93 (s, OH), 2.29–2.22 (m, 1H, CH_2_), 2.17–2.09 (m, 1H, CH_2_), 1.12 (t, *J* = 7.6 Hz, 3H, CH_3_) ppm. ^13^C NMR (100 MHz, methanol-*d*_4_): δ 176.3, 173.1, 163.9 (d, ^1^*J*_C-F_ = 246.3 Hz), 152.8, 131.4, 130.7, 129.6 (d, ^3^*J*_C-F_ = 9.0 Hz), 128.3 (d, ^4^*J*_C-F_ = 3.3 Hz), 126.2, 124.9, 119.1, 117.2 (d, ^2^*J*_C-F_ = 23.3 Hz), 100.5, 75.6, 55.5, 41.4, 25.8, 9.8 ppm. HRMS (ESI): *m*/*z* calcd. for C_20_H_18_FN_2_O_6_ [M + H]^+^ 401.1143, found 401.1140.

(3a*S*,9*S*,9a*R*)-2-(4-Chlorophenyl)-9a-ethyl-3a-hydroxy-9-(nitromethyl)-9,9a-dihydrochromeno[2,3-*c*]pyrrole-1,3(2*H*,3a*H*)-dione (**3ea**). From **1e** (25.0 mg, 0.10 mmol) and **2a** (19.8 mg, 0.12 mmol), purified by silica gel (200–300 mesh) column chromatography using ethyl acetate/petroleum ether (1:8) as eluent to obtain 33.4 mg (80% yield) compound **3ea** as a white solid, m.p. 196–197 °C. HPLC (Daicel Chiralpak IB, n-hexane/ethyl acetate = 90:10, flow rate 1.0 mL/min, detection at 254 nm): *t*_R_ = 9.0 min (minor), *t*_R_ = 11.2 min (major); 96% ee. [α]_D_^25^ = +31.7 (*c* = 1.67, CH_2_Cl_2_). ^1^H NMR (400 MHz, acetone-*d*_6_): 8.22 (s, 1H, OH), 7.49–7.45 (m, 2H, ArH), 7.32 (td, *J*_1_ = 7.8 Hz, *J*_2_ = 1.6 Hz, 1H, ArH), 7.15 (dd, *J*_1_ = 1.6 Hz, *J*_2_ = 7.6 Hz, 1H, ArH), 7.07 (td, *J*_1_ = 7.6 Hz, *J*_2_ = 1.2 Hz, 1H, ArH), 7.01–6.96 (m, 3H, ArH), 5.23 (dd, *J*_1_ = 4.4 Hz, *J*_2_ = 12.8 Hz, 1H, CH_2_), 4.83–4.77 (dd, *J*_1_ = 11.6 Hz, *J*_2_ = 12.8 Hz, 1H, CH), 4.19 (dd, *J*_1_ = 4.4 Hz, *J*_2_ = 11.6 Hz, 1H, CH_2_), 2.30–2.22 (m, 1H, CH_2_), 2.17–2.10 (m, 1H, CH_2_), 1.12 (t, *J* = 7.6 Hz, 3H, CH_3_) ppm. ^13^C NMR (100 MHz, acetone-*d*_6_): δ 176.0, 172.9, 153.1, 136.1, 132.2, 131.7, 131.4, 131.2, 129.8, 126.6, 125.7, 119.7, 101.1, 76.4, 56.9, 41.4, 26.2, 10.5 ppm. HRMS (ESI): *m*/*z* calcd. for C_20_H_18_ClN_2_O_6_ [M + H]^+^ 417.0848, found 417.0849.

(3a*S*,9*S*,9a*R*)-2-(4-Bromophenyl)-9a-ethyl-3a-hydroxy-9-(nitromethyl)-9,9a-dihydrochromeno[2,3-*c*]pyrrole-1,3(2*H*,3a*H*)-dione (**3fa**). From **1f** (29.4 mg, 0.10 mmol) and **2a** (19.8 mg, 0.12 mmol), purified by silica gel (200–300 mesh) column chromatography using ethyl acetate/petroleum ether (1:8) as eluent to obtain 36.4 mg (79% yield) compound **3fa** as a white solid, m.p. 209–211 °C. HPLC (Daicel Chiralpak IB, n-hexane/2-propanol = 90:10, flow rate 1.0 mL/min, detection at 254 nm): *t*_R_ = 9.3 min (minor), *t*_R_ = 12.1 min (major); 86% ee. [α]_D_^25^ = +100.7 (*c* = 1.82, CH_2_Cl_2_). ^1^H NMR (400 MHz, acetone-*d*_6_): 8.22 (s, 1H, OH), 7.63–7.60 (m, 2H, ArH), 7.32 (td, *J*_1_ = 7.6 Hz, *J*_2_ = 1.6 Hz, 1H, ArH), 7.15 (dd, *J*_1_ = 7.6 Hz, *J*_2_ = 1.6 Hz, 1H, ArH), 7.07 (dd, *J*_1_ = 0.8 Hz, *J*_2_ = 7.6 Hz, 1H, ArH), 7.00 (d, *J* = 8.0 Hz, 1H, ArH), 6.93–6.90 (m, 2H, ArH), 5.23 (dd, *J*_1_ = 4.4 Hz, *J*_2_ = 12.8 Hz, 1H, CH_2_), 4.83–4.77 (dd, *J*_1_ = 11.6 Hz, *J*_2_ = 12.8, 1H, CH), 4.19 (dd, *J*_1_ = 4.4 Hz, *J*_2_ = 11.6 Hz, 1H, CH_2_), 2.31–2.22 (m, 1H, CH_2_), 2.17–2.08 (m, 1H, CH_2_), 1.11 (t, *J* = 7.6 Hz, 3H, CH_3_) ppm. ^13^C NMR (100 MHz, acetone-*d*_6_): 175.9, 172.8, 153.1, 134.2, 132.14, 132.12, 131.3, 130.0, 126.5, 125.7, 124.1, 119.7, 101.0, 76.4, 56.9, 41.4, 26.2, 10.5 ppm. HRMS (ESI): *m*/*z* calcd. for C_20_H_18_^79^BrN_2_O_6_ [M + H]^+^ 461.0343, found 461.0341; calcd. for C_20_H_18_^81^BrN_2_O_6_ [M + H]^+^ 463.0323, found 463.0323.

(3a*S*,9*S*,9a*R*)-9a-Ethyl-2-(4-ethylphenyl)-3a-hydroxy-9-(nitromethyl)-9,9a-dihydrochromeno[2,3-*c*]pyrrole-1,3(2*H*,3a*H*)-dione (**3ga**). From **1g** (24.4 mg, 0.10 mmol) and **2a** (19.8 mg, 0.12 mmol), purified by silica gel (200–300 mesh) column chromatography using ethyl acetate/petroleum ether (1:8) as eluent to obtain 36.2 mg (88% yield) compound **3ga** as a white solid, m.p. 189–190 °C. HPLC (Daicel Chiralpak IA, n-hexane/2-propanol = 90:10, flow rate 1.0 mL/min, detection at 254 nm): *t*_R_ = 13.5 min (minor), *t*_R_ = 15.1 min (major); >99% ee. [α]_D_^25^ = +43.1 (*c* = 1.81, CH_2_Cl_2_). ^1^H NMR (400 MHz, acetone-*d*_6_): 7.33 (td, *J*_1_ = 7.6 Hz, *J*_1_ = 1.6 Hz, 1H, ArH), 7.25 (d, *J* = 8.8 Hz, 2H, ArH), 7.14 (dd, *J*_1_ = 1.6 Hz, *J*_2_ = 7.6 Hz, 1H, ArH), 7.06 (td, *J*_1_ = 7.6 Hz, *J*_2_ = 0.8 Hz, 1H, ArH), 7.00 (d, *J* = 8.0 Hz, 1H, ArH), 6.80 (d, *J* = 8.4 Hz, 1H, ArH), 5.22 (dd, *J*_1_ = 4.4 Hz, *J*_2_ = 12.8 Hz, 1H, CH_2_), 4.80 (t, *J* = 12.2 Hz, 1H, CH), 4.16 (dd, *J*_1_ = 4.4 Hz, *J*_2_ = 11.6 Hz, 1H, CH_2_), 2.91 (s, 1H, OH), 2.63 (q, *J* = 7.6 Hz, 2H, CH_2_), 2.31–2.22 (m, 1H, CH_2_), 2.15–2.08 (m, 1H, CH), 1.18 (t, *J* = 7.6 Hz, 3H, CH_3_), 1.11 (t, *J* = 7.4 Hz, 3H, CH_3_) ppm. ^13^C NMR (100 MHz, acetone-*d*_6_): 176.3, 173.3, 153.2, 147.2, 132.1, 131.3, 130.6, 130.3, 128.1, 126.7, 125.6, 119.7, 101.0, 76.4, 56.9, 41.7, 30.0, 26.3, 16.8, 10.7 ppm. HRMS (ESI): *m*/*z* calcd. for C_22_H_23_N_2_O_6_ [M + H]^+^ 411.1551, found 411.1544.

(3a*S*,9*S*,9a*R*)-9a-Ethyl-3a-hydroxy-2-(4-isopropylphenyl)-9-(nitromethyl)-9,9a-dihydrochromeno[2,3-*c*]pyrrole-1,3(2*H*,3a*H*)-dione (**3ha**). From **1h** (26.0 mg, 0.10 mmol) and **2a** (19.8 mg, 0.12 mmol), purified by silica gel (200–300 mesh) column chromatography using ethyl acetate/petroleum ether (1:8) as eluent to obtain 35.7 mg (84% yield) compound **3ha** as a white solid, m.p. 192–193 °C. HPLC (Daicel Chiralpak IA, n-hexane/2-propanol = 95:5, flow rate 1.0 mL/min, detection at 254 nm): *t*_R_ = 19.8 min (minor), *t*_R_ = 23.0 min (major); 70% ee. [α]_D_^25^ = +9.3 (*c* = 1.79, CH_2_Cl_2_). ^1^H NMR (400 MHz, acetone-*d*_6_): 8.24 (s, OH), 7.34–7.27 (m, 3H, ArH), 7.14 (d, *J* = 7.6 Hz, 1H, ArH), 7.06 (t, *J* = 7.2 Hz, 1H, ArH), 7.00 (d, *J* = 8.0 Hz, 1H, ArH), 6.82 (d, *J* = 7.2 Hz, 2H, ArH), 5.22 (dd, *J*_1_ = 3.2 Hz, *J*_2_ = 12.8 Hz, 1H, CH_2_), 4.81 (t, *J* = 12.2 Hz, 1H, CH), 4.17 (dd, *J*_1_ = 3.2 Hz, *J*_2_ = 11.6 Hz, 1H, CH_2_), 3.07 (d, *J* = 10.4 Hz, 1H, CH), 2.94–2.86 (m, 1H, CH), 2.30–2.22 (m, 1H, CH_2_), 2.15–2.08 (m, 1H, CH_2_), 1.196 (d, *J* = 6.8 Hz, 3H, CH_3_), 1.192 (d, *J* = 6.8 Hz, 3H, CH_3_), 1.11 (t, *J* = 6.8 Hz, 3H, CH_3_) ppm. ^13^C NMR (100 MHz, acetone-*d*_6_): 176.3, 173.2, 153.2, 151.7, 132.0, 131.3, 130.6, 128.9, 128.0, 126.7, 125.6, 119.6, 101.0, 76.4, 56.8, 41.7, 35.5, 26.3, 25.0, 10.7 ppm. HRMS (ESI): *m*/*z* calcd. for C_23_H_25_N_2_O_6_ [M + H]^+^ 425.1707, found 425.1699.

(3a*S*,9*S*,9a*R*)-9a-Ethyl-3a-hydroxy-9-(nitromethyl)-2-(m-tolyl)-9,9a-dihydrochromeno[2,3-*c*]pyrrole-1,3(2*H*,3a*H*)-dione (**3ia**). From **1i** (23.0 mg, 0.10 mmol) and **2a** (19.8 mg, 0.12 mmol), purified by silica gel (200–300 mesh) column chromatography using ethyl acetate/petroleum ether (1:8) as eluent to obtain 35.3 mg (89% yield) compound **3ia** as a white solid, m.p. 203–204 °C. HPLC (Daicel Chiralpak IC, n-hexane/2-propanol = 95:5, flow rate 1.0 mL/min, detection at 254 nm): *t*_R_ = 12.2 min (major), *t*_R_ = 14.3 min (minor); 80% ee. [α]_D_^25^ = +6.0 (*c* = 1.77, CH_2_Cl_2_). ^1^H NMR (400 MHz, acetone-*d*_6_): 8.14 (s, 1H, OH), 7.33 (td, *J*_1_ = 7.6 Hz, *J*_2_ = 1.6 Hz, 1H, ArH), 7.28 (t, *J* = 7.8 Hz, 1H, ArH), 7.21 (d, *J* = 7.6 Hz, 1H, ArH), 7.14 (dd, *J*_1_ = 1.6 Hz, *J*_2_ = 7.6 Hz, 1H, ArH), 7.07 (td, *J*_1_ = 7.6 Hz, *J*_2_ = 0.8 Hz, 1H, ArH), 7.00 (d, *J* = 8.0 Hz, 1H, ArH), 6.73 (s, 1H, ArH), 6.66 (d, *J* = 8.0 Hz, 1H, ArH), 5.22 (dd, *J*_1_ = 4.4 Hz, *J*_2_ = 12.8 Hz, 1H, CH_2_), 4.83–4.78 (m, 1H, CH), 4.17 (dd, *J*_1_ = 4.4 Hz, *J*_2_ = 11.6 Hz, 1H, CH_2_), 2.28 (s, 3H, CH_3_), 2.26–2.22 (m, 1H, CH_2_), 2.16–2.09 (m, 1H, CH_2_), 1.12 (t, *J* = 7.4 Hz, 3H, CH_3_), ppm. ^13^C NMR (100 MHz, acetone-*d*_6_): 176.2, 173.2, 153.2, 141.0, 133.0, 132.1, 131.5, 131.4, 130.8, 128.7, 126.7, 125.7, 125.3, 119.7, 101.1, 76.5, 56.8, 41.7, 26.3, 22.1, 10.7 ppm. HRMS (ESI): *m*/*z* calcd. for C_21_H_21_N_2_O_6_ [M + H]^+^ 397.1394, found 397.1390.

(3a*S*,9*S*,9a*R*)-2-(3-Chlorophenyl)-9a-ethyl-3a-hydroxy-9-(nitromethyl)-9,9a-dihydrochromeno[2,3-*c*]pyrrole-1,3(2*H*,3a*H*)-dione (**3ja**). From **1j** (25.1 mg, 0.10 mmol) and **2a** (19.8 mg, 0.12 mmol), purified by silica gel (200–300 mesh) column chromatography using ethyl acetate/petroleum ether (1:8) as eluent to obtain 33.4 mg (80% yield) compound **3ja** as a white solid, m.p. 185–187 °C. HPLC (Daicel Chiralpak AD-H, n-hexane/2-propanol = 95:5, flow rate 1.0 mL/min, detection at 254 nm): *t*_R_ = 30.1 min (major), *t*_R_ = 33.5 min (minor); 79% ee. [α]_D_^25^ = +51.2 (*c* = 1.67, CH_2_Cl_2_). ^1^H NMR (400 MHz, acetone-*d*_6_): 7.46–7.45 (m, 2H, ArH), 7.34 (td, *J*_1_ = 7.8 Hz, *J*_2_ = 1.6 Hz, 1H, ArH), 7.16 (dd, *J*_1_ = 1.6 Hz, *J*_2_ = 7.6 Hz, 1H, ArH), 7.08 (t, *J* = 7.0 Hz, 1H, ArH), 7.03–7.01 (m, 1H, ArH), 6.92–6.89 (m, 1H, ArH), 5.23 (dd, *J*_1_ = 4.4 Hz, *J*_2_ = 12.8 Hz, 1H, CH_2_), 4.80 (t, *J* = 12.2 Hz, 1H, CH), 4.20 (dd, *J*_1_ = 4.4 Hz, *J*_2_ = 11.6 Hz, 1H, CH_2_), 2.97 (s, 1H, OH), 2.32–2.22 (m, 1H, CH_2_), 2.18–2.11 (m, 1H, CH_2_), 1.12 (t, *J* = 7.4 Hz, 3H, CH_3_) ppm. ^13^C NMR (100 MHz, acetone-*d*_6_): 175.8, 172.7, 153.1, 135.8, 134.1, 132.5, 132.1, 131.3, 131.0, 128.1, 126.8, 126.5, 125.7, 119.7, 101.0, 76.4, 56.9, 41.3, 26.0, 10.5 ppm. HRMS (ESI): *m*/*z* calcd. for C_20_H_18_ClN_2_O_6_ [M + H]^+^ 417.0848, found 417.0844.

(3a*S*,9*S*,9a*R*)-2-(3-Bromophenyl)-9a-ethyl-3a-hydroxy-9-(nitromethyl)-9,9a-dihydrochromeno[2,3-*c*]pyrrole-1,3(2*H*,3a*H*)-dione (**3ka**). From **1k** (29.4 mg, 0.10 mmol) and **2a** (19.8 mg, 0.12 mmol), purified by silica gel (200–300 mesh) column chromatography using ethyl acetate/petroleum ether (1:9) as eluent to obtain 35.5 mg (77% yield) compound **3ka** as a white solid, m.p. 197–198 °C. HPLC (Daicel Chiralpak IC, n-hexane/2-propanol = 97:3, flow rate 1.0 mL/min, detection at 254 nm): *t*_R_ = 18.6 min (major), *t*_R_ = 23.2 min (minor); 80% ee. [α]_D_^25^ = +136.0 (*c* = 1.78, CH_2_Cl_2_). ^1^H NMR (400 MHz, acetone-*d*_6_): 8.34, 3.08 (s, 1H, OH), 7.60–7.57 (m, 1H, ArH), 7.38 (t, *J* = 8.0 Hz, 1H, ArH), 7.33 (td, *J*_1_ = 8.0 Hz, *J*_2_ = 1.6 Hz, 1H, ArH), 7.18–7.15 (m, 2H, ArH), 7.07 (t, *J* = 7.4 Hz, 1H, ArH), 7.02 (d, *J* = 8.0 Hz, 1H, ArH), 6.95 (dd, *J*_1_ = 8.0 Hz, *J*_2_ = 0.8 Hz, 1H), 5.23 (dd, *J*_1_ = 4.4 Hz, *J*_2_ = 12.8 Hz, 2H, CH_2_), 4.81 (t, *J* = 12.2 Hz, 1H, CH), 4.20 (dd, *J*_1_ = 4.4 Hz, *J*_2_ = 11.6 Hz, 1H, CH_2_), 2.32–2.23 (m, 1H, CH_2_), 2.18–2.09 (m, 1H, CH_2_), 1.12 (t, *J* = 7.4 Hz, 3H, CH_3_) ppm. ^13^C NMR (100 MHz, acetone-*d*_6_): 175.8, 172.7, 153.0, 134.2, 133.9, 132.7, 132.1, 131.3, 130.9, 127.2, 126.5, 125.7, 123.5, 119.7, 101.0, 76.3, 56.9, 41.3, 26.0, 10.5 ppm. HRMS (ESI): *m*/*z* calcd. for C_20_H_18_^79^BrN_2_O_6_ [M + H]^+^ 461.0343, found 461.0329; calcd. for C_20_H_18_^81^BrN_2_O_6_ [M + H]^+^ 463.0323, found 463.0315.

(3a*S*,9*S*,9a*R*)-9a-ethyl-3a-hydroxy-2-(3-methoxyphenyl)-9-(nitromethyl)-9,9a-dihydrochromeno[2,3-*c*]pyrrole-1,3(2*H*,3a*H*)-dione (**3la**). From **1l** (24.6 mg, 0.10 mmol) and **2a** (19.8 mg, 0.12 mmol), purified by silica gel (200–300 mesh) column chromatography using ethyl acetate/petroleum ether (1:9) as eluent to obtain 35.1 mg (85% yield) compound **3la** as a white solid, m.p. 191–192 °C. HPLC (Daicel Chiralpak AD-H, n-hexane/2-propanol = 95:5, flow rate 1.0 mL/min, detection at 254 nm): *t*_R_ = 22.3 min (minor), *t*_R_ = 23.1 min (major); 89% ee. [α]_D_^25^ = +47.4 (*c* = 1.76, CH_2_Cl_2_). ^1^H NMR (400 MHz, acetone-*d*_6_): 8.25, 2.99 (s, 1H, OH), 7.36–7.29 (m, 2H, ArH), 7.15 (dd, *J*_1_ = 1.6 Hz, *J*_2_ = 7.6 Hz, 1H, ArH), 7.07 (td, *J*_1_ = 7.6 Hz, *J*_2_ = 1.2 Hz, 1H, ArH), 7.02 (d, *J* = 8.0 Hz, 1H, ArH), 6.98–6.95 (m, 1H, ArH), 6.48–6.46 (m, 1H, ArH), 6.38 (t, *J* = 2.2 Hz, 1H, ArH), 5.22 (dd, *J*_1_ = 4.4 Hz, *J*_2_ = 12.8 Hz, 1H, CH_2_), 4.80 (dd, *J*_1_ = 11.6 Hz, *J*_2_ = 12.8 Hz, 1H, CH), 4.18 (dd, *J*_1_ = 4.4 Hz, *J*_2_ = 11.6 Hz, 1H, CH_2_), 3.73 (s, 1H, CH_3_), 2.30–2.22 (m, 1H, CH_2_), 2.15–2.08 (m, 1H, CH_2_), 1.12 (t, *J* = 7.4 Hz, 3H, CH_3_) ppm. ^13^C NMR (100 MHz, acetone-*d*_6_): 176.1, 173.0, 162.0, 153.2, 134.0, 132.1, 131.8, 131.4, 126.8, 125.7, 120.3, 119.7, 116.1, 114.3, 101.0, 76.4, 56.9, 56.8, 41.7, 26.2, 10.6 ppm. HRMS (ESI): *m*/*z* calcd. for C_21_H_21_N_2_O_7_ [M + H]^+^ 413.1343, found 413.1339.

(3a*S*,9*S*,9a*R*)-2-(3,5-Dimethylphenyl)-9a-ethyl-3a-hydroxy-9-(nitromethyl)-9,9a-dihydrochromeno[2,3-*c*]pyrrole-1,3(2*H*,3a*H*)-dione (**3ma**). From **1m** (24.6 mg, 0.10 mmol) and **2a** (19.8 mg, 0.12 mmol), purified by silica gel (200–300 mesh) column chromatography using ethyl acetate/petroleum ether (1:7) as eluent to obtain 34.9 mg (85% yield) compound **3ma** as a white solid, m.p. 176–178 °C. HPLC (Daicel Chiralpak AD-H, n-hexane/2-propanol = 95:5, flow rate 1.0 mL/min, detection at 254 nm): *t*_R_ = 17.7 min (minor), *t*_R_ = 19.8 min (major); 82% ee. [α]_D_^25^ = +16.7 (*c* = 1.75, CH_2_Cl_2_). ^1^H NMR (400 MHz, acetone-*d*_6_): 8.13 (s, 1H, OH), 7.33 (td, *J*_1_ = 7.6 Hz, *J*_2_ = 1.6 Hz, 1H, ArH), 7.14 (dd, *J*_1_ = 1.6 Hz, *J*_2_ = 7.6 Hz, 1H, ArH), 7.07 (td, *J*_1_ = 7.6 Hz, *J*_2_ = 0.8 Hz, 1H, ArH), 7.00 (d, *J* = 8.4 Hz, 2H, ArH), 6.48 (s, 2H, ArH), 5.21 (dd, *J*_1_ = 4.4 Hz, *J*_2_ = 12.8 Hz, 1H, CH_2_), 4.79 (t, *J* = 12.0 Hz, 1H, CH), 4.17 (dd, *J*_1_ = 4.2 Hz, *J*_2_ = 11.4 Hz, 1H, CH_2_), 2.28–2.24 (m, 1H, CH_2_), 2.22 (s, 6H, CH_3_), 2.15–2.07 (m, 1H, CH_2_), 1.11 (t, *J* = 7.4 Hz, 3H, CH_3_) ppm. ^13^C NMR (100 MHz, acetone-*d*_6_): 176.2, 173.3, 153.2, 140.7, 133.0, 132.3, 132.0, 131.3, 126.6, 125.8, 125.6, 119.7, 101.0, 76.5, 56.8, 41.7, 26.3, 22.0, 10.6 ppm. HRMS (ESI): *m*/*z* calcd. for C_22_H_23_N_2_O_6_ [M + H]^+^ 411.1551, found 411.1550.

(3a*S*,9*S*,9a*R*)-2-(3,5-dimethoxyphenyl)-9a-ethyl-3a-hydroxy-9-(nitromethyl)-9,9a-dihydrochromeno[2,3-*c*]pyrrole-1,3(2*H*,3a*H*)-dione (**3na**). From **1n** (27.6 mg, 0.10 mmol) and **2a** (19.8 mg, 0.12 mmol), purified by silica gel (200–300 mesh) column chromatography using ethyl acetate/petroleum ether (1:8) as eluent to obtain 39.4 mg (89% yield) compound **3na** as a white solid, m.p. 180–181 °C. HPLC (Daicel Chiralpak AD-H, n-hexane/2-propanol = 85:15, flow rate 1.0 mL/min, detection at 254 nm): *t*_R_ = 12.9 min (minor), *t*_R_ = 16.4 min (major); 73% ee. [α]_D_^25^ = +89.3 (*c* = 1.97, CH_2_Cl_2_). ^1^H NMR (400 MHz, acetone-*d*_6_): 8.23, 2.99 (s, 1H, OH), 7.34 (td, *J*_1_ = 7.6 Hz, *J*_2_ = 1.6 Hz, 1H, ArH), 7.16 (dd, *J*_1_ = 1.6 Hz, *J*_2_ = 7.2 Hz, 1H, ArH), 7.10–7.06 (m, 1H, ArH), 7.02 (d, *J* = 8.0 Hz, 2H, ArH), 6.51 (t, *J* = 2.2 Hz, 1H, ArH), 5.97 (d, *J* = 2.0 Hz, 2H, ArH), 5.21 (dd, *J*_1_ = 4.4 Hz, *J*_2_ = 12.8 Hz, 1H, CH_2_), 4.81 (t, *J* = 12.2 Hz, 1H, CH), 4.18 (dd, *J*_1_ = 4.2 Hz, *J*_2_ = 11.4 Hz, 1H, CH_2_), 3.71 (s, 6H, CH_3_), 2.29–2.20 (m, 1H, CH_2_), 2.14–2.06 (m, 1H, CH_2_), 1.11 (t, *J* = 7.4 Hz, 3H, CH_3_) ppm. ^13^C NMR (100 MHz, acetone-*d*_6_): 176.0, 173.0, 163.0, 153.2, 134.5, 132.0, 131.4, 126.9, 125.7, 119.8, 106.7, 102.2, 101.1, 101.0, 76.3, 56.9, 41.9, 26.1, 10.6 ppm. HRMS (ESI): *m*/*z* calcd. for C_22_H_23_N_2_O_8_ [M + H]^+^ 443.1449, found 443.1448

(3a*S*,9*S*,9a*R*)-9a-Benzyl-3a-hydroxy-9-(nitromethyl)-2-phenyl-9,9a-dihydrochromeno[2,3-*c*]pyrrole-1,3(2*H*,3a*H*)-dione (**3qa**). From **1q** (28.0 mg, 0.10 mmol) and **2a** (19.8 mg, 0.12 mmol), purified by silica gel (200–300 mesh) column chromatography using ethyl acetate/petroleum ether (1:10) as eluent to obtain 39.2 mg (88% yield) compound **3qa** as a white solid, m.p. 210–211 °C. HPLC (Daicel Chiralpak IB, n-hexane/2-propanol = 85:15, flow rate 1.0 mL/min, detection at 254 nm): *t*_R_ = 8.3 min (major), *t*_R_ = 9.3 min (minor); 46% ee. [α]_D_^25^ = +69.9 (*c* = 1.96, CH_2_Cl_2_). ^1^H NMR (400 MHz, acetone-*d*_6_): 7.41–7.39 (m, 1H, ArH), 7.34–7.21 (m, 7H, ArH), 7.17 (dd, *J*_1_ = 1.6 Hz, *J*_2_ = 7.6 Hz, 1H, ArH), 7.06 (td, *J*_1_ = 7.4 Hz, *J*_2_ = 0.8 Hz, 1H, ArH), 6.98 (d, *J* = 8.4 Hz, 2H, ArH), 6.19–6.16 (m, 2H, ArH), 5.43 (dd, *J*_1_ = 4.4 Hz, *J*_2_ = 12.8 Hz, 1H, CH_2_), 4.94 (t, *J* = 12.2 Hz, 1H, CH), 4.34 (dd, *J*_1_ = 4.4 Hz, *J*_2_ = 11.6 Hz, 1H, CH_2_), 3.84 (d, *J* = 13.2 Hz, 1H, CH_2_), 3.26 (d, *J* = 13.2 Hz, 1H, CH_2_), 2.93 (s, 1H, OH) ppm. ^13^C NMR (100 MHz, acetone-*d*_6_): 174.8, 172.4, 153.1, 136.9, 132.7, 132.6, 132.1, 131.4, 130.7, 130.6, 130.3, 129.4, 128.1, 126.6, 125.5, 119.5, 100.6, 76.4, 58.6, 43.3, 39.1 ppm. HRMS (ESI): *m*/*z* calcd. for C_25_H_21_N_2_O_6_ [M + H]^+^ 445.1394, found 445.1393.

(3a*S*,9*S*,9a*R*)-5-Ethoxy-9a-ethyl-3a-hydroxy-9-(nitromethyl)-2-phenyl-9,9a-dihydrochromeno[2,3-*c*]pyrrole-1,3(2*H*,3a*H*)-dione (**3ab**). From **1a** (21.6 mg, 0.10 mmol) and **2b** (25.0 mg, 0.12 mmol), purified by silica gel (200–300 mesh) column chromatography using ethyl acetate/petroleum ether (1:8) as eluent to obtain 35.9 mg (84% yield) compound **3ab** as a white solid, m.p. 186–187 °C. HPLC (Daicel Chiralpak IA, n-hexane/2-propanol = 88:12, flow rate 1.0 mL/min, detection at 254 nm): *t*_R_ = 17.1 min (major), *t*_R_ = 20.2 min (minor); 80% ee. [α]_D_^25^ = +26.6 (*c* = 1.80, CH_2_Cl_2_). ^1^H NMR (400 MHz, acetone-*d*_6_): 7.44–7.39 (m, 3H, ArH), 7.01–6.90 (m, 4H, ArH), 6.69 (dd, *J*_1_ = 2.0 Hz, *J*_2_ = 6.8 Hz, 1H, ArH), 5.21 (dd, *J*_1_ = 4.4 Hz, *J*_2_ = 12.6 Hz, 1H, CH_2_), 4.81 (t, *J* = 12.0 Hz, 1H, CH), 4.14 (dd, *J*_1_ = 4.4 Hz, *J*_2_ = 11.6 Hz, 1H, CH_2_), 4.08–4.00 (m, 2H, CH_2_), 2.92 (s, 1H, OH), 2.29–2.24 (m, 1H, CH_2_), 2.14–2.07 (m, 1H, CH_2_), 1.30 (t, *J* = 6.8 Hz, 3H, CH_3_), 1.11 (t, *J* = 7.6 Hz, 3H, CH_3_) ppm. ^13^C NMR (100 MHz, acetone-*d*_6_): 176.0, 173.0, 150.8, 142.4, 133.1, 131.0, 130.8, 128.2, 128.1, 125.6, 122.7, 116.3, 101.0, 76.3, 66.4, 56.9, 42.0, 26.2, 16.1, 10.7 ppm. HRMS (ESI): *m*/*z* calcd. for C_22_H_23_N_2_O_7_ [M + H]^+^ 427.1500, found 427.1494.

(3a*S*,9*S*,9a*R*)-9a-Ethyl-3a-hydroxy-6-methyl-9-(nitromethyl)-2-phenyl-9,9a-dihydrochromeno[2,3-*c*]pyrrole-1,3(2*H*,3a*H*)-dione (**3ac**). From **1a** (21.6 mg, 0.10 mmol) and **2c** (17.8 mg, 0.12 mmol), purified by silica gel (200–300 mesh) column chromatography using ethyl acetate/petroleum ether (1:9) as eluent to obtain 34.1 mg (86% yield) compound **3ac** as a white solid, m.p. 183–185 °C. HPLC (Daicel Chiralpak AD-H, n-hexane/2-propanol = 85:15, flow rate 1.0 mL/min, detection at 254 nm): *t*_R_ = 10.1 min (minor), *t*_R_ = 11.1 min (major); 89% ee. [α]_D_^25^ = +1.05 (*c* = 1.71, CH_2_Cl_2_). ^1^H NMR (400 MHz, acetone-d_6_): 8.21, 2.96 (s, 1H, OH), 7.45–7.39 (m, 3H, ArH), 7.00 (d, *J* = 7.6 Hz, 1H, ArH), 6.93 (dd, *J*_1_ = 1.8 Hz, *J*_2_ = 8.2 Hz, 2H, ArH), 6.87 (d, *J* = 7.6 Hz, 1H, ArH), 6.83 (s, 1H, ArH), 5.19 (dd, *J*_1_ = 4.4 Hz, *J*_2_ = 12.4 Hz, 1H, CH_2_), 4.77 (t, *J* = 12.2 Hz, 1H, CH), 4.14 (dd, *J*_1_ = 4.4 Hz, *J*_2_ = 11.6 Hz, 1H, CH_2_), 2.28 (s, 3H, CH_3_), 2.25–2.20 (m, 1H, CH_2_), 2.14–2.07 (m, 1H, CH_2_), 1.11 (t, *J* = 7.4 Hz, 3H, CH_3_) ppm. ^13^C NMR (100 MHz, acetone-*d*_6_): 176.2, 173.2, 153.0, 142.4, 133.1, 131.0, 130.8, 128.2, 126.3, 123.4, 120.0, 100.9, 76.6, 56.8, 41.2, 28.5, 26.3, 22.1, 10.6 ppm. HRMS (ESI): *m*/*z* calcd. for C_21_H_21_N_2_O_6_ [M + H]^+^ 397.1394 found 397.1388.

(3a*S*,9*S*,9a*R*)-9a-Ethyl-3a-hydroxy-7-methyl-9-(nitromethyl)-2-phenyl-9,9a-dihydrochromeno[2,3-*c*]pyrrole-1,3(2*H*,3a*H*)-dione (**3ad**). From **1a** (21.6 mg, 0.10 mmol) and **2d** (17.8 mg, 0.12 mmol), purified by silica gel (200–300 mesh) column chromatography using ethyl acetate/petroleum ether (1:9) as eluent to obtain 34.5 mg (87% yield) compound **3ad** as a white solid, m.p. 188–189 °C. HPLC (Daicel Chiralpak AD-H, n-hexane/2-propanol = 90:10, flow rate 1.0 mL/min, detection at 254 nm): *t*_R_ = 15.8 min (major), *t*_R_ = 17.6 min (minor); 86% ee. [α]_D_^25^ = +82.7 (*c* = 1.73, CH_2_Cl_2_). ^1^H NMR (400 MHz, acetone-*d*_6_): 7.45–7.39 (m, 3H, ArH), 7.13 (dd, *J*_1_ = 1.6 Hz, *J*_2_ = 8.0 Hz, 1H, ArH), 6.94–6.88 (m, 4H, ArH), 5.21 (dd, *J*_1_ = 4.4 Hz, *J*_2_ = 12.4 Hz, 1H, CH_2_), 4.78 (dd, *J*_1_ = 11.4 Hz, *J*_2_ = 12.6 Hz, 1H, CH), 4.12 (dd, *J*_1_ = 4.4 Hz, *J*_2_ = 11.2 Hz, 1H, CH_2_), 2.29–2.21 (m, 1H, CH_2_), 3.84, 2.96 (s, 1H, OH), 2.24 (s, 3H, CH_3_), 2.15–2.08 (m, 1H, CH_2_), 1.12 (t, *J* = 7.4 Hz, 3H, CH_3_) ppm. ^13^C NMR (100 MHz, acetone-*d*_6_): 176.2, 173.3, 150.9, 135.1, 133.1, 132.5, 131.5, 131.0, 130.8, 128.2, 126.4, 119.4, 100.9, 76.5, 56.8, 41.7, 26.3, 21.6, 10.6 ppm. HRMS (ESI): *m*/*z* calcd. for C_21_H_21_N_2_O_6_ [M + H]^+^ 397.1394, found 397.1384.

(3a*S*,9*S*,9a*R*)-9a-Ethyl-3a-hydroxy-7-methoxy-9-(nitromethyl)-2-phenyl-9,9a-dihydrochromeno[2,3-*c*]pyrrole-1,3(2*H*,3a*H*)-dione (**3ae**). From **1a** (21.6 mg, 0.10 mmol) and **2e** (21.4 mg, 0.12 mmol), purified by silica gel (200–300 mesh) column chromatography using ethyl acetate: petroleum ether (1:9) as eluent to obtain 34.7 mg (84% yield) compound **3ae** as a white solid, m.p. 197–198 °C. HPLC (Daicel Chiralpak IB, n-hexane/2-propanol = 90:10, flow rate 1.0 mL/min, detection at 254 nm): *t*_R_ = 12.4 min (minor), *t*_R_ = 14.0 min (major); 92% ee. [α]_D_^25^ = +54.7 (*c* = 1.74, CH_2_Cl_2_). ^1^H NMR (400 MHz, acetone-*d*_6_): 8.17, 2.92 (s, 1H, OH), 7.45–7.39 (m, 3H, ArH), 6.96–6.92 (m, 3H, ArH), 6.88 (dd, *J*_1_ = 3.2 Hz, *J*_2_ = 8.8 Hz, 1H, ArH), 6.71 (d, *J* = 3.2 Hz, 1H, ArH), 5.22 (dd, *J*_1_ = 4.6 Hz, *J*_2_ = 13.0 Hz, 1H, CH_2_), 4.84–4.78 (dd, *J*_1_ = 11.6 Hz, *J*_2_ = 12.8 Hz, 1H, CH), 4.13 (dd, *J*_1_ = 4.4 Hz, *J*_2_ = 11.6 Hz, 1H, CH_2_), 3.73 (s, 3H, CH_3_), 2.30–2.20 (m, 1H, CH_2_), 2.14–2.08 (m, 1H, CH_2_), 1.12 (t, *J* = 7.4 Hz, 3H, CH_3_) ppm. ^13^C NMR (100 MHz, acetone-*d*_6_): 176.2, 173.3, 157.7, 146.6, 133.1, 131.0, 130.8, 128.3, 127.9, 120.5, 117.1, 116.2, 101.0, 76.3, 56.92, 56.85, 42.2, 26.2, 10.7 ppm. HRMS (ESI): *m*/*z* calcd. for C_21_H_21_N2O_7_ [M + H]^+^ 413.1343, found 413.1345.

(3a*S*,9*S*,9a*R*)-7-Chloro-9a-ethyl-3a-hydroxy-9-(nitromethyl)-2-phenyl-9,9a-dihydrochromeno[2,3-*c*]pyrrole-1,3(2*H*,3a*H*)-dione (**3ag**). From **1a** (21.6 mg, 0.10 mmol) and **2g** (20.0 mg, 0.12 mmol), purified by silica gel (200–300 mesh) column chromatography using ethyl acetate: petroleum ether (1:9) as eluent to obtain 37.1 mg (89% yield) compound **3ag** as a white solid, m.p. 208–210 °C. HPLC (Daicel Chiralpak AD-H, n-hexane/2-propanol = 85:15, flow rate 1.0 mL/min, detection at 254 nm): *t*_R_ = 11.0 min (major), *t*_R_ = 12.5 min (minor); 90% ee. [α]_D_^25^ = +34.1 (*c* = 1.86, CH_2_Cl_2_). ^1^H NMR (400 MHz, acetone-*d*_6_): 7.47–7.41 (m, 3H, ArH), 7.36 (dd, *J*_1_ = 2.4 Hz, *J*_2_ = 8.8 Hz, 1H, ArH), 7.22 (d, *J* = 2.4 Hz, 1H, ArH), 7.06 (d, *J* = 8.8 Hz, 1H, ArH), 6.98 (dd, *J*_1_ = 1.6 Hz, *J*_2_ = 8.0 Hz, 1H, ArH), 5.26 (dd, *J*_1_ = 4.4 Hz, *J*_2_ = 13.2 Hz, 1H, CH_2_), 4.81 (t, *J* = 12.2 Hz, 1H, CH), 4.20 (dd, *J*_1_ = 4.4 Hz, *J*_2_ = 11.2 Hz, 1H, CH_2_), 2.93 (s, 1H, OH), 2.32–2.23 (m, 1H, CH_2_), 2.18–2.09 (m, 1H, CH_2_), 1.12 (t, *J* = 7.4 Hz, 3H, CH_3_) ppm. ^13^C NMR (100 MHz, acetone-*d*_6_): 175.9, 172.8, 152.0, 132.9, 132.1, 131.1, 131.0, 130.0, 128.6, 128.2, 121.5, 101.3, 101.2, 76.0, 56.6, 41.1, 26.3, 10.6 ppm. HRMS (ESI): *m*/*z* calcd. for C_20_H_18_ClN_2_O_6_ [M + H]^+^ 417.0848, found 417.0840.

### 4.4. Procedure for the Scaled-Up Synthesis of Compound **3aa**

In a dried bottle, **1a** (0.216 g, 1.0 mmol), **2a** (0.368 g, 1.2 mmol), chiral organocatalyst **C3** (60.0 mg, 0.1 mmol, 10 mol%) and DCM (10.0 mL) were added. The mixture was stirred at −16 °C for 24 h. After completion of the reaction, the residue was purified by flash column chromatography on silica gel to obtain the pure product **3aa** (0.496 g, 85% yield).

### 4.5. Procedure for the Synthesis of Compound **4**

In a dried bottle, hydroxymaleimide **1b** (43.2 mg, 0.20 mmol), β-nitrostyrene **2h** (30.4 mg, 0.24 mmol), chiral organocatalyst **C3** (12.0 mg, 0.02 mmol, 10 mol%) and DCM (2.0 mL) were added. The mixture was stirred at −16 °C for 24 h. After completion of the reaction, the residue was purified by flash column chromatography on silica gel to afford the pure product **4** (15.1 mg, 20% yield, 2:1 dr) as a white solid, m.p. 182–191 °C. ^1^H NMR (400 MHz, acetone-*d*_6_): 7.40–7.34 (m, 3H, ArH), 7.22–7.17 (m, 2H, ArH), 7.09–7.04 (m, 2.7H, ArH), 6.96 (d, *J* = 8.8 Hz, 0.66 H, ArH), 6.73 (d, *J* = 8.8 Hz, 0.66 H, ArH), 5.48–5.41 (m, 1H, CH_2_), 5.36–5.18 (m, 1H, CH_2_), 4.22 (dd, *J*_1_ = 11.4 Hz, *J*_2_ = 4.0 Hz, 1H, CH), 3.85 (s, 2H, OCH_3_), 3.82 (s, 1H, O CH_3_), 2.37–2.14 (m, 2H, CH_2_), 1.10–1.05 (m, 3H, CH_3_) ppm. ^13^C NMR (100 MHz, acetone-*d*_6_): 200.4, 198.3, 175.9, 174.9, 162.22, 162.17, 160.6, 160.3, 136.3, 135.9, 131.21, 131.18, 131.0, 130.9, 130.7, 130.5, 129.41, 129.38, 125.0, 124.9, 116.3, 116.1, 76.5, 76.0, 59.2, 59.0, 56.95, 56.91, 49.3, 48.4, 27.2, 27.0, 10.2, 9.6 ppm. HRMS (ESI): *m*/*z* calcd. for C_21_H_21_N_2_O_6_ [M + H]^+^ 397.1394, found 397.1419.

## Figures and Tables

**Table 1:** Optimization of the reaction conditions ^a^.

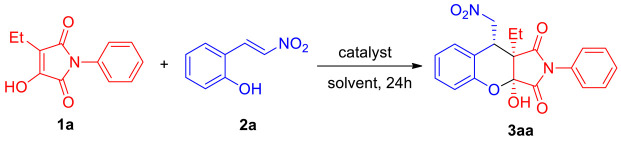						
Entry	Solvent	Catalyst	Temperate (°C)	Yield ^b^ (%)	dr ^c^	ee ^d^ (%)
1	CH_2_Cl_2_	**C1**	rt	82	>20:1	53
2	CH_2_Cl_2_	**C1**	−16	80	>20:1	87
3	CH_2_Cl_2_	**C2**	rt	85	>20:1	55
4	CH_2_Cl_2_	**C2**	−16	83	>20:1	73
5	CH_2_Cl_2_	**C3**	−16	88	>20:1	96
6	CH_2_Cl_2_	**C4**	−16	79	>20:1	77
7	CH_2_Cl_2_	**C5**	−16	77	>20:1	95
8	CH_2_Cl_2_	**C6**	−16	89	>20:1	59
9	CH_2_Cl_2_	**C7**	−16	82	>20:1	81
10	CH_2_Cl_2_	**C8**	−16	78	>20:1	73
11	CH_2_Cl_2_	**C9**	−16	90	16:1	−69
12	CH_2_Cl_2_	**C10**	−16	81	9:1	59
13	DCE	**C3**	−16	87	>20:1	93
14	Toluene	**C3**	−16	80	>20:1	85
15	MeCN	**C3**	−16	89	>20:1	79
16	CHCl_3_	**C3**	−16	83	>20:1	87
17	THF	**C3**	−16	trace	–	–
18 ^e^	CH_2_Cl_2_	**C3**	−16	86	>20:1	95
19 ^f^	CH_2_Cl_2_	**C3**	−16	80	>20:1	89

^a^ Unless otherwise specified, the reactions were carried out with **1a** (0.10 mmol), **2a** (0.12 mmol) and catalyst (10 mol%) in solvent (1.0 mL) for 24 h. ^b^ Isolated yield after column chromatography purification. ^c^ Determined by ^1^H NMR analysis. ^d^ The enantiomeric excess (ee) was determined by HPLC analysis. ^e^ 5 mol% catalyst was used. ^f^ 2.5 mol% catalyst was used.

## Data Availability

Not applicable.

## Supplementary Materials

The following supporting information can be downloaded at: https://www.mdpi.com/article/10.3390/molecules27165081/s1, Spectroscopic data (^1^H and ^13^C NMR), X-ray single-crystal data and chiral HPLC chromatograms for all new compounds **3**.

## Sample Availability

Samples of the compounds **3** are available from the authors.
