# Supplementary material for: Enantioselective Michael/Hemiketalization Cascade Reactions between Hydroxymaleimides and 2-Hydroxynitrostyrenes for the Construction of Chiral Chroman-Fused Pyrrolidinediones
Source: Molecules. 2022 Aug 10;27(16):5081. doi: 10.3390/molecules27165081 (PMC9414856; doi:10.3390/molecules27165081)
Supplement: Supplementary file 1 [file molecules-27-05081-s001.zip › molecules-1849391-supplementary.pdf]

Enantioselective Michael/Hemiketalization Cascade Reactions Between Hydroxymaleimides and 2-Hydroxynitrostyrenes for the Construction of Chiral Chroman-fused Pyrrolidinediones

Dong-Hua Xie, Cheng Niu, Da-Ming Du*

School of Chemistry and Chemical Engineering, Beijing Institute of Technology,

Beijing 100081, People's Republic of China

E-mail: dudm@bit.edu.cn

Supporting Information

Contents

1. Copies of ^{1}H and ^{13}C NMR spectra of new compounds……………...............................S1
2. X-ray single-crystal data for product 3ca…………………….…..…………………… .S43
3. Copies of HPLC chromatograms…………………….…..…………………………….. S44

1. **Copies of ^{1}H and ^{13}C NMR spectra of new compounds**

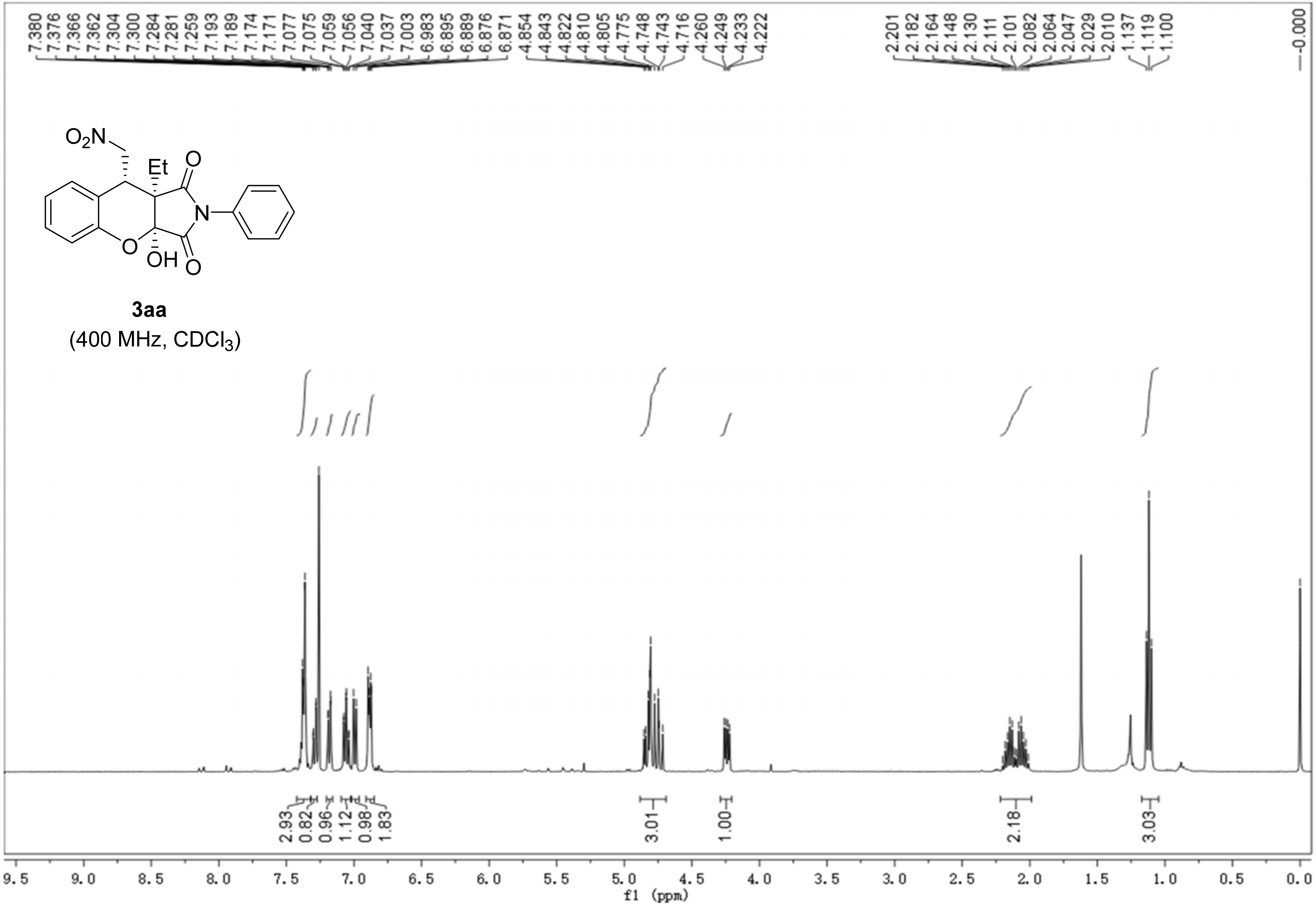

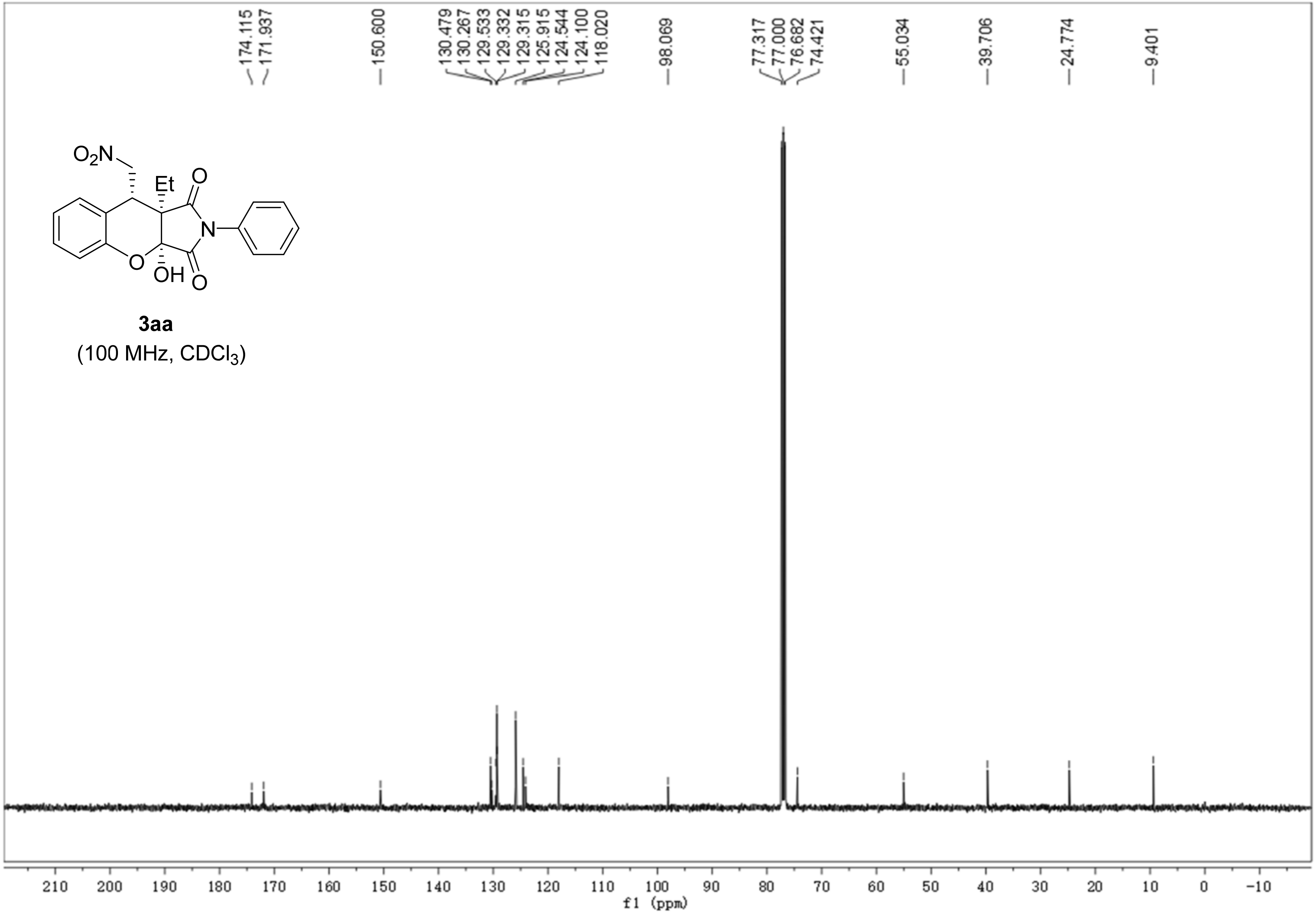
174.115
171.937
150.600
130.479
130.267
129.533
129.332
129.315
125.915
124.544
124.100
118.020
98.069
77.317
77.000
76.682
74.421
55.034
39.706
24.774
9.401
O_2N
Et
O
N
OH
3aa
(100 MHz, $CDCl_3$)
210 200 190 180 170 160 150 140 130 120 110 100 90 80 70 60 50 40 30 20 10 0 −10
f1 (ppm)

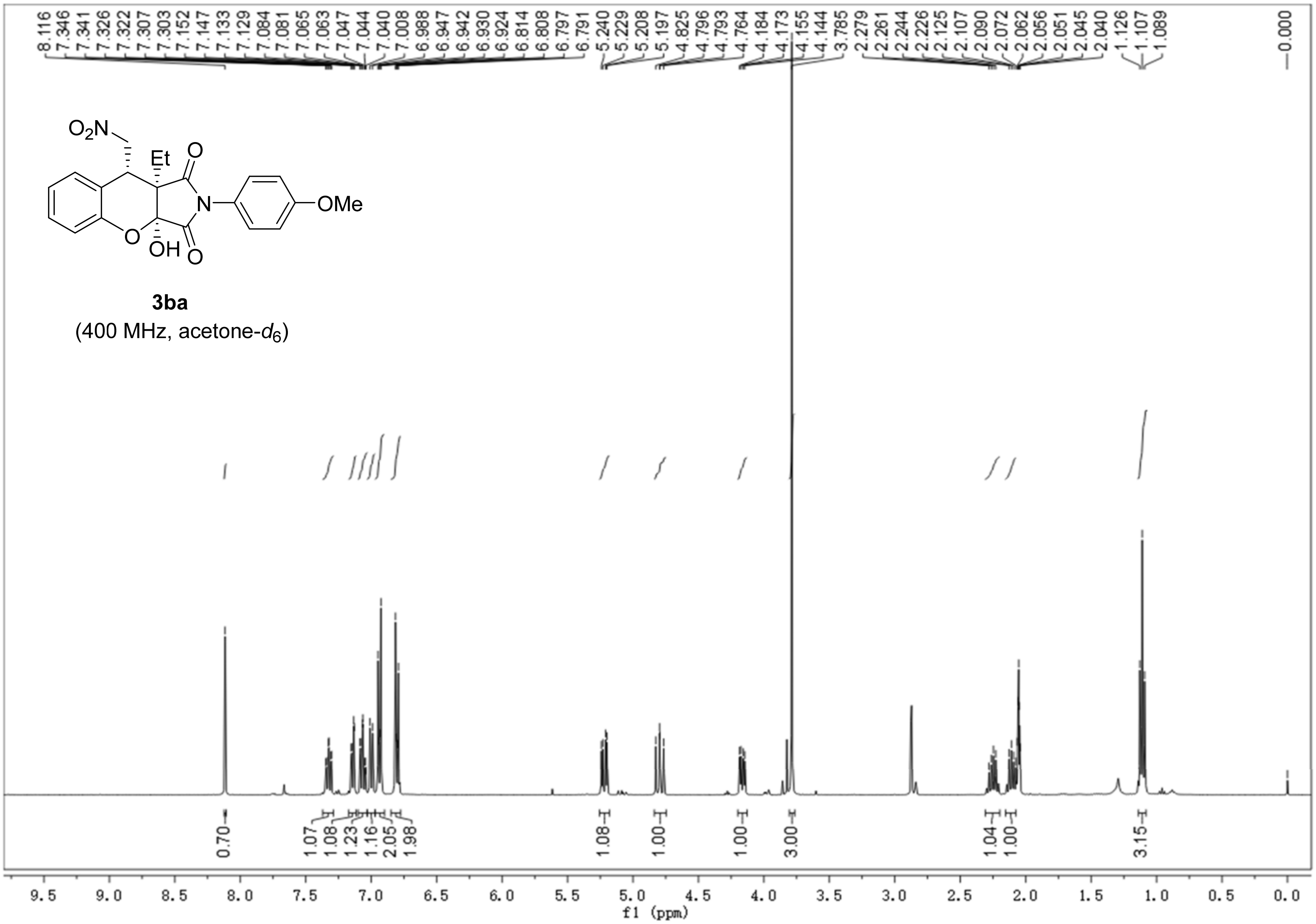

3ba

(400 MHz, acetone-d_6)

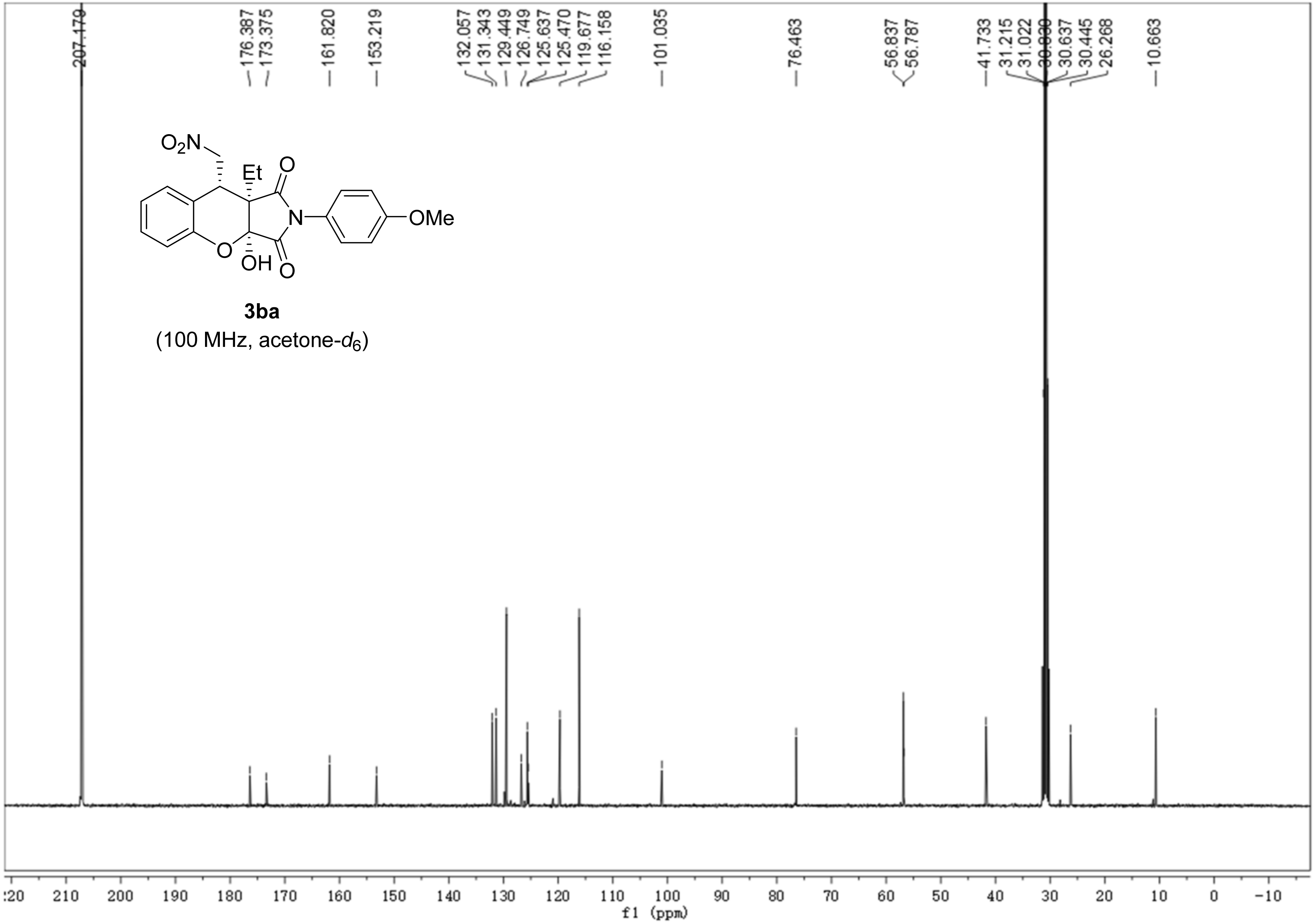
207.179
176.387
173.375
161.820
153.219
132.057
131.343
129.449
126.749
125.637
125.470
119.677
116.158
101.035
76.463
56.837
56.787
41.733
31.215
31.022
30.637
30.445
26.268
10.663
O2N
Et
O
N
OMe
O
OH
O
3ba
(100 MHz, acetone-d6)
210 200 190 180 170 160 150 140 130 120 110 100 90 80 70 60 50 40 30 20 10 0 −10
f1 (ppm)

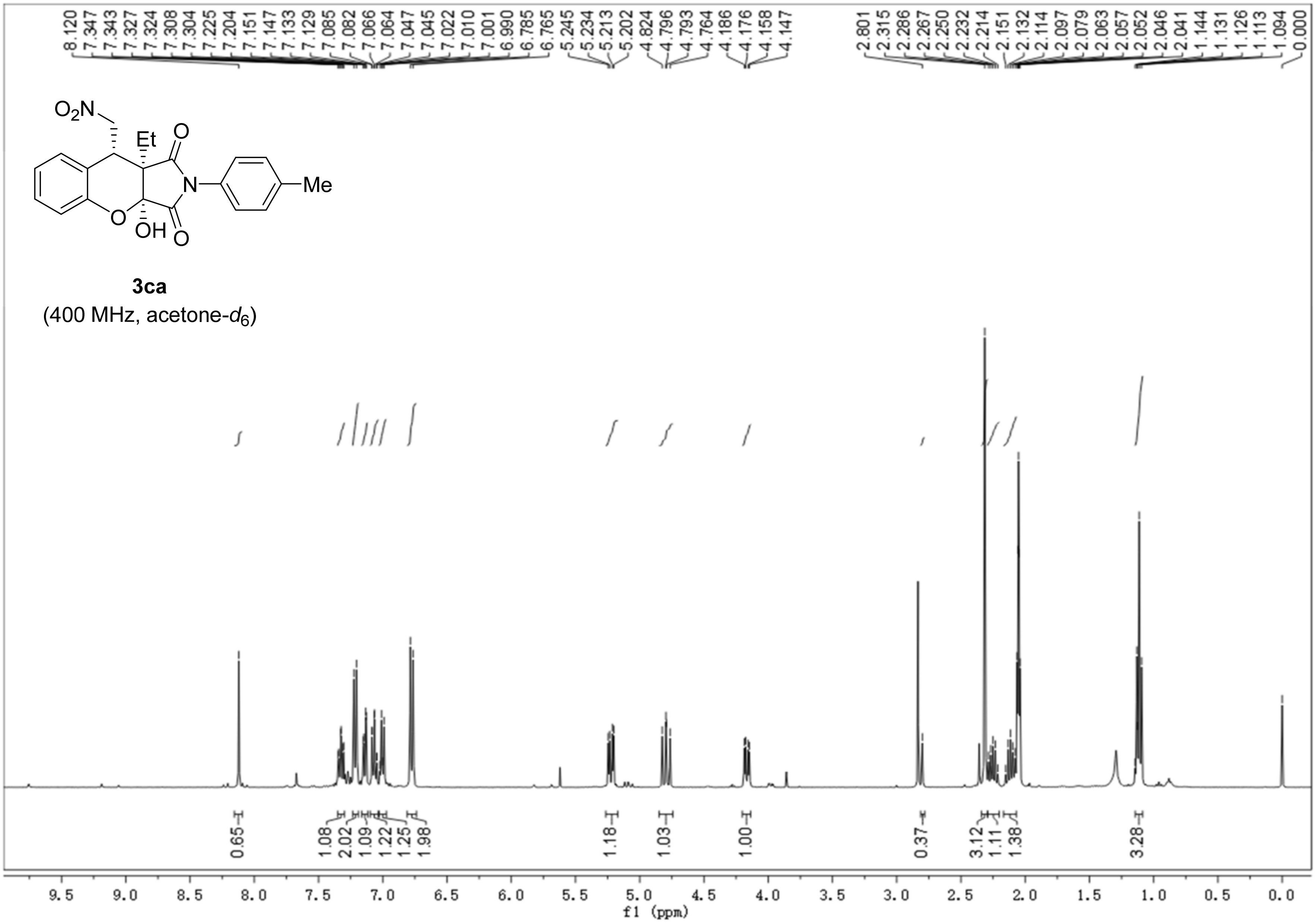

3ca
(400 MHz, acetone-d_6)

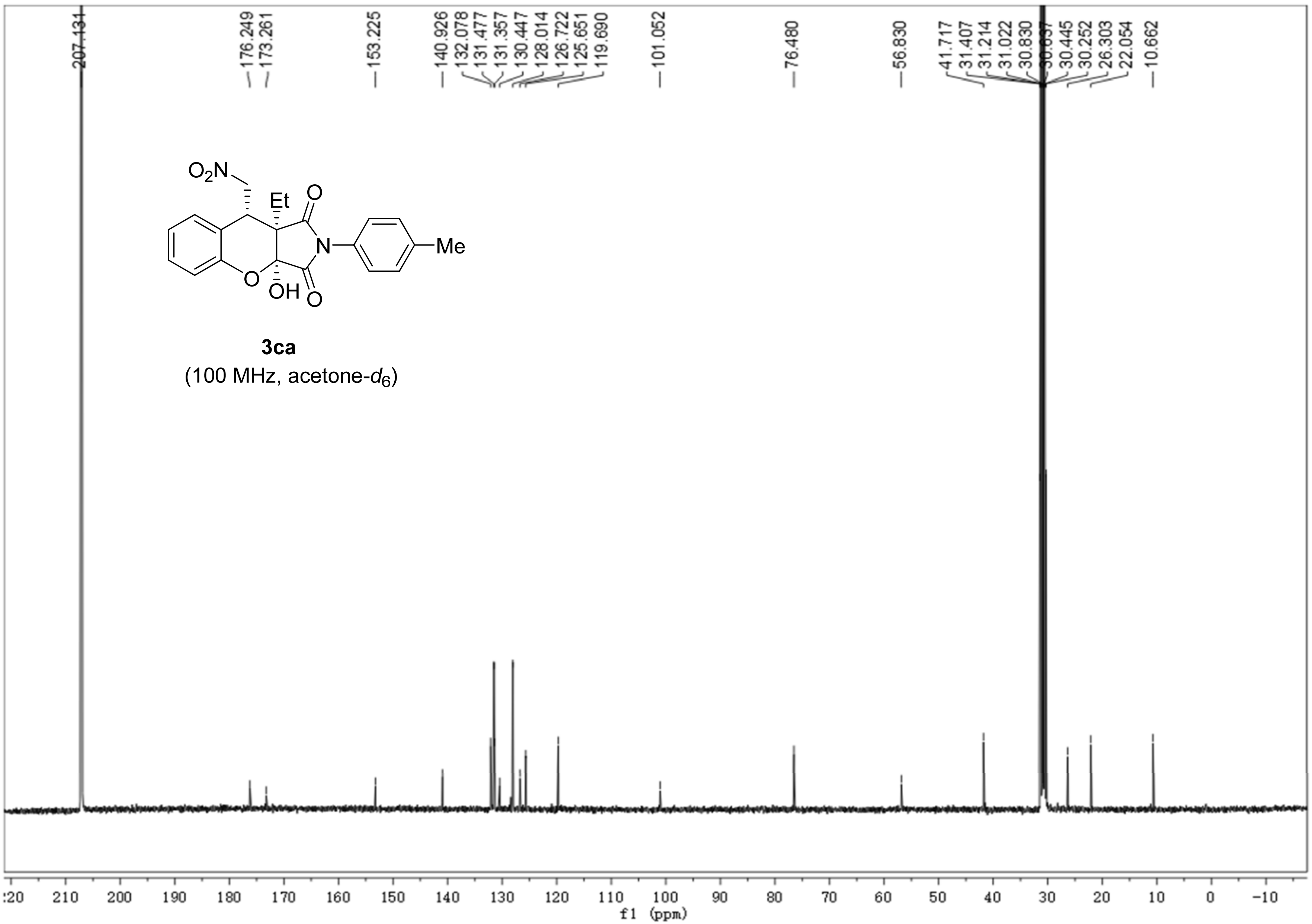

207.131
176.249
173.261
153.225
140.926
132.078
131.477
131.357
130.447
128.014
126.722
125.651
119.690
101.052
76.480
56.830
41.717
31.407
31.214
31.022
30.830
30.637
30.445
30.252
26.303
22.054
10.662
O2N
Et
O
N
Me
O
OH
O
3ca
(100 MHz, acetone-d6)
f1 (ppm)

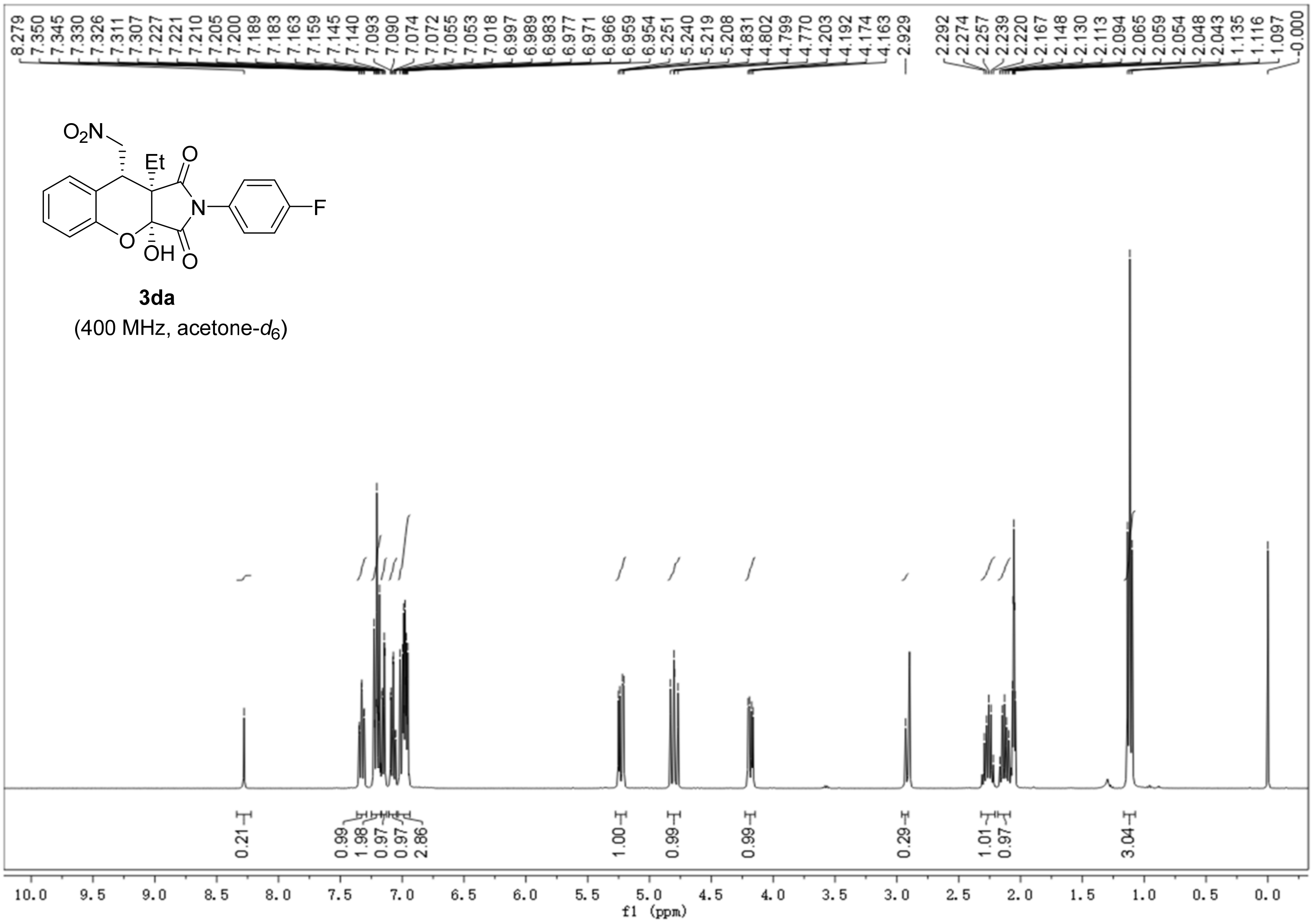

3da

(400 MHz, acetone-d_6)

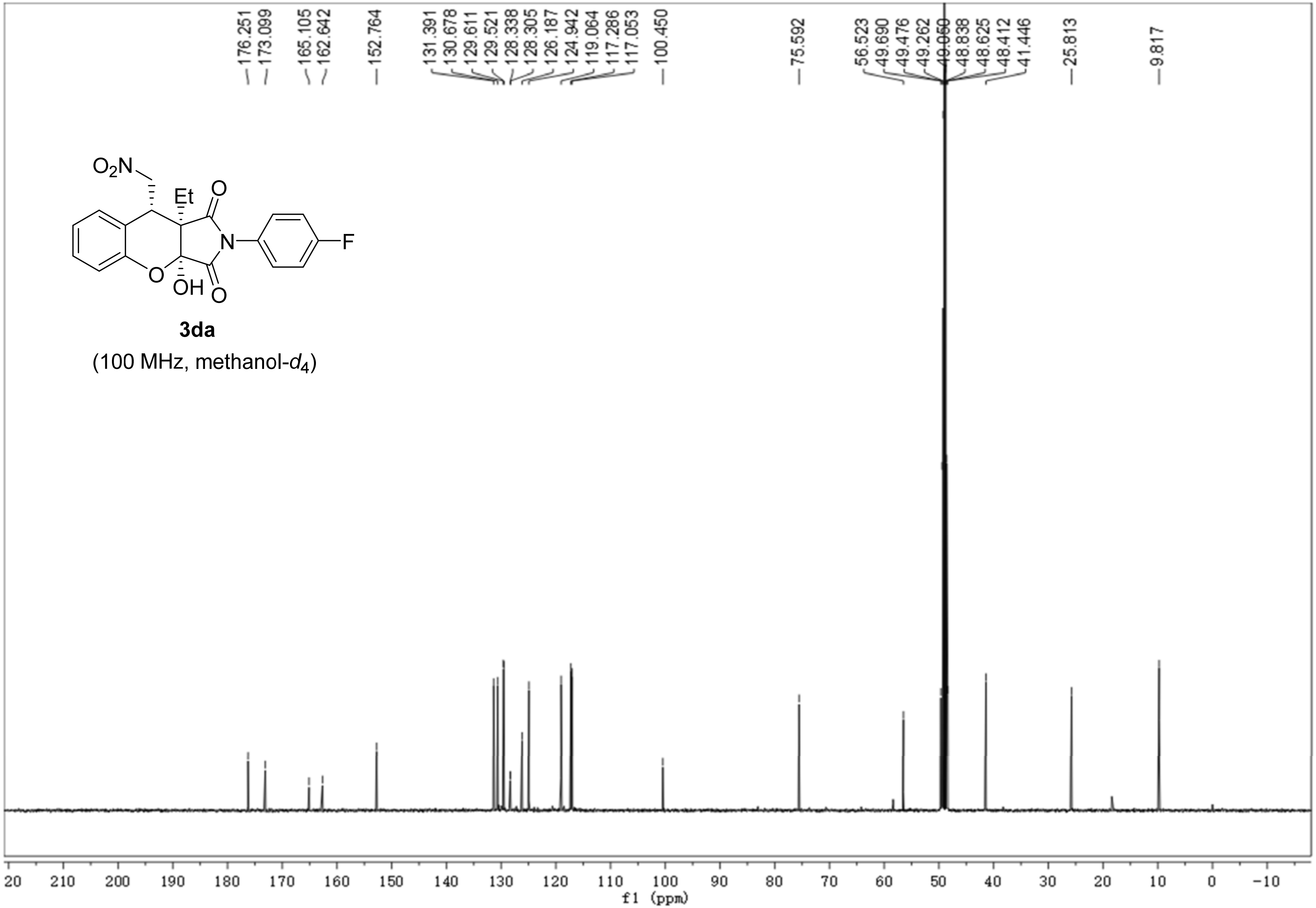
176.251
173.099
165.105
162.642
152.764
131.391
130.678
129.611
129.521
128.338
128.305
126.187
124.942
119.064
117.286
117.053
100.450
75.592
56.523
49.690
49.476
49.262
49.050
48.838
48.625
48.412
41.446
25.813
9.817
O_2N
Et
O
N
F
O
OH
O
3da
(100 MHz, methanol-d_4)
f1 (ppm)

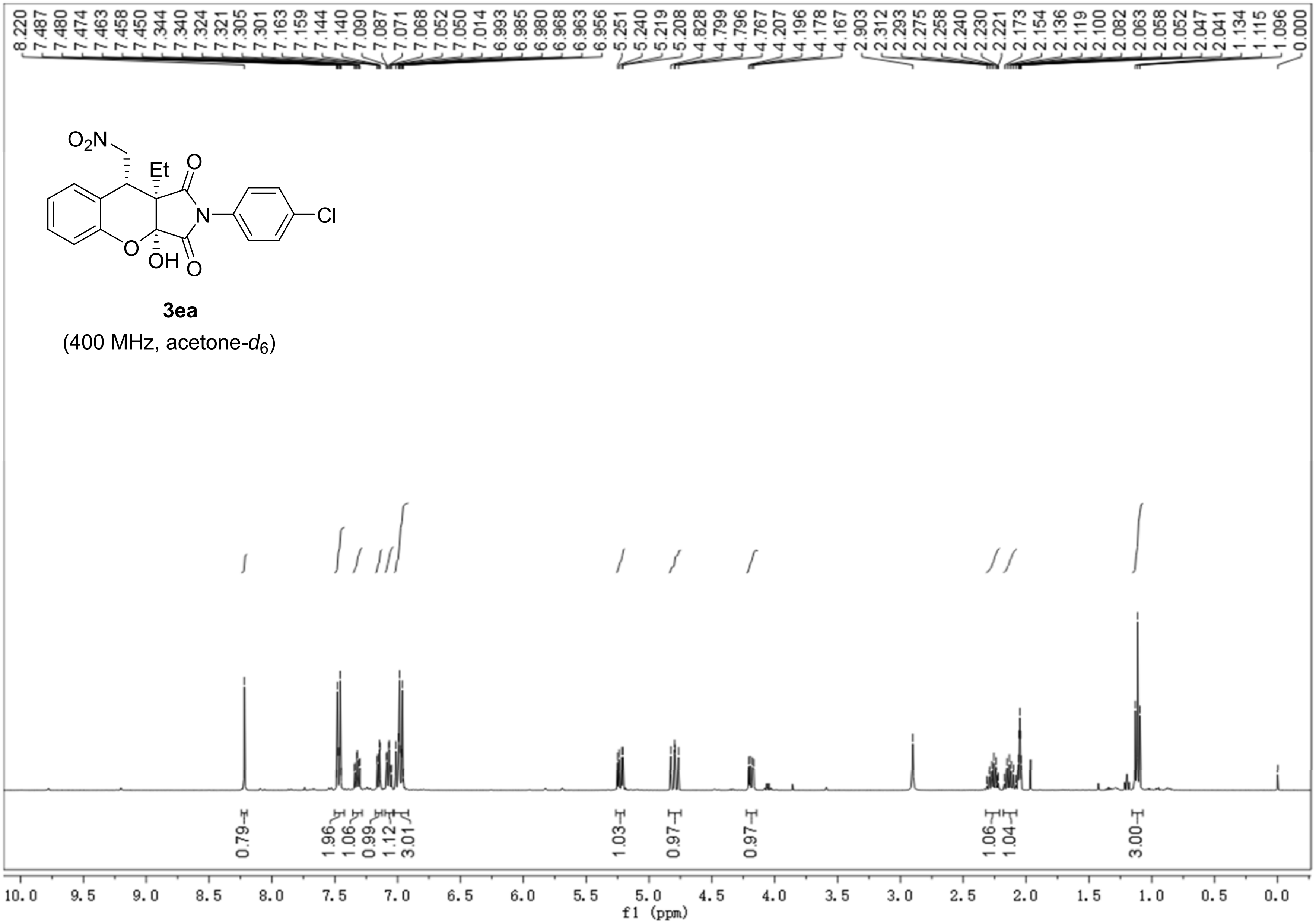
8.220 7.487 7.480 7.474 7.463 7.458 7.450 7.344 7.340 7.324 7.321 7.305 7.301 7.163 7.159 7.144 7.140 7.090 7.087 7.071 7.068 7.052 7.050 7.014 6.993 6.985 6.980 6.968 6.963 6.956 5.251 5.240 5.219 5.208 4.828 4.799 4.796 4.767 4.207 4.196 4.178 4.167 2.903 2.312 2.293 2.275 2.258 2.240 2.230 2.221 2.173 2.154 2.136 2.119 2.100 2.082 2.063 2.058 2.052 2.047 2.041 1.134 1.115 1.096 0.000
O2N
Et
O
N
Cl
O
OH
O
3ea
(400 MHz, acetone-d6)
0.79
1.96
1.06
0.99
1.12
3.01
1.03
0.97
0.97
1.06
1.04
3.00
10.0 9.5 9.0 8.5 8.0 7.5 7.0 6.5 6.0 5.5 5.0 4.5 4.0 3.5 3.0 2.5 2.0 1.5 1.0 0.5 0.0
f1 (ppm)

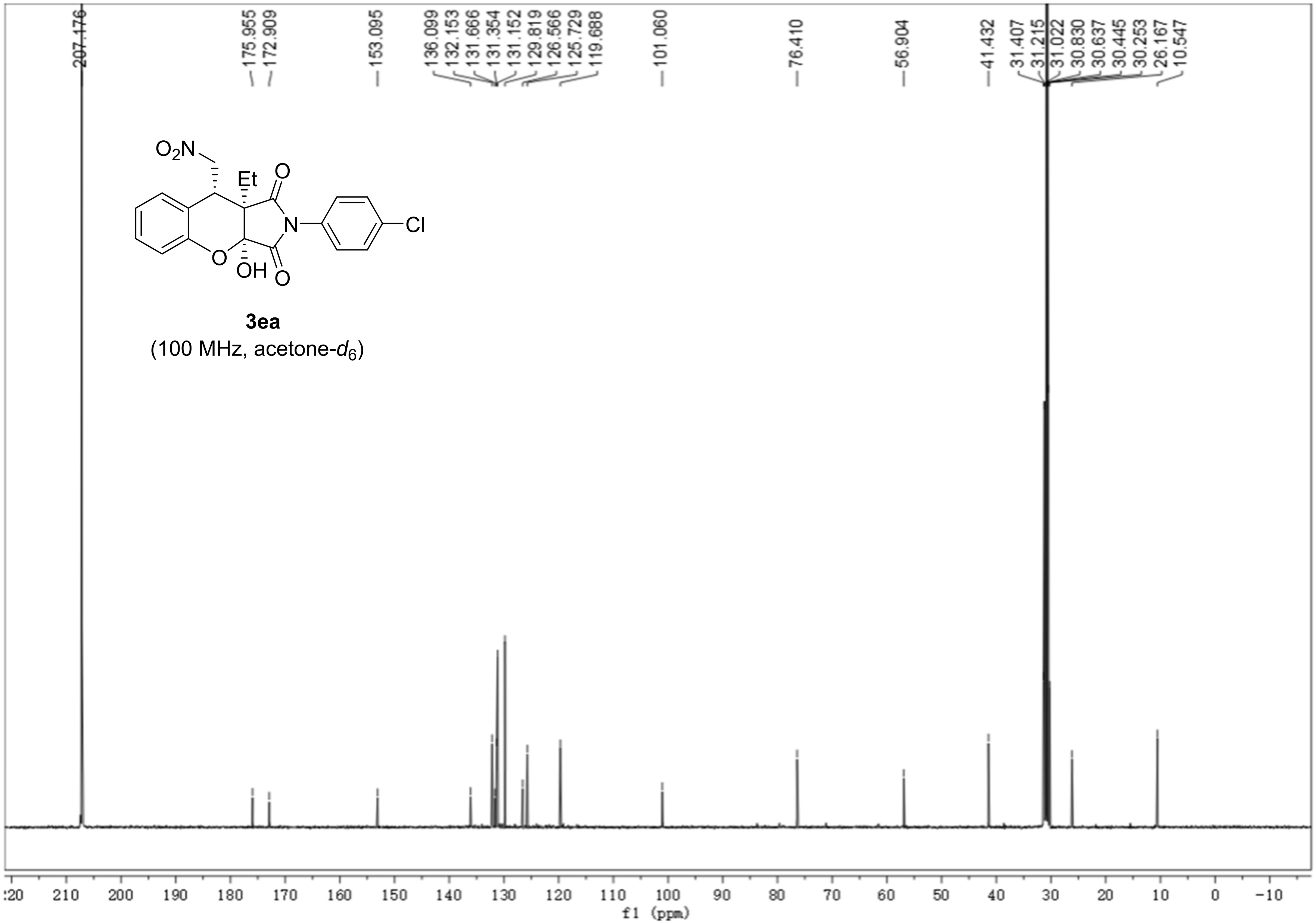
207.176
175.955
172.909
153.095
136.099
132.153
131.666
131.354
131.152
129.819
126.566
125.729
119.688
101.060
76.410
56.904
41.432
31.407
31.215
31.022
30.830
30.637
30.445
30.253
26.167
10.547
O_2N
Et
O
N
Cl
O
OH
O
3ea
(100 MHz, acetone-d_6)
f1 (ppm)

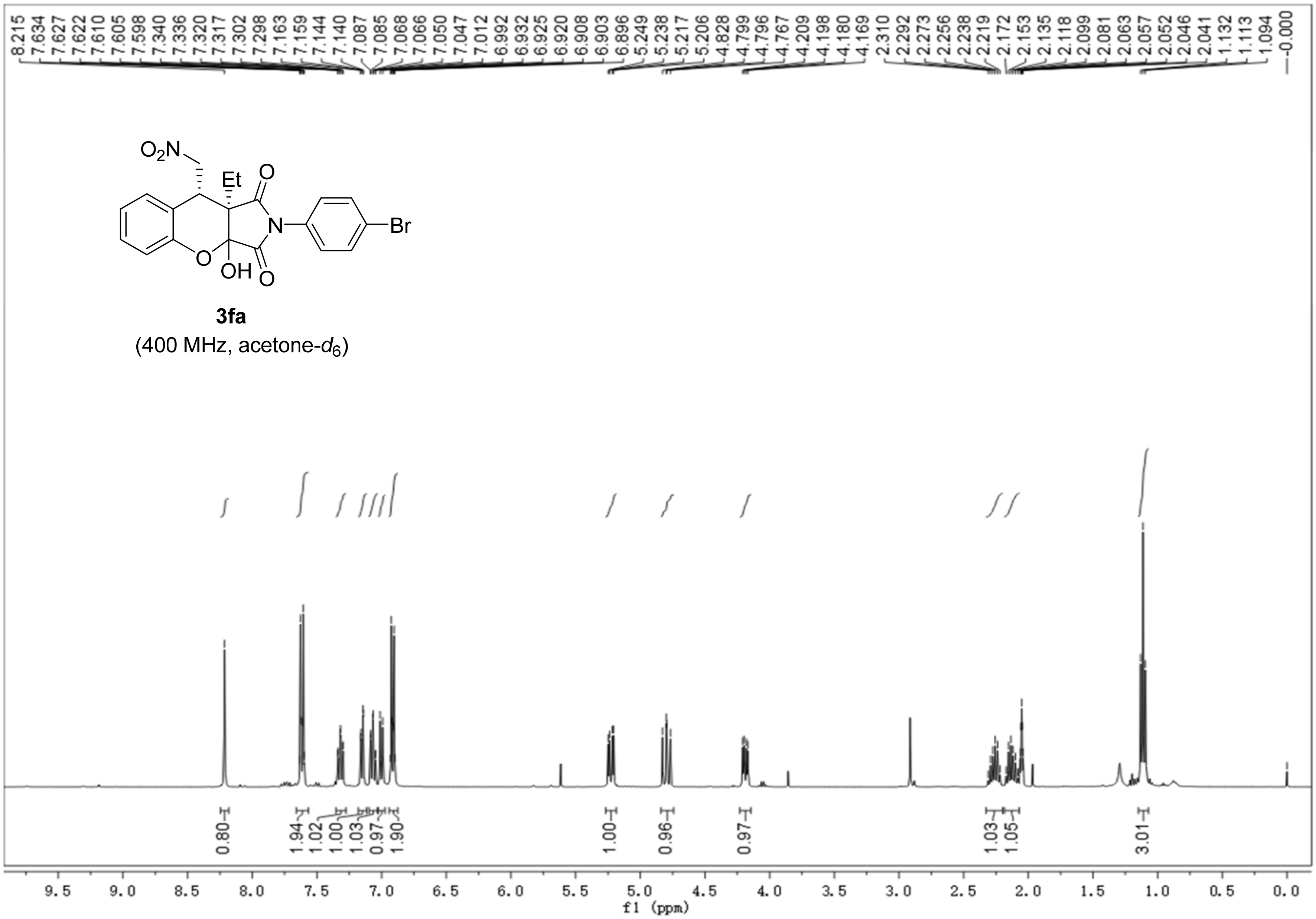
8.215
7.634
7.627
7.622
7.610
7.605
7.598
7.340
7.336
7.320
7.317
7.302
7.298
7.163
7.159
7.144
7.140
7.087
7.085
7.068
7.066
7.050
7.047
7.012
6.992
6.932
6.925
6.920
6.908
6.903
6.896
5.249
5.238
5.217
5.206
4.828
4.799
4.796
4.767
4.209
4.198
4.180
4.169
2.310
2.292
2.273
2.256
2.238
2.219
2.172
2.153
2.135
2.118
2.099
2.081
2.063
2.057
2.052
2.046
2.041
1.132
1.113
1.094
-0.000
O_2N
Et
O
N
Br
O
OH
O
3fa
(400 MHz, acetone-d_6)
0.80
1.94
1.02
1.00
1.03
0.97
1.90
1.00
0.96
0.97
1.03
1.05
3.01
9.5
9.0
8.5
8.0
7.5
7.0
6.5
6.0
5.5
5.0
4.5
4.0
3.5
3.0
2.5
2.0
1.5
1.0
0.5
0.0
f1 (ppm)

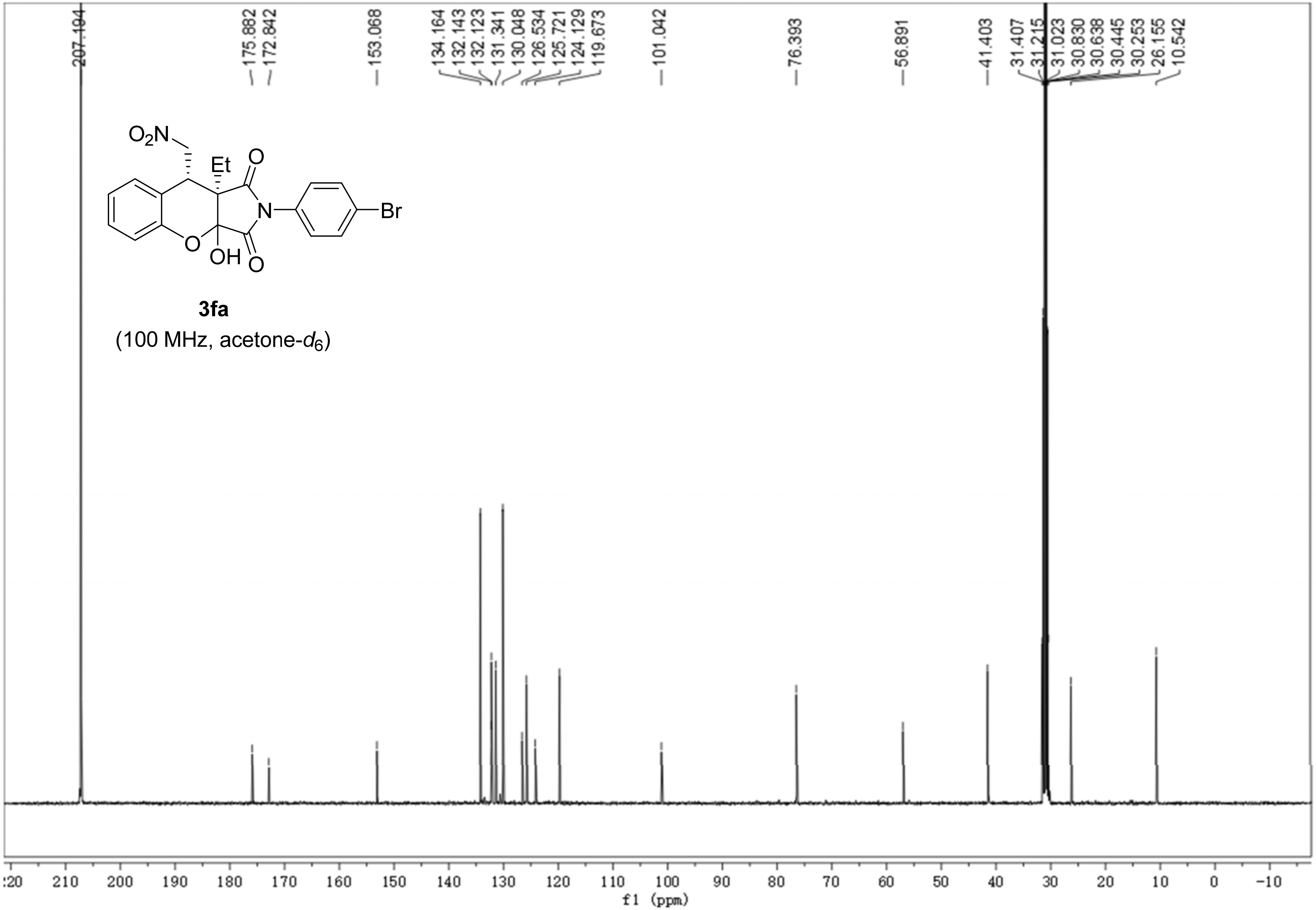
207.194
175.882
172.842
153.068
134.164
132.143
132.123
131.341
130.048
126.534
125.721
124.129
119.673
101.042
76.393
56.891
41.403
31.407
31.215
31.023
30.830
30.638
30.445
30.253
26.155
10.542
O2N
Et
O
N
Br
O
OH
O
3fa
(100 MHz, acetone-d6)
f1 (ppm)

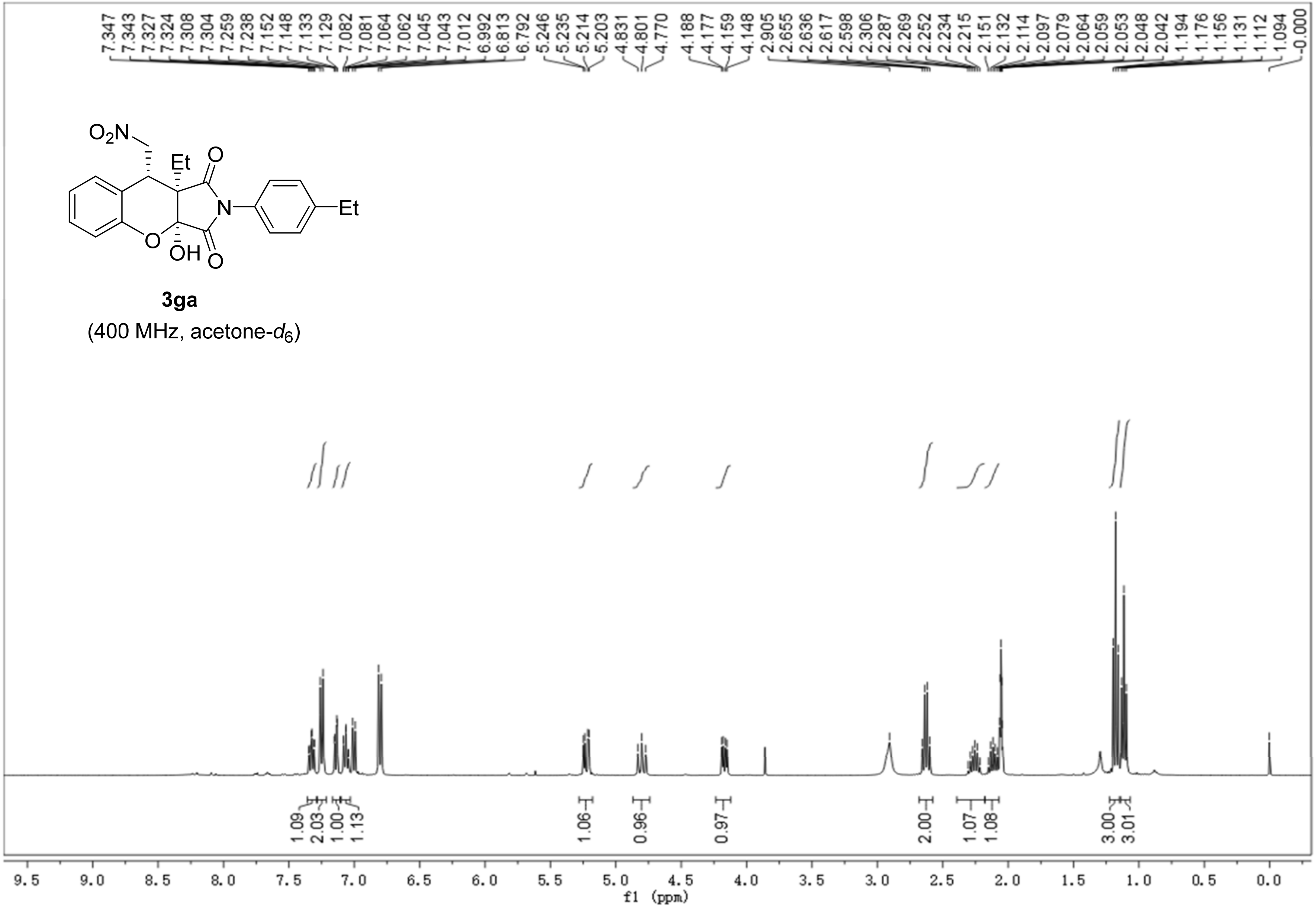

3ga

(400 MHz, acetone-d_6)

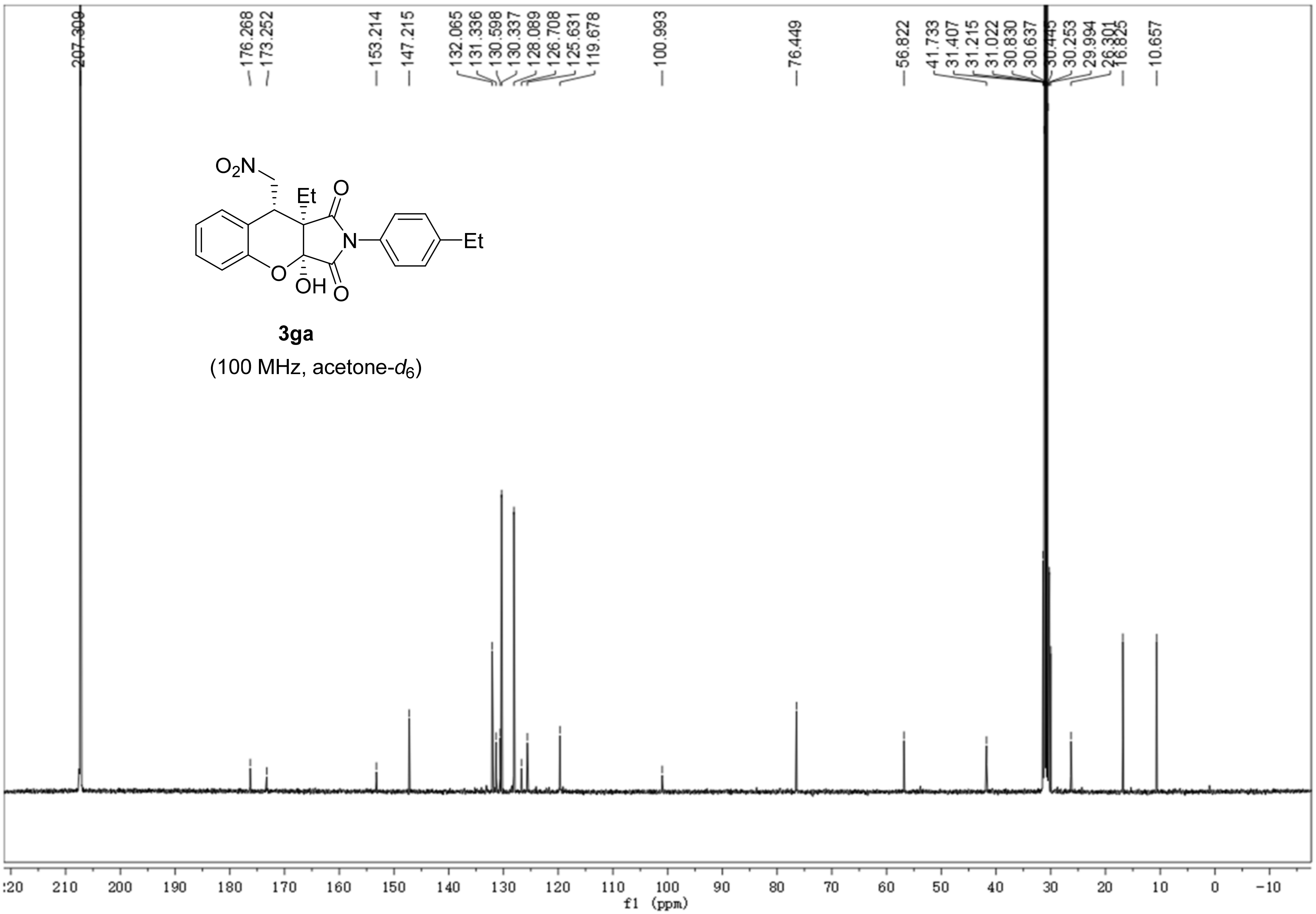

207.309
176.268
173.252
153.214
147.215
132.065
131.336
130.598
130.337
128.089
126.708
125.631
119.678
100.993
76.449
56.822
41.733
31.407
31.215
31.022
30.830
30.637
30.445
30.253
29.994
26.301
16.825
10.657
O_2N
Et
O
N
Et
O
OH
O
3ga
(100 MHz, acetone-d_6)
f1 (ppm)

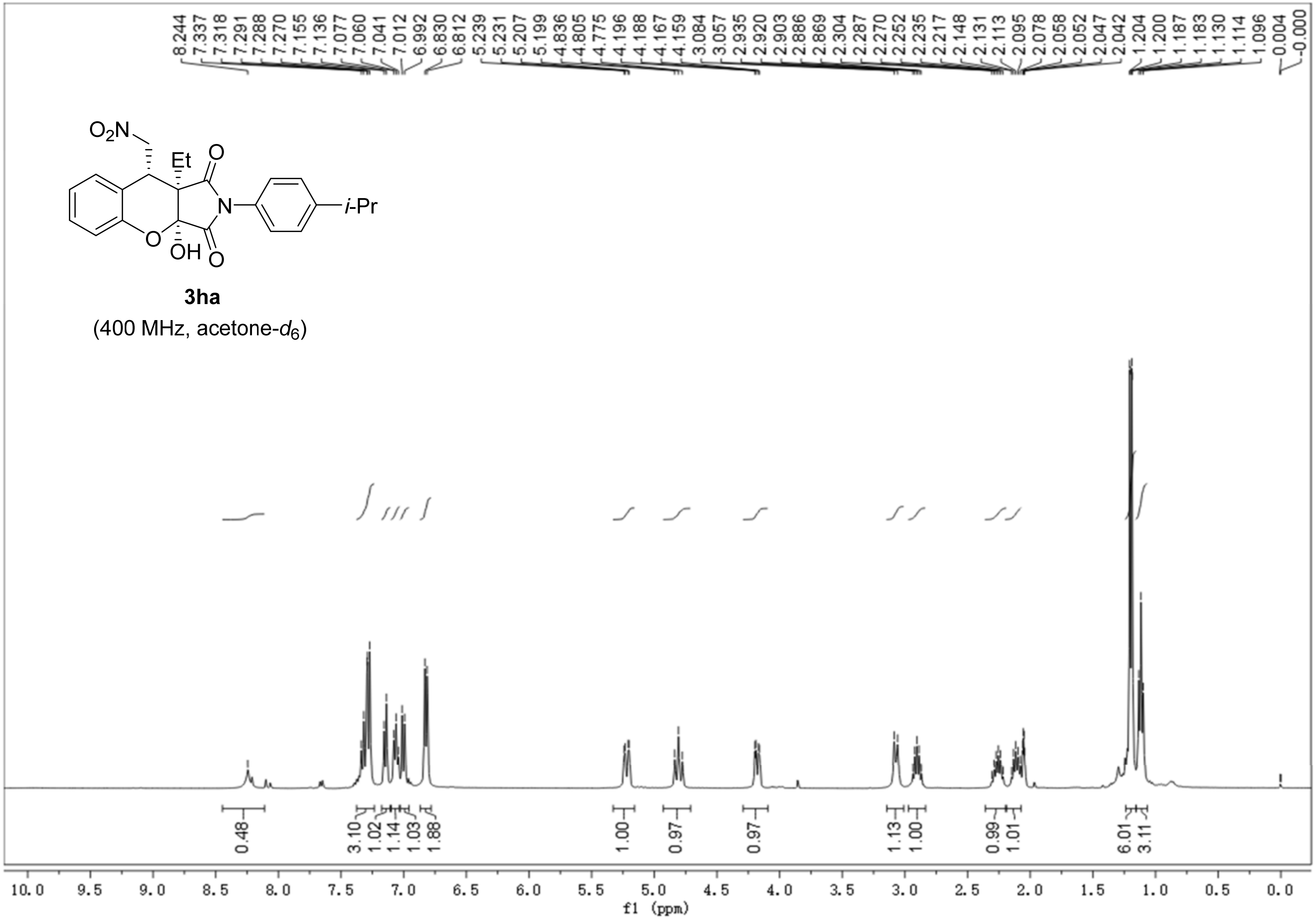

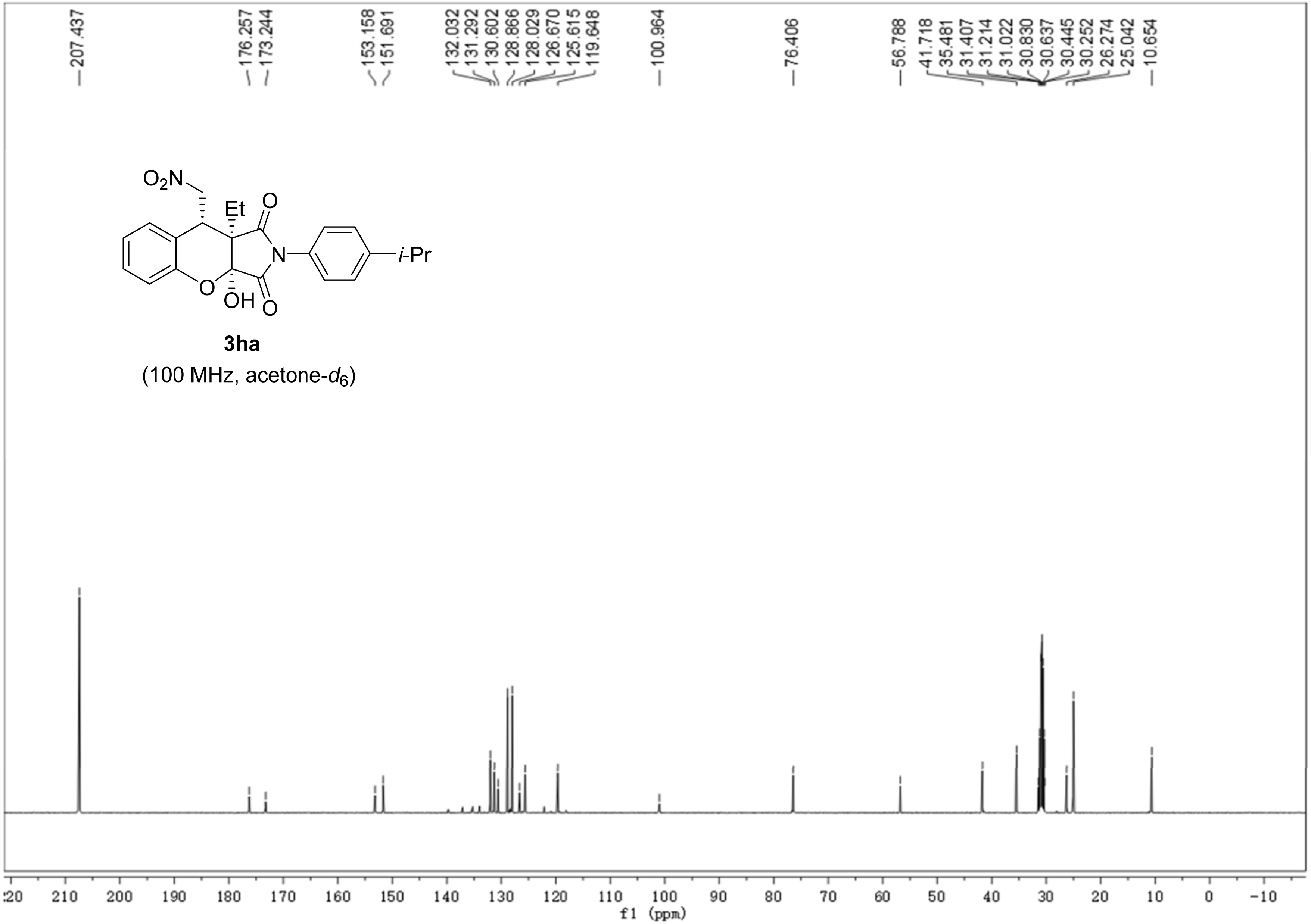

3ha

(100 MHz, acetone-d_6)

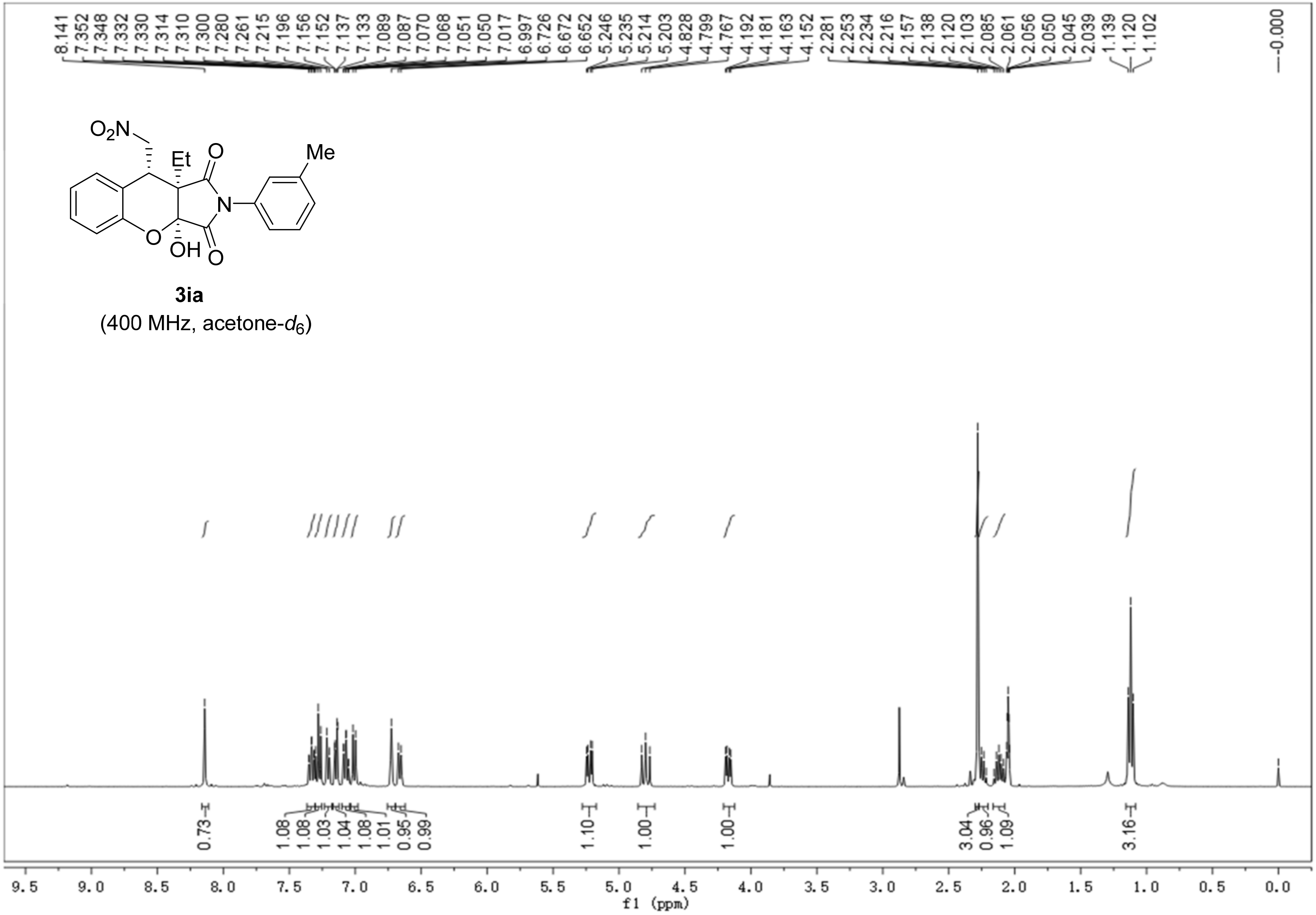

3ia
(400 MHz, acetone-d_6)

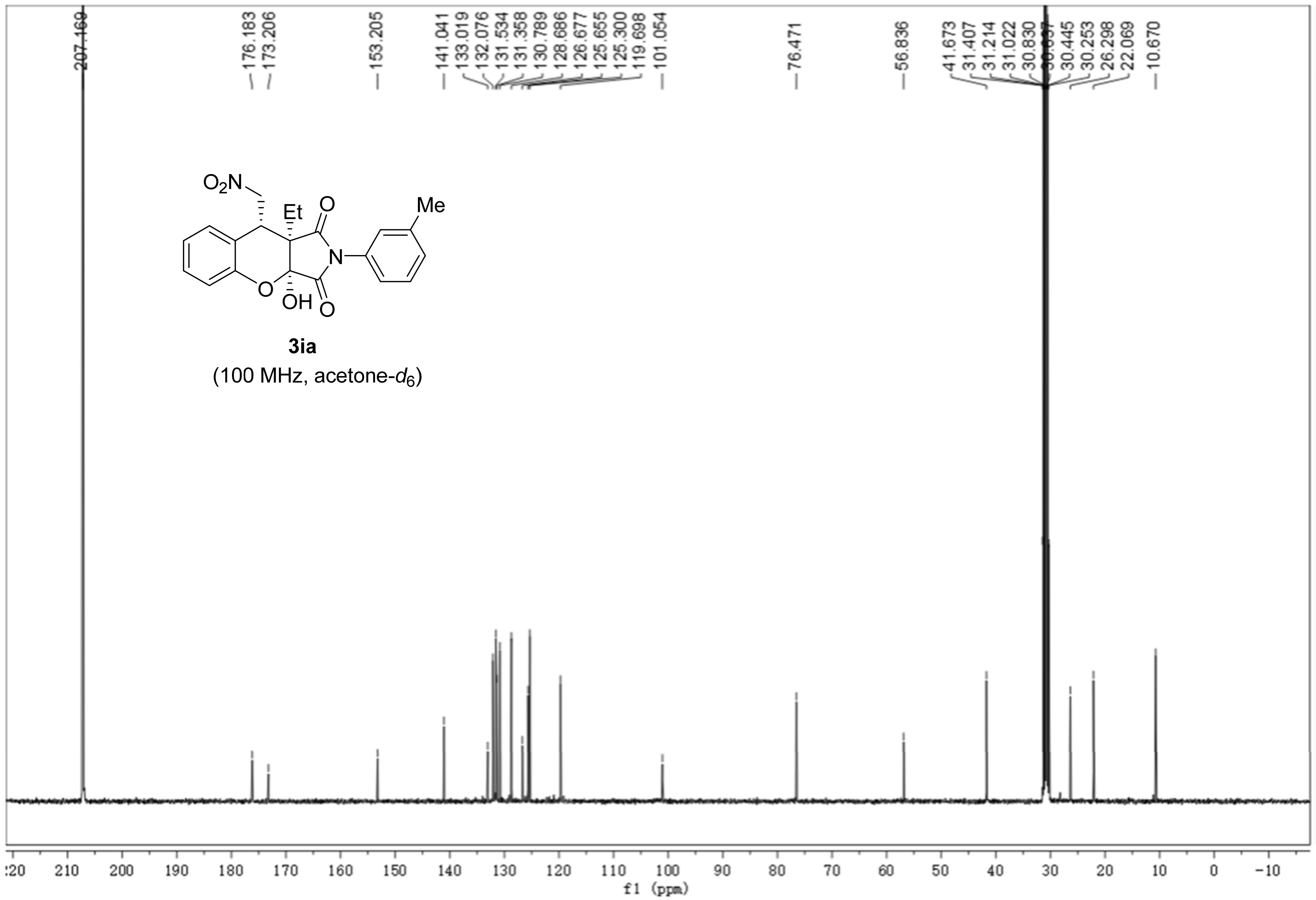

207.169
176.183
173.206
153.205
141.041
133.019
132.076
131.534
131.358
130.789
128.686
126.677
125.655
125.300
119.698
101.054
76.471
56.836
41.673
31.407
31.214
31.022
30.830
30.445
30.253
26.298
22.069
10.670
O_2N
Et
Me
N
O
OH
3ia
(100 MHz, acetone-d_6)
f1 (ppm)

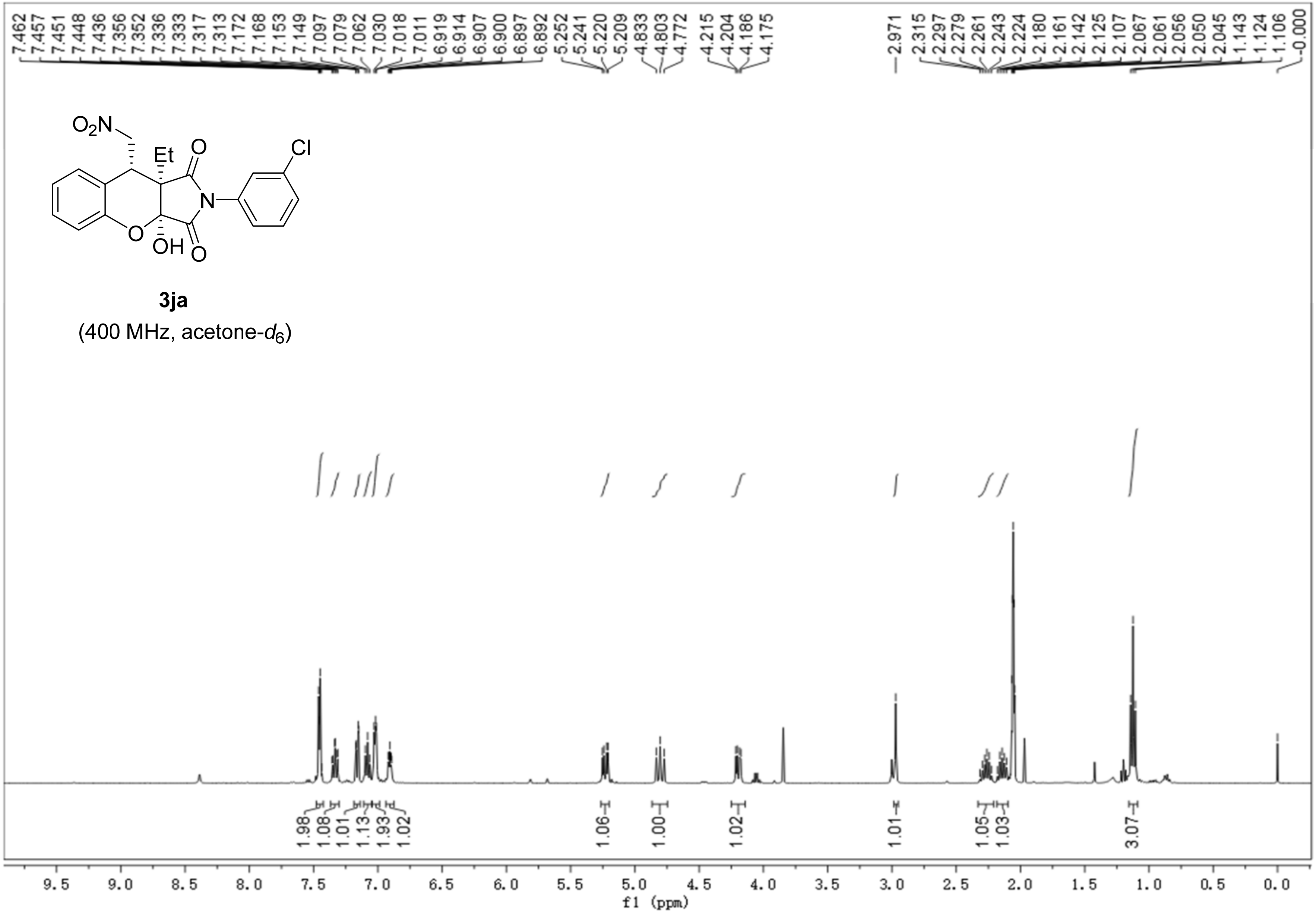

3ja
(400 MHz, acetone-d_6)
f1 (ppm)

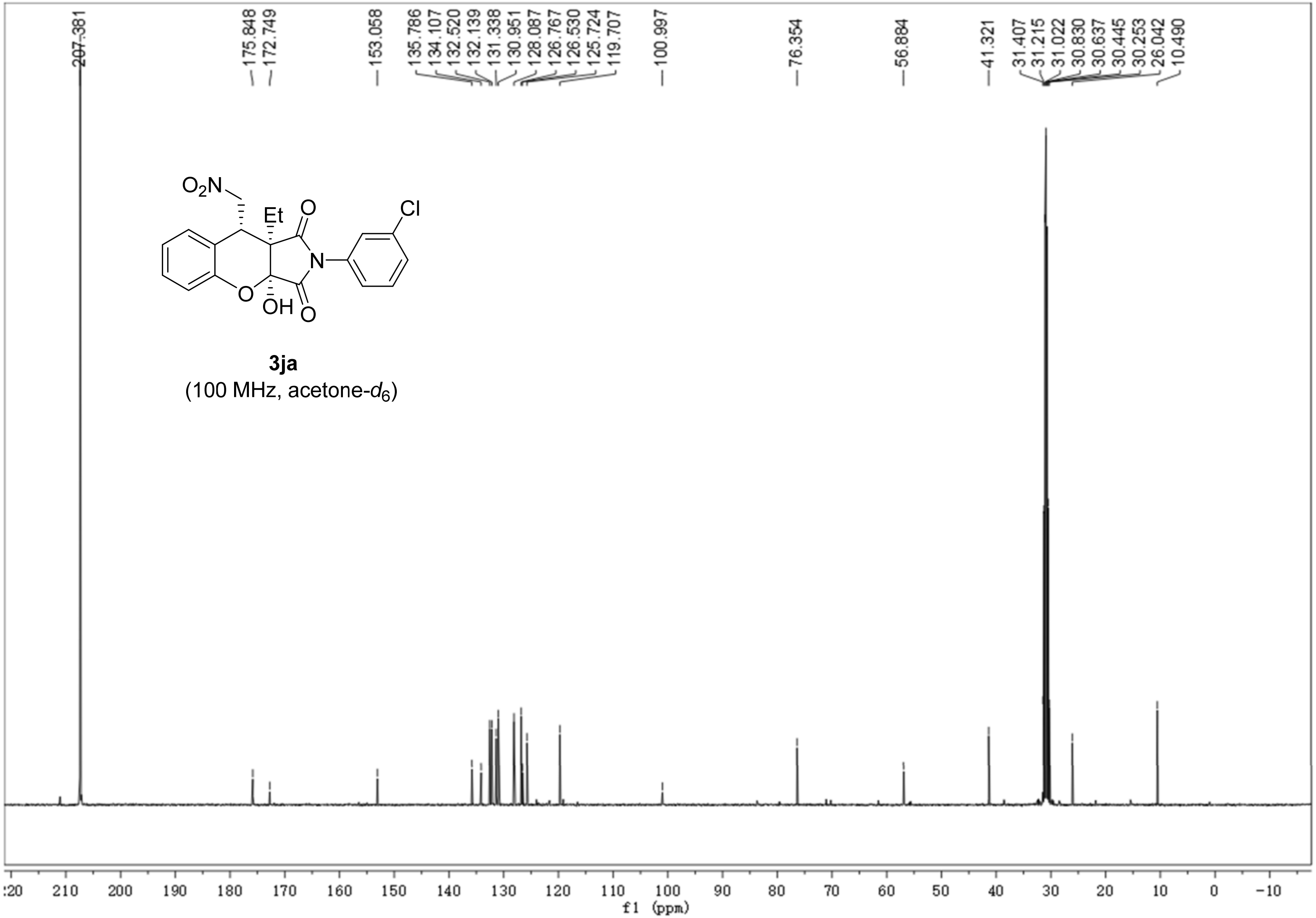

3ja
(100 MHz, acetone-d_6)

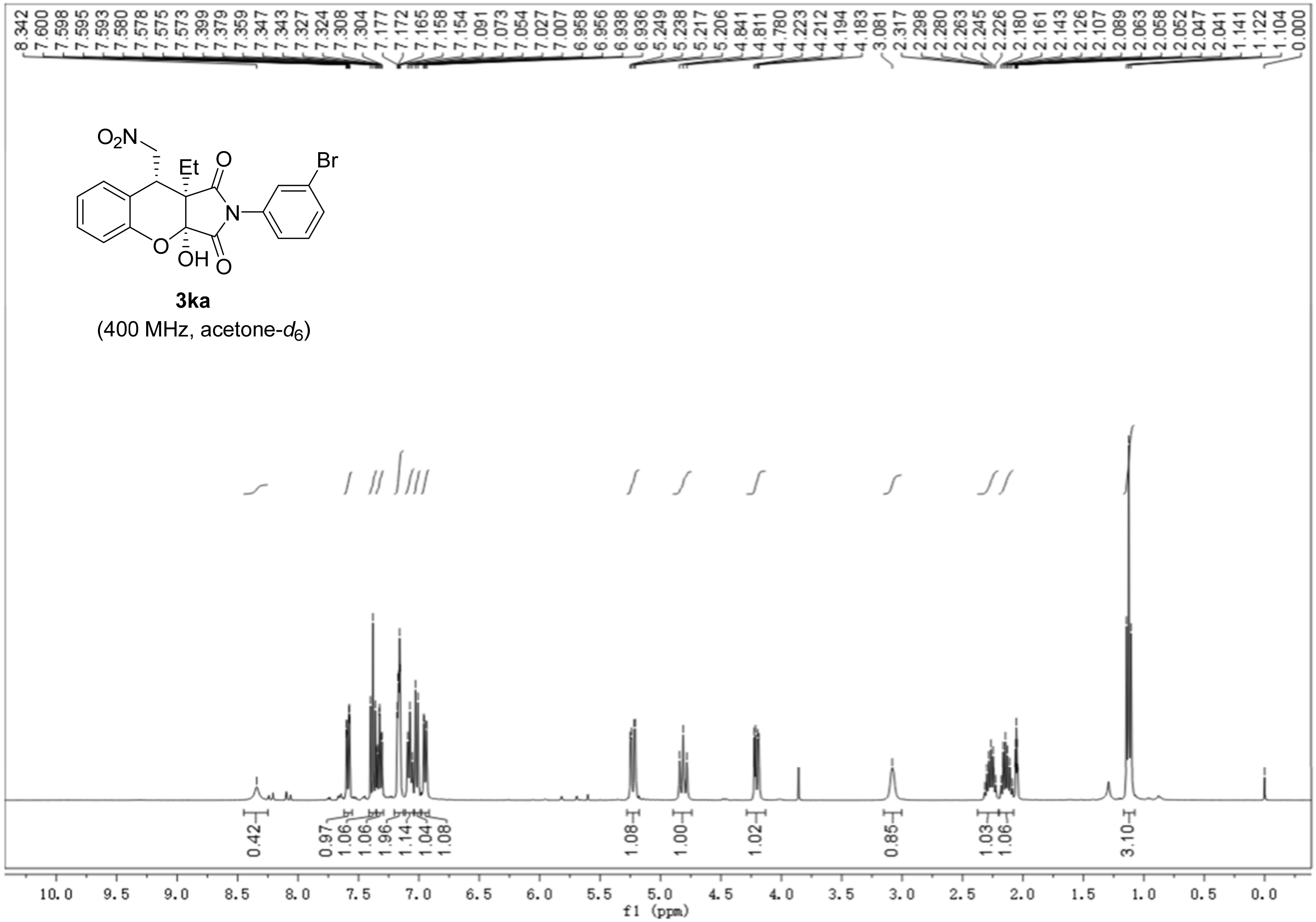

3ka
(400 MHz, acetone-d6)
f1 (ppm)

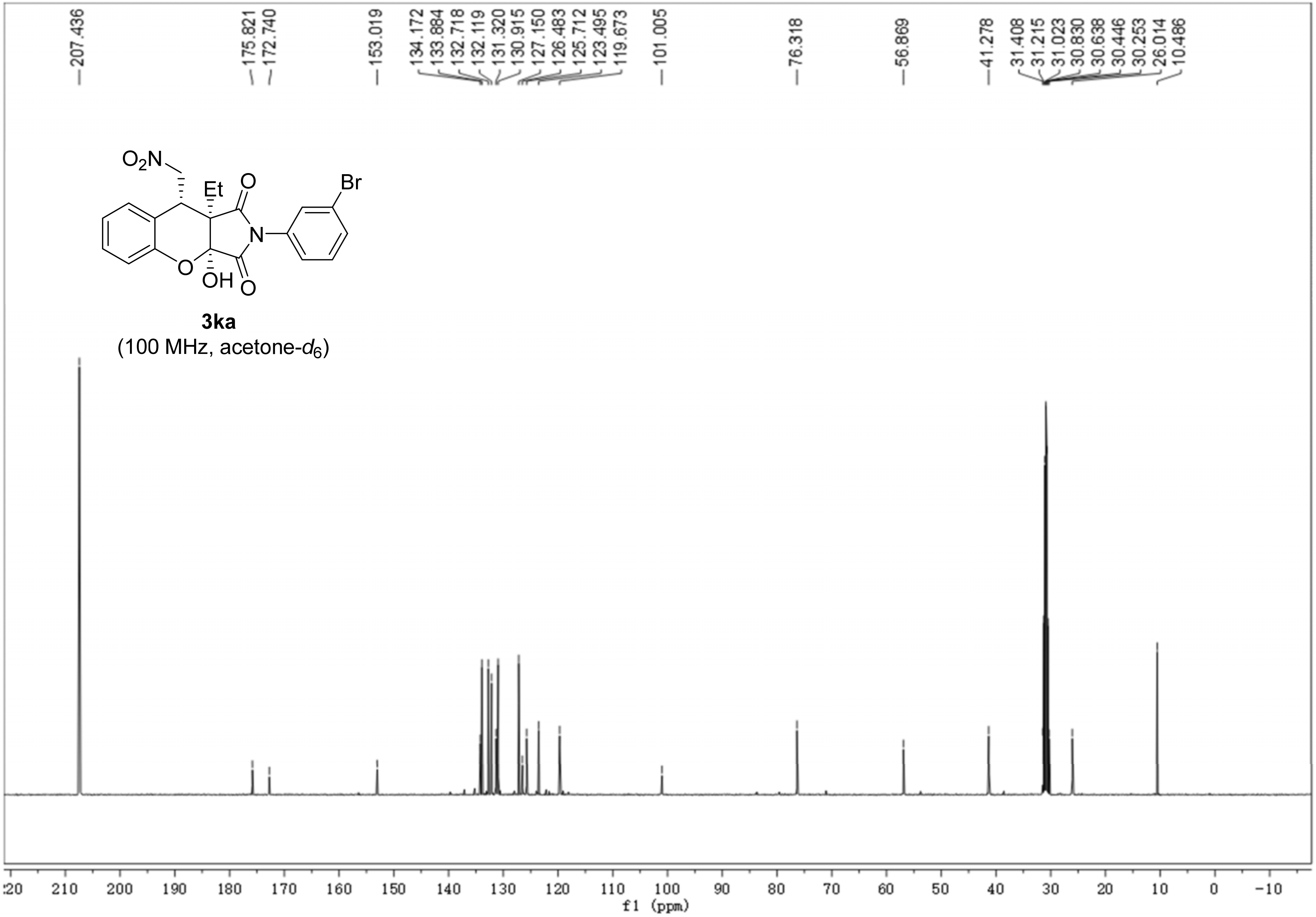
207.436
175.821
172.740
153.019
134.172
133.884
132.718
132.119
131.320
130.915
127.150
126.483
125.712
123.495
119.673
101.005
76.318
56.869
41.278
31.408
31.215
31.023
30.830
30.638
30.446
30.253
26.014
10.486
O2N
Et
O
Br
N
O
OH
O
3ka
(100 MHz, acetone-d6)
f1 (ppm)

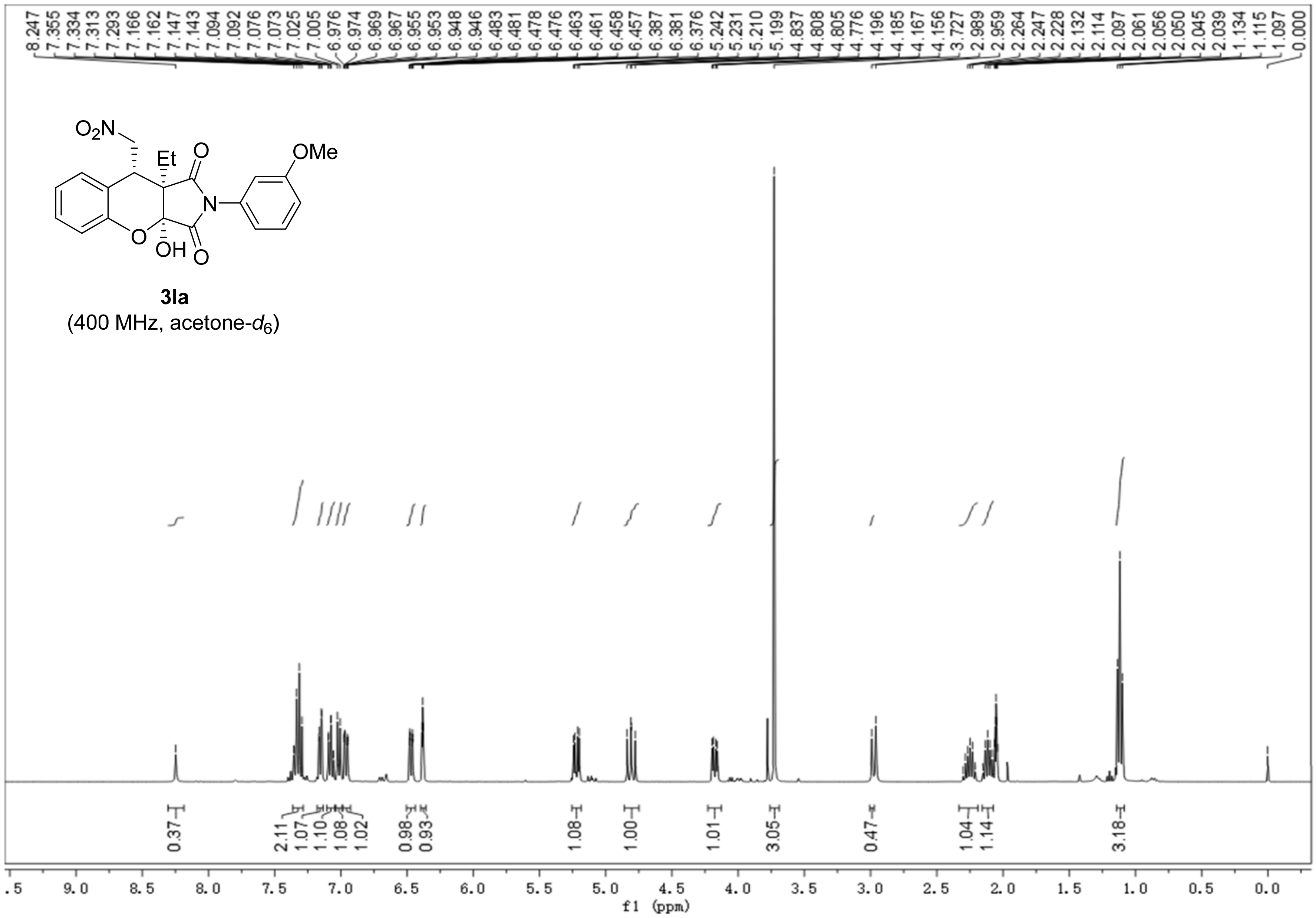

O_2N
Et
OMe
O
N
O
OH
O
3la
(400 MHz, acetone-d_6)
f1 (ppm)

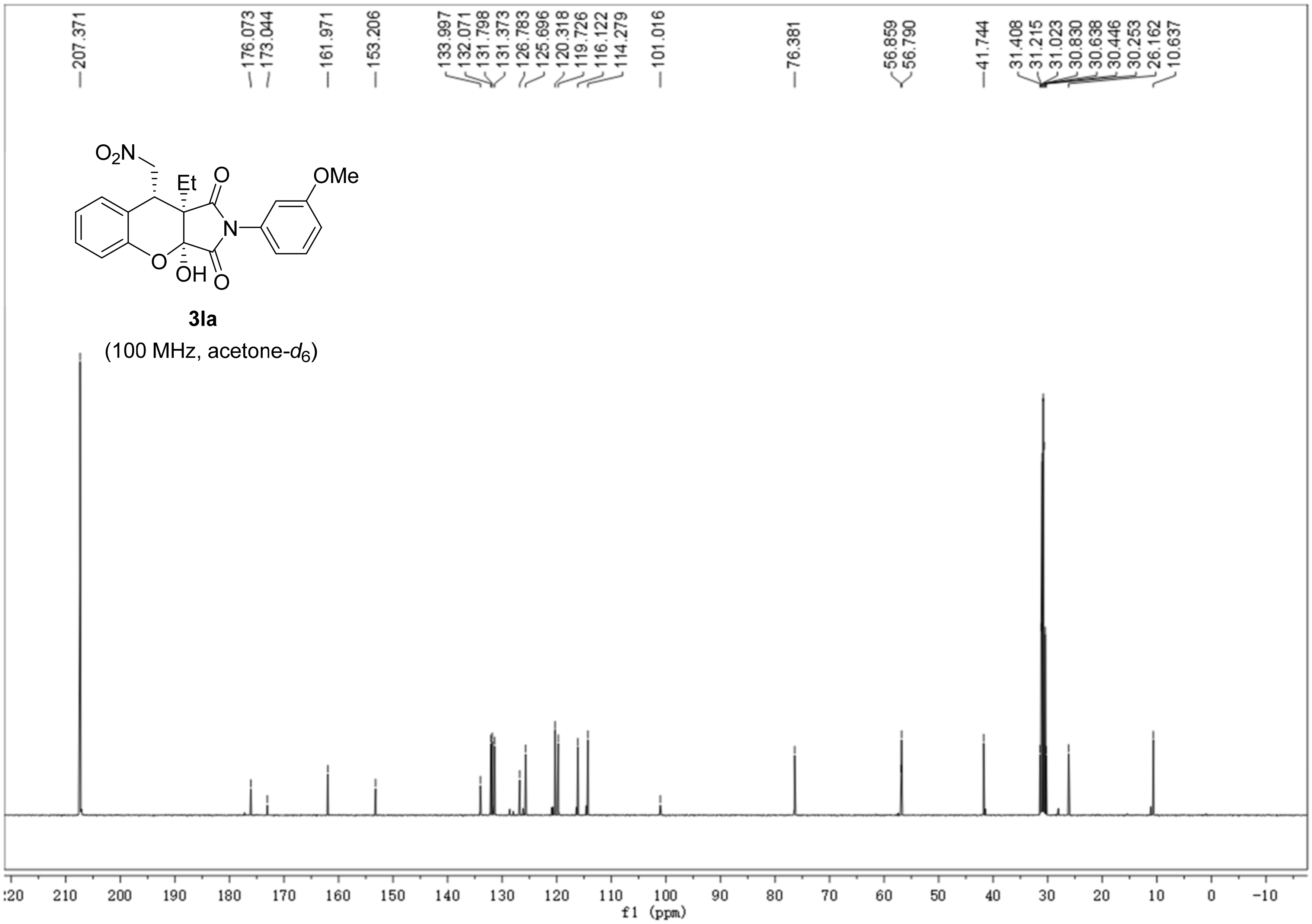
207.371
176.073
173.044
161.971
153.206
133.997
132.071
131.798
131.373
126.783
125.696
120.318
119.726
116.122
114.279
101.016
76.381
56.859
56.790
41.744
31.408
31.215
31.023
30.830
30.638
30.446
30.253
26.162
10.637
O_2N
Et
O
OMe
N
O
OH
O
3la
(100 MHz, acetone-d_6)
f1 (ppm)

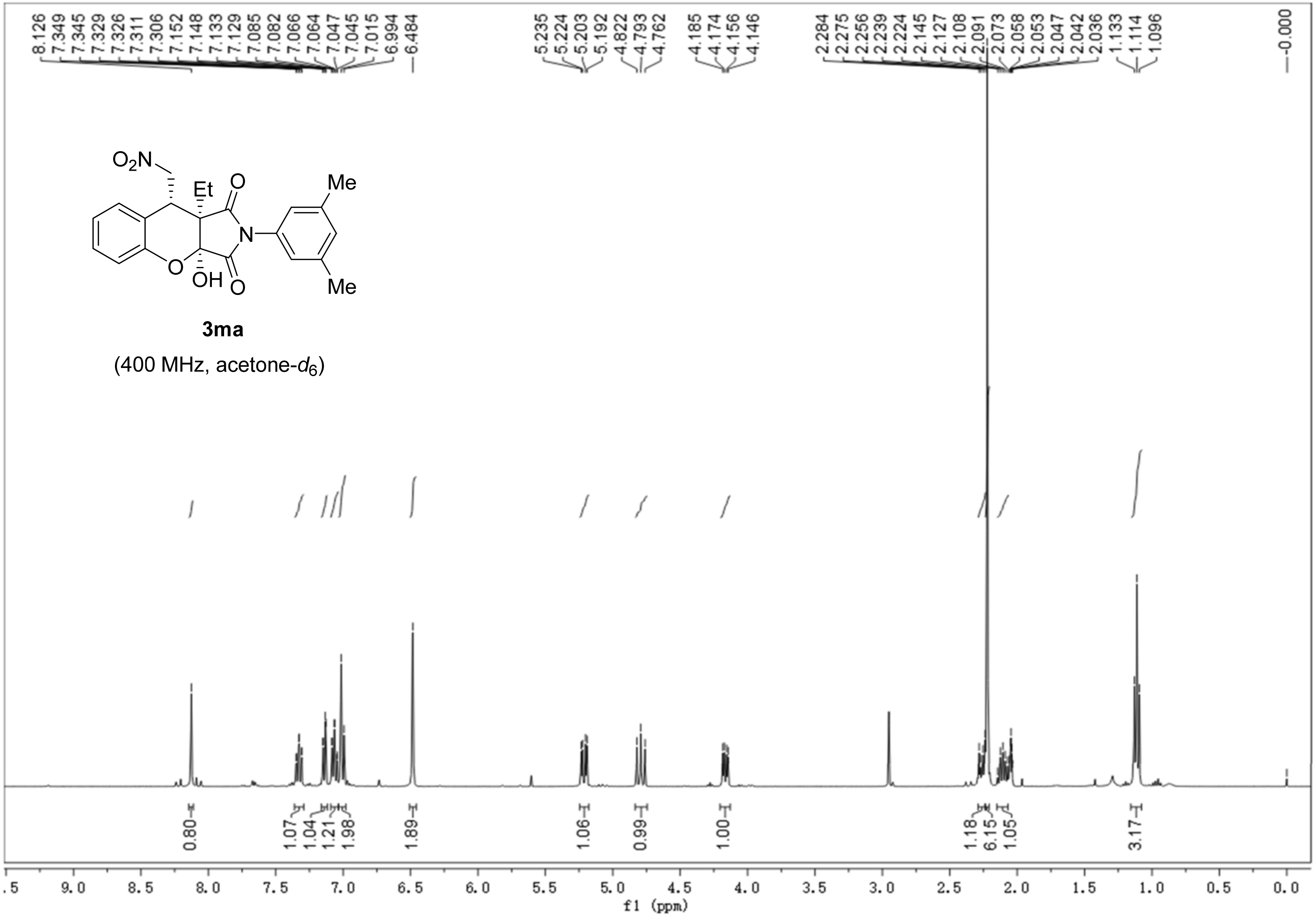

8.126
7.349
7.345
7.329
7.326
7.311
7.306
7.152
7.148
7.133
7.129
7.085
7.082
7.066
7.064
7.047
7.045
7.015
6.994
6.484
5.235
5.224
5.203
5.192
4.822
4.793
4.762
4.185
4.174
4.156
4.146
2.284
2.275
2.256
2.239
2.224
2.145
2.127
2.108
2.091
2.073
2.058
2.053
2.047
2.042
2.036
1.133
1.114
1.096
-0.000
O2N
Et
O
N
Me
O
OH
O
Me
3ma
(400 MHz, acetone-d6)
0.80
1.07
1.04
1.21
1.98
1.89
1.06
0.99
1.00
1.18
6.15
1.05
3.17
f1 (ppm)

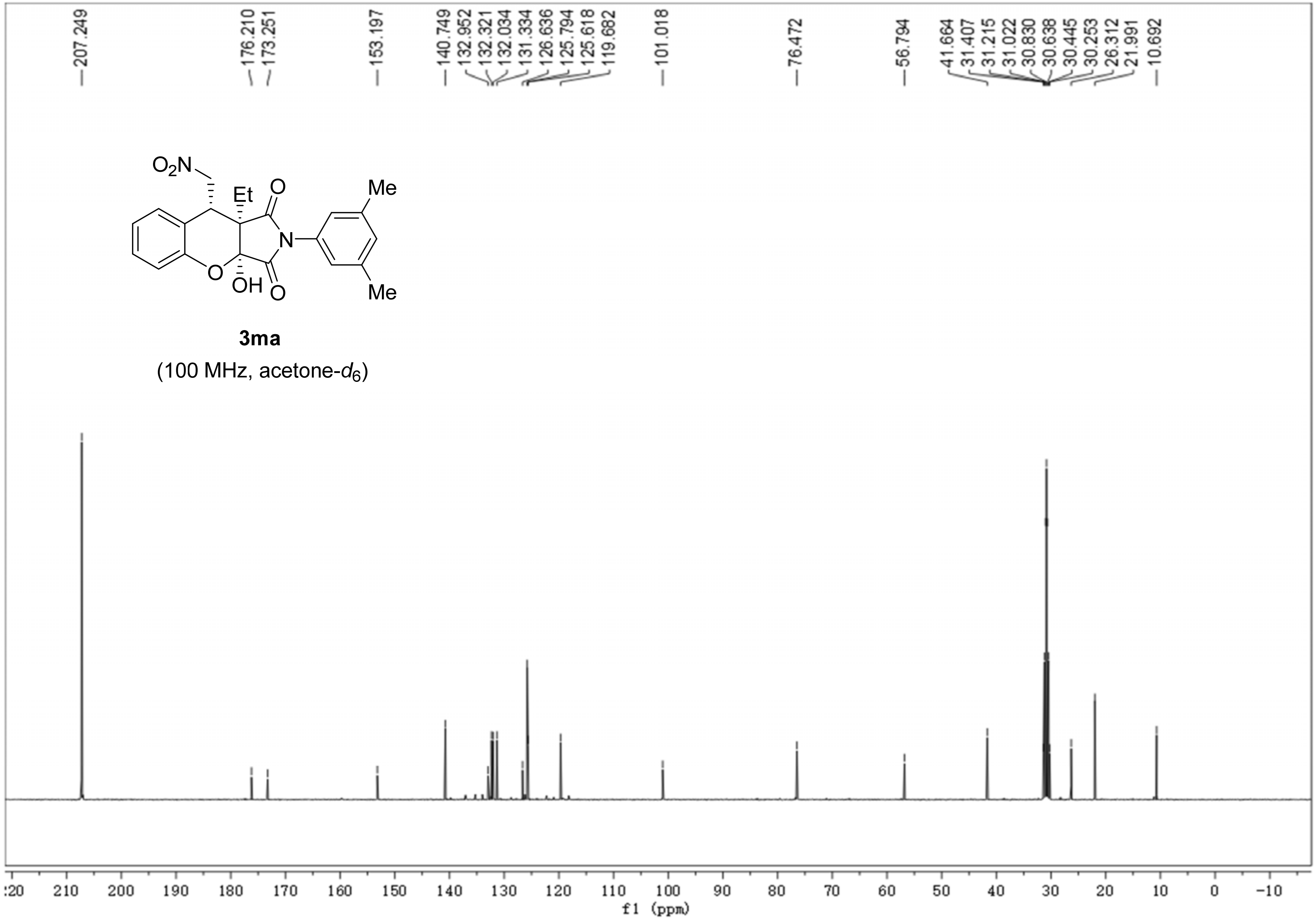
207.249
176.210
173.251
153.197
140.749
132.952
132.321
132.034
131.334
126.636
125.794
125.618
119.682
101.018
76.472
56.794
41.664
31.407
31.215
31.022
30.830
30.638
30.445
30.253
26.312
21.991
10.692
O2N
Et
O
N
Me
O
OH
O
Me
3ma
(100 MHz, acetone-d6)
f1 (ppm)

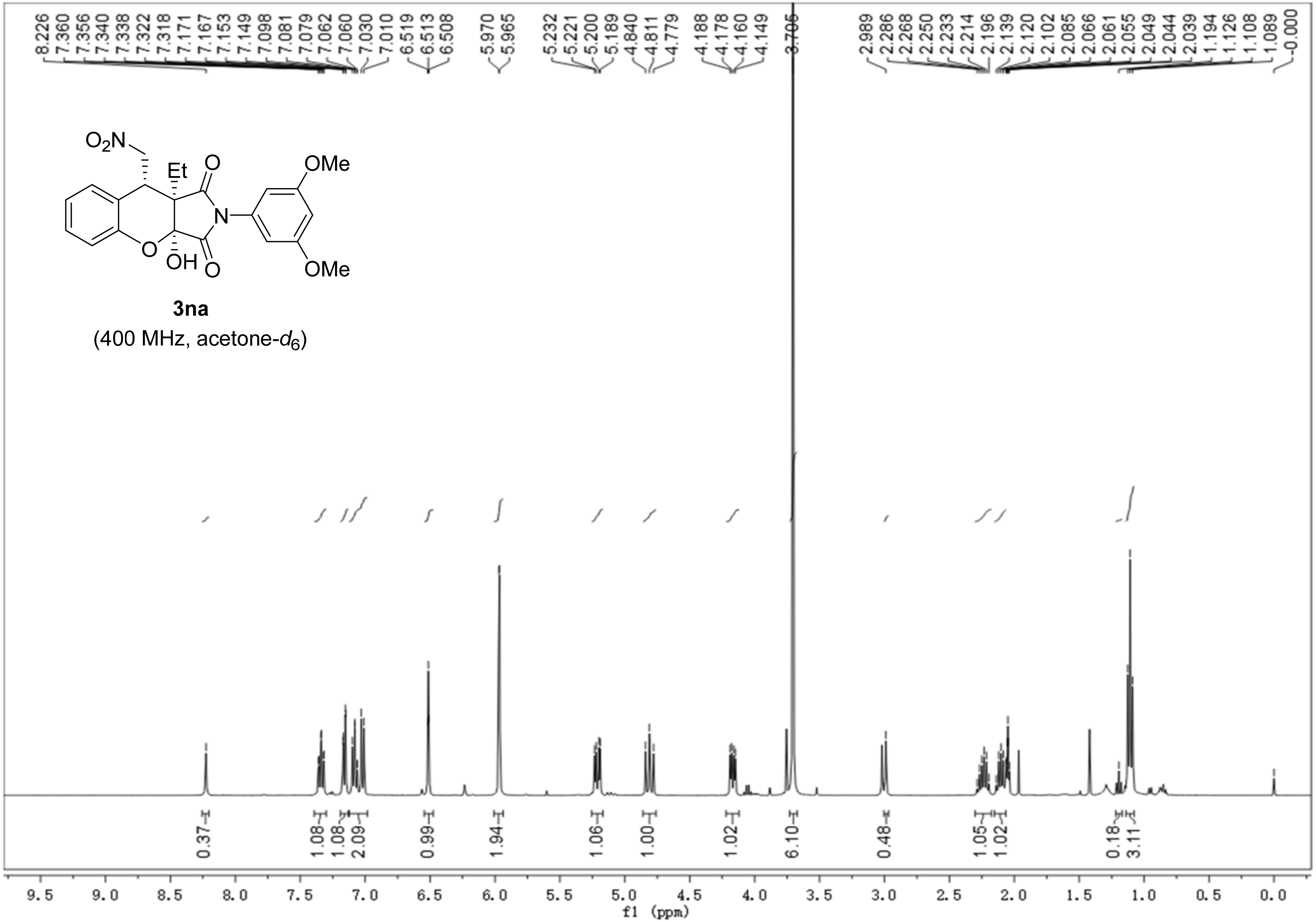
3na
(400 MHz, acetone-d6)
f1 (ppm)

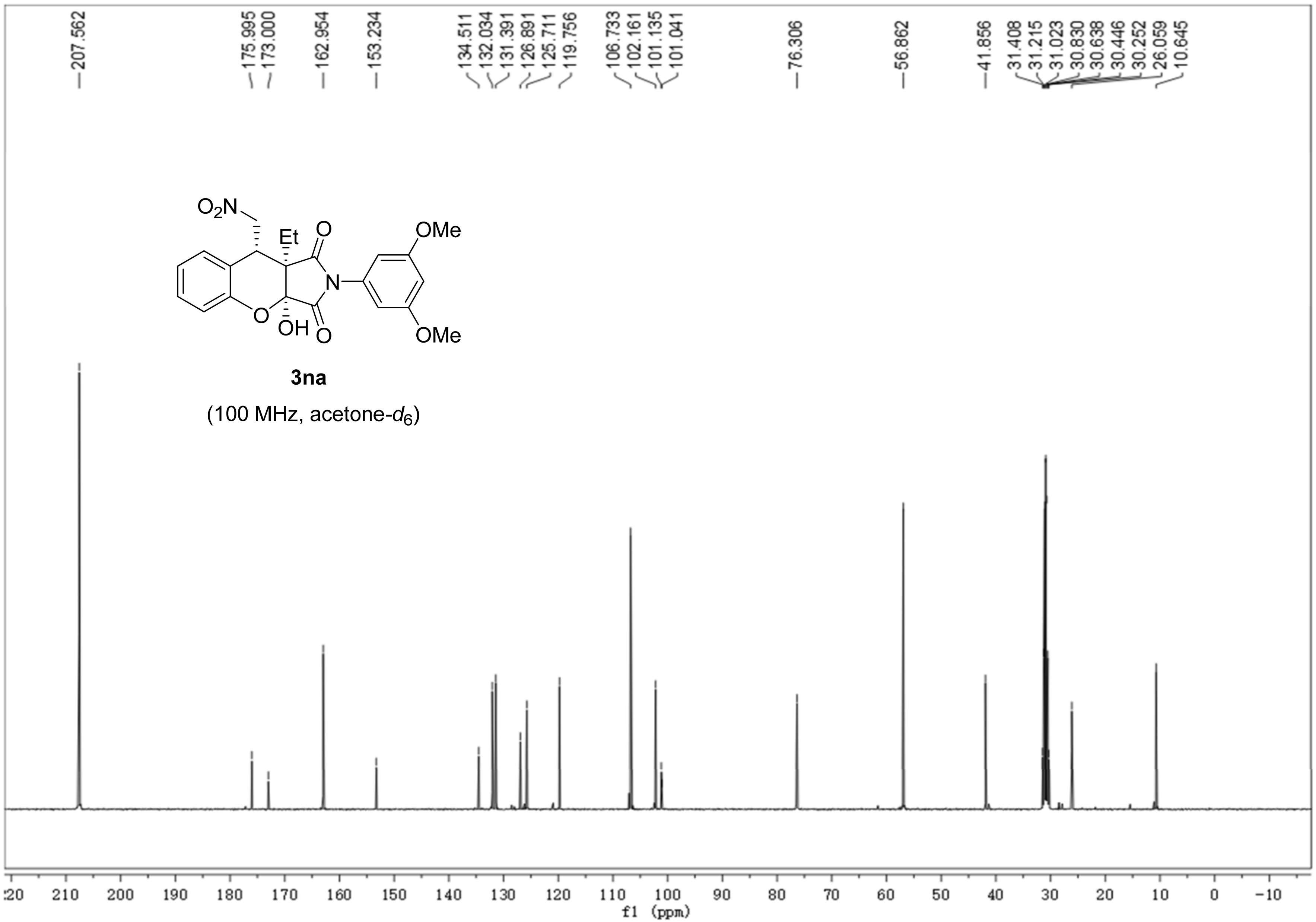

207.562
175.995
173.000
162.954
153.234
134.511
132.034
131.391
126.891
125.711
119.756
106.733
102.161
101.135
101.041
76.306
56.862
41.856
31.408
31.215
31.023
30.830
30.638
30.446
30.252
26.059
10.645
3na
(100 MHz, acetone-d_6)
f1 (ppm)

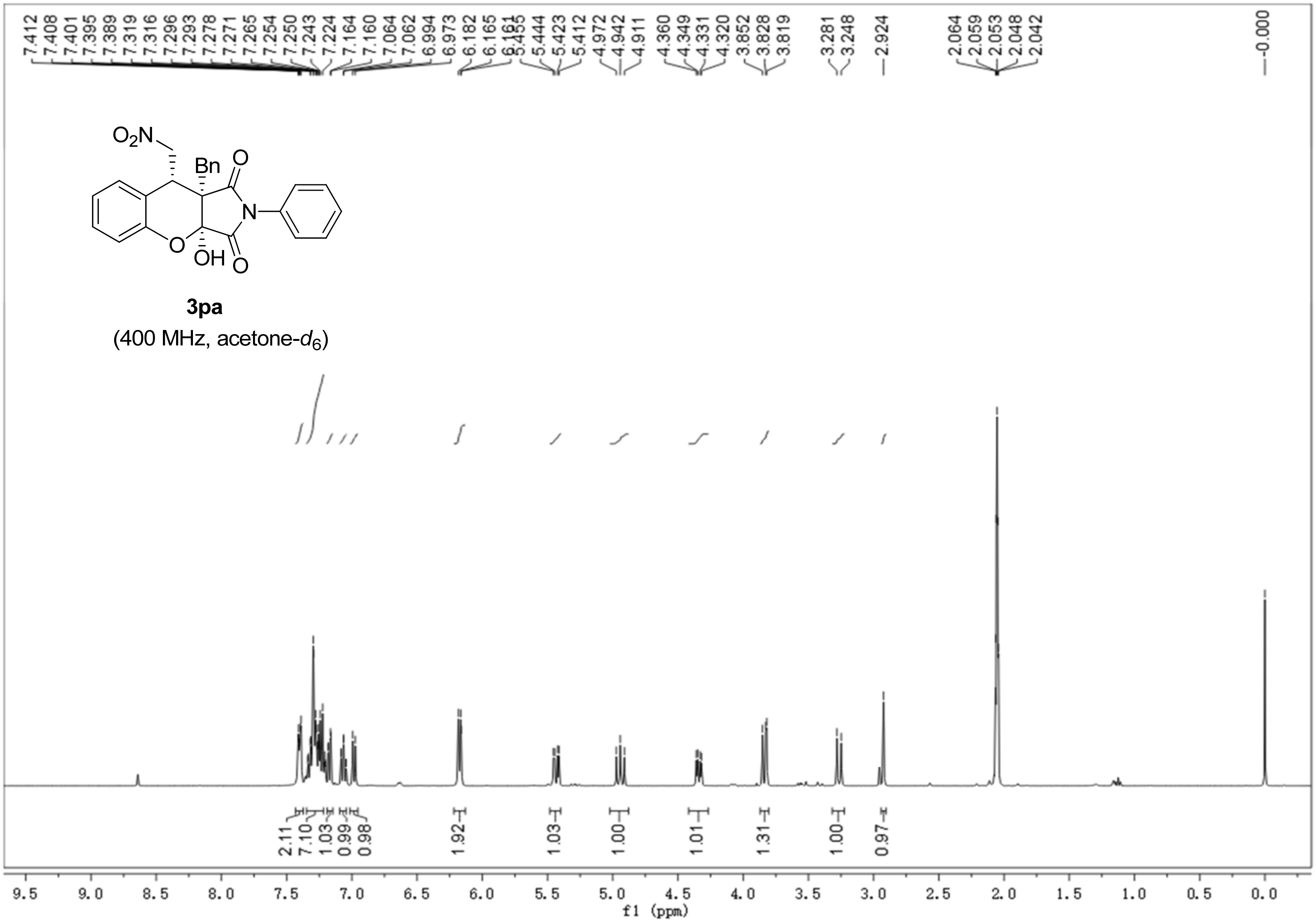
7.412 7.408 7.401 7.395 7.389 7.319 7.316 7.296 7.293 7.278 7.271 7.265 7.254 7.250 7.243 7.224 7.164 7.160 7.064 7.062 6.994 6.973 6.182 6.165 6.161 5.455 5.444 5.423 5.412 4.972 4.942 4.911 4.360 4.349 4.331 4.320 3.852 3.828 3.819 3.281 3.248 2.924 2.064 2.059 2.053 2.048 2.042 -0.000
O_2N
Bn
O
N
O
OH
O
3pa
(400 MHz, acetone-d_6)
2.11 7.10 1.03 0.99 0.98 1.92 1.03 1.00 1.01 1.31 1.00 0.97
9.5 9.0 8.5 8.0 7.5 7.0 6.5 6.0 5.5 5.0 4.5 4.0 3.5 3.0 2.5 2.0 1.5 1.0 0.5 0.0
f1 (ppm)

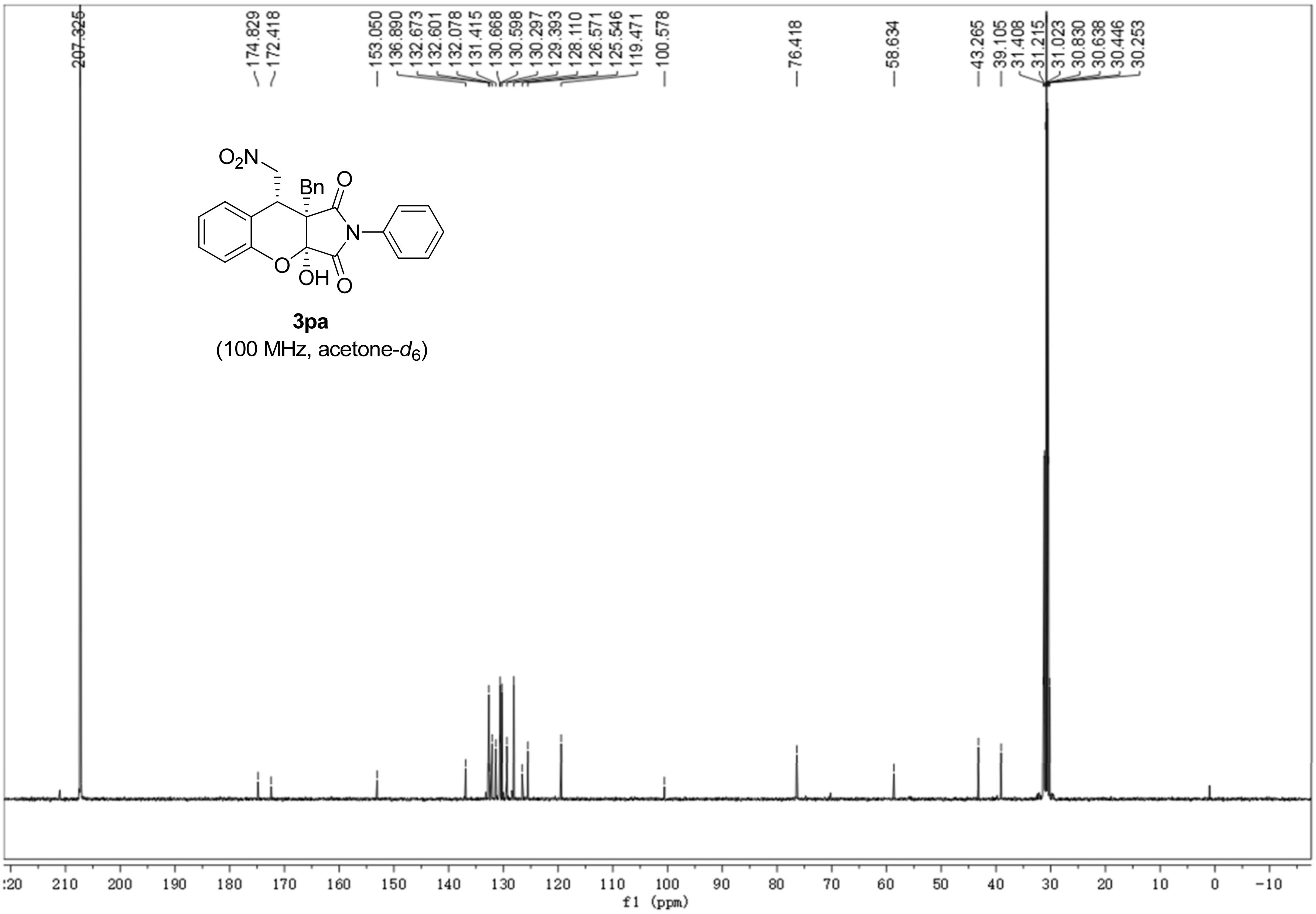
207.325
174.829
172.418
153.050
136.890
132.673
132.601
132.078
131.415
130.668
130.598
130.297
129.393
128.110
126.571
125.546
119.471
100.578
76.418
58.634
43.265
39.105
31.408
31.215
31.023
30.830
30.638
30.446
30.253
O2N
Bn
O
N
O
OH
O
3pa
(100 MHz, acetone-d6)
f1 (ppm)

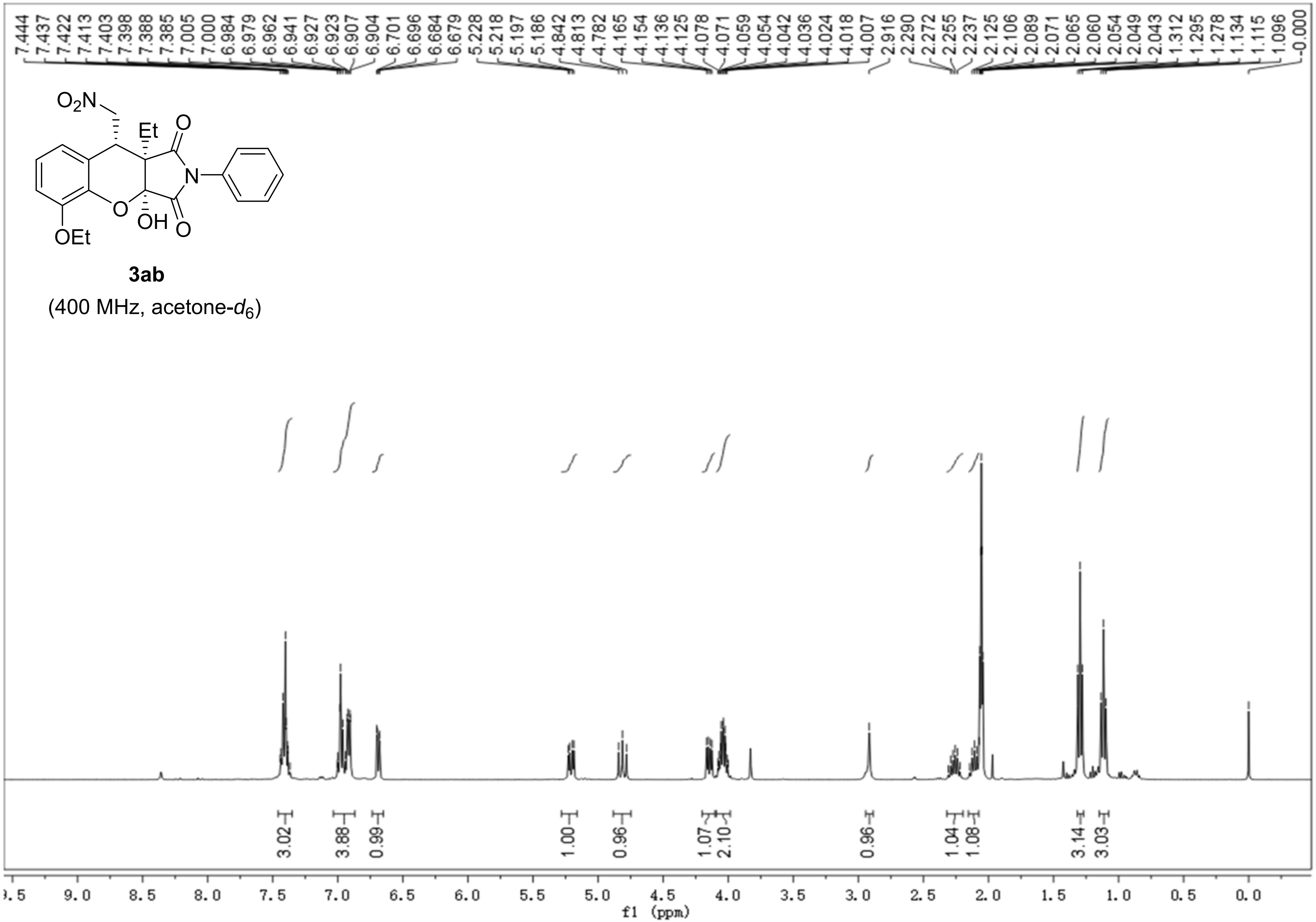
7.444
7.437
7.422
7.413
7.403
7.398
7.388
7.385
7.005
7.000
6.984
6.979
6.962
6.941
6.927
6.923
6.907
6.904
6.701
6.696
6.684
6.679
5.228
5.218
5.197
5.186
4.842
4.813
4.782
4.165
4.154
4.136
4.125
4.078
4.071
4.059
4.054
4.042
4.036
4.024
4.018
4.007
2.916
2.290
2.272
2.255
2.237
2.125
2.106
2.089
2.071
2.065
2.060
2.054
2.049
2.043
1.312
1.295
1.278
1.134
1.115
1.096
-0.000
O2N
Et
O
N
O
OH
O
OEt
3ab
(400 MHz, acetone-d6)
3.02
3.88
0.99
1.00
0.96
1.07
2.10
0.96
1.04
1.08
3.14
3.03
9.5
9.0
8.5
8.0
7.5
7.0
6.5
6.0
5.5
5.0
4.5
4.0
3.5
3.0
2.5
2.0
1.5
1.0
0.5
0.0
f1 (ppm)

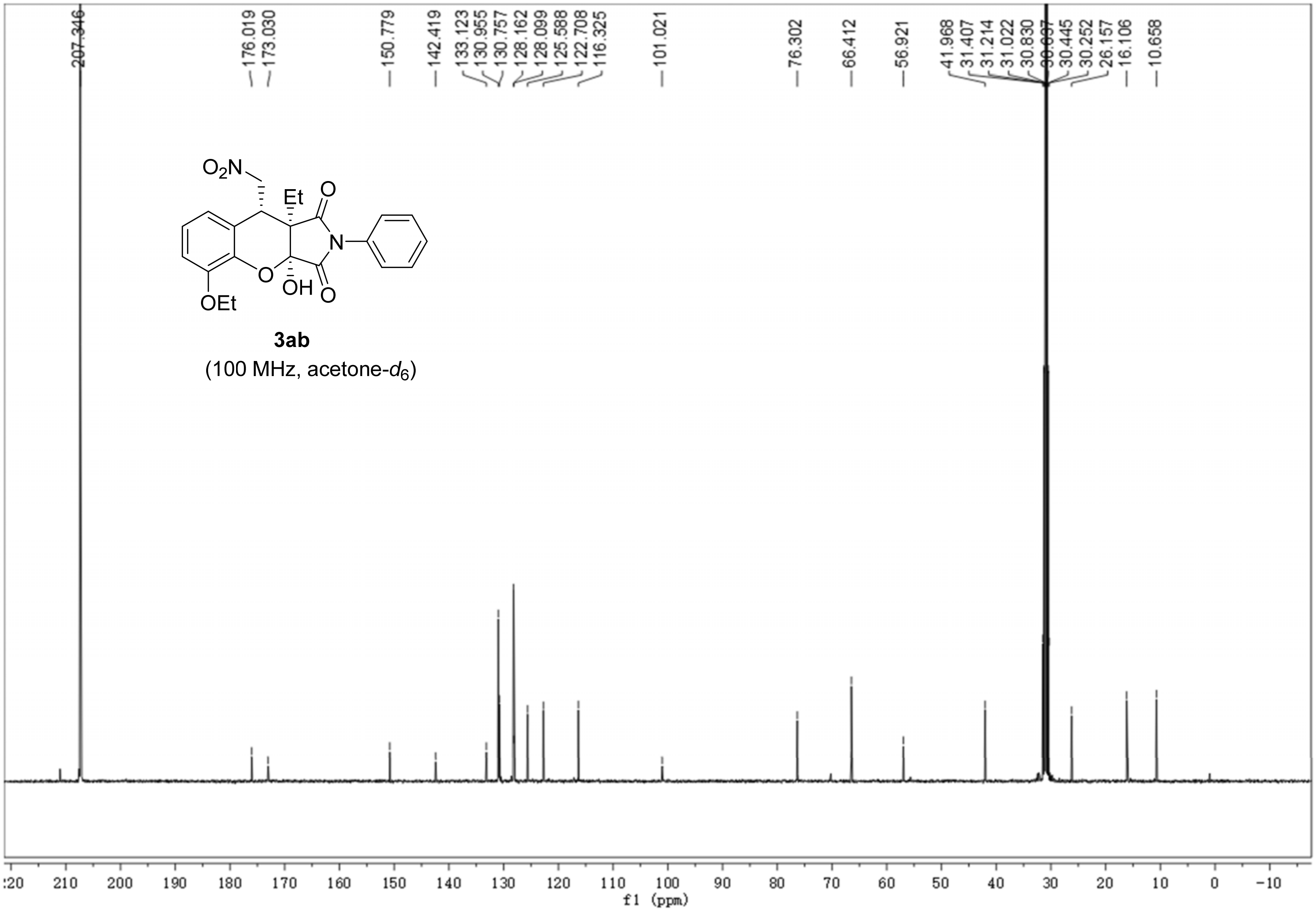
207.346
176.019
173.030
150.779
142.419
133.123
130.955
130.757
128.162
128.099
125.588
122.708
116.325
101.021
76.302
66.412
56.921
41.968
31.407
31.214
31.022
30.830
30.445
30.252
26.157
16.106
10.658
O_2N
Et
O
N
O
OH
O
OEt
3ab
(100 MHz, acetone-d_6)
f1 (ppm)

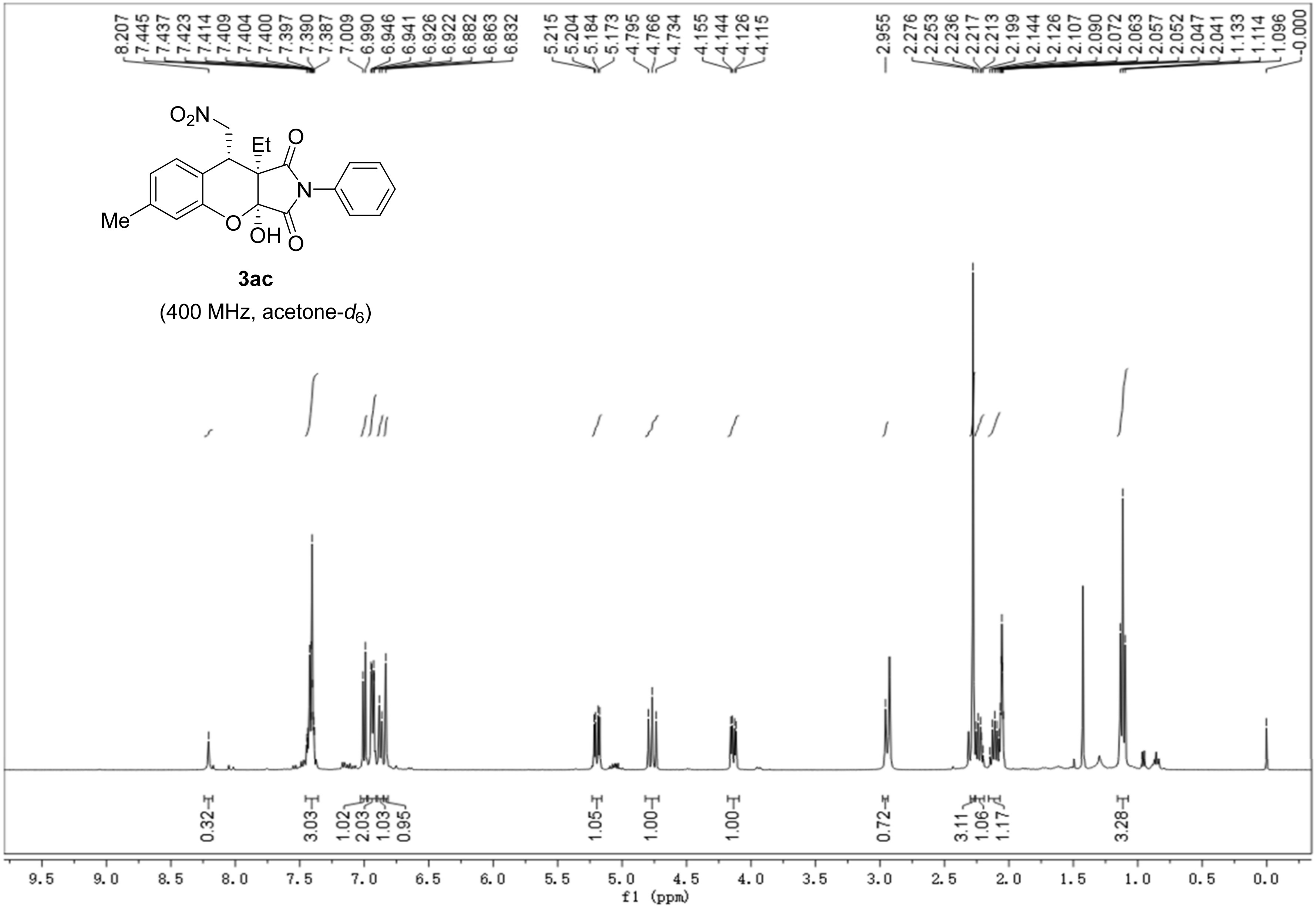

3ac

(400 MHz, acetone-d_6)

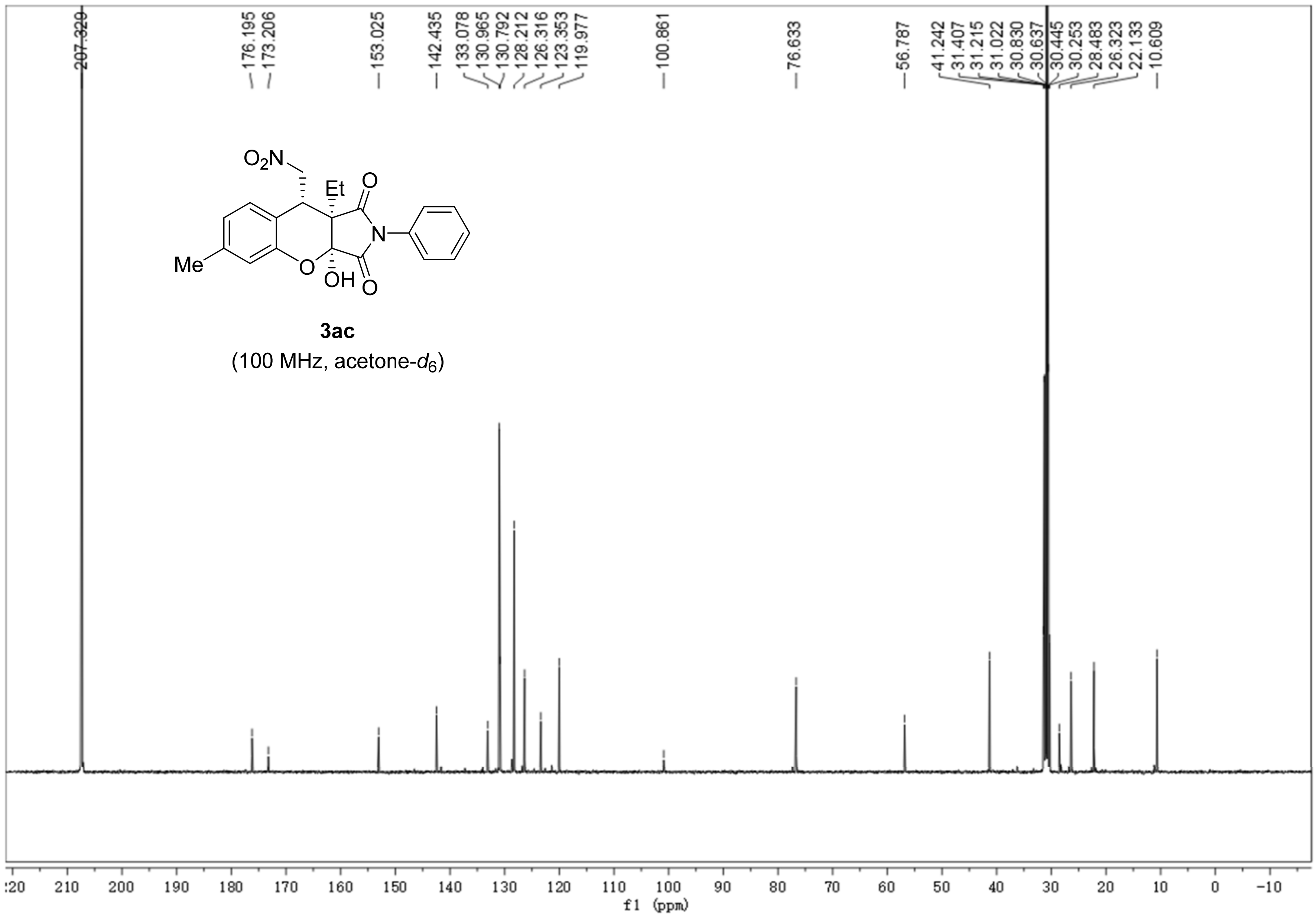

207.329
176.195
173.206
153.025
142.435
133.078
130.965
130.792
128.212
126.316
123.353
119.977
100.861
76.633
56.787
41.242
31.407
31.215
31.022
30.830
30.637
30.445
30.253
28.483
26.323
22.133
10.609
O_2N
Et
O
N
Me
O
OH
O
3ac
(100 MHz, acetone-d_6)
f1 (ppm)

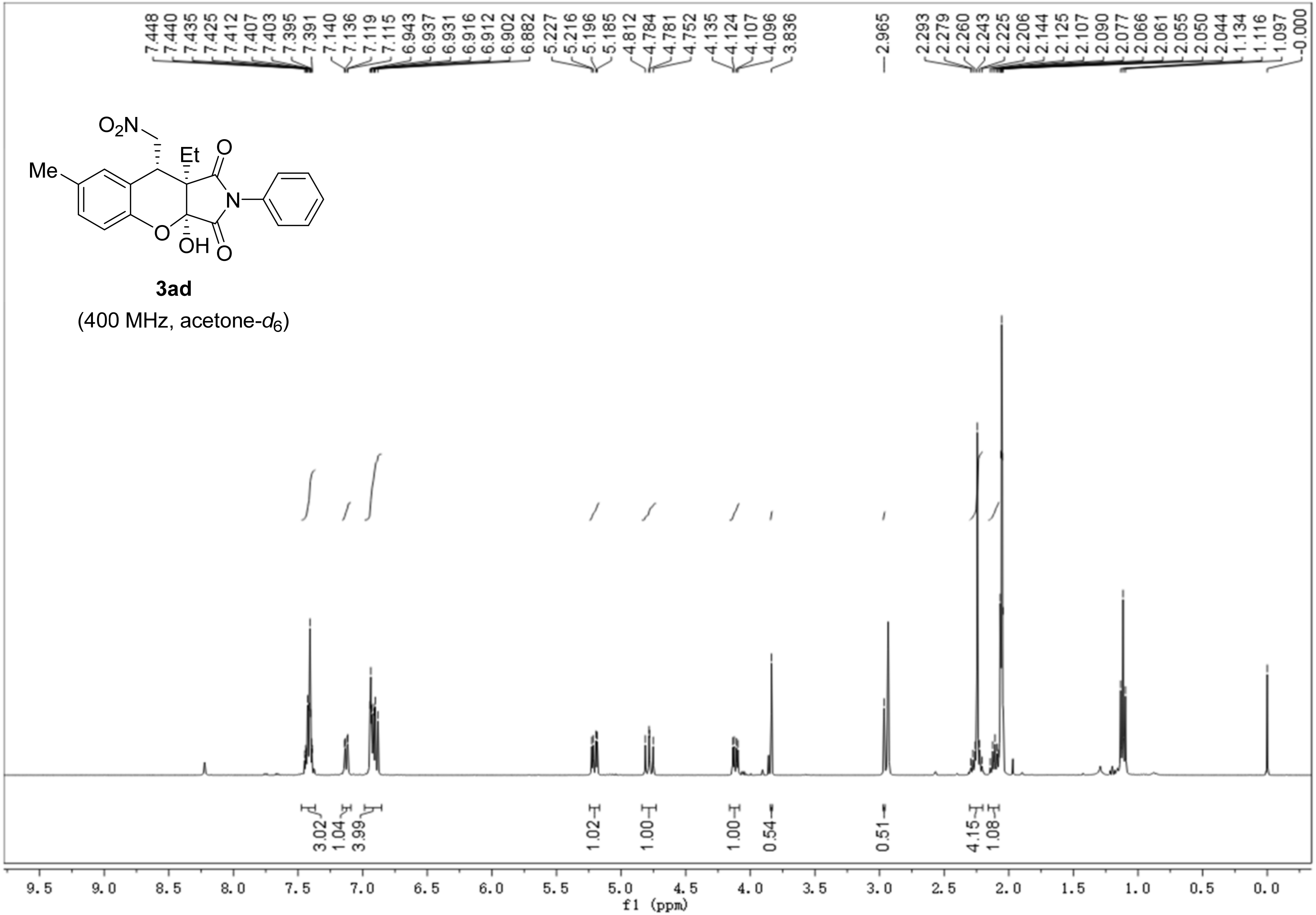

3ad

(400 MHz, acetone-d_6)

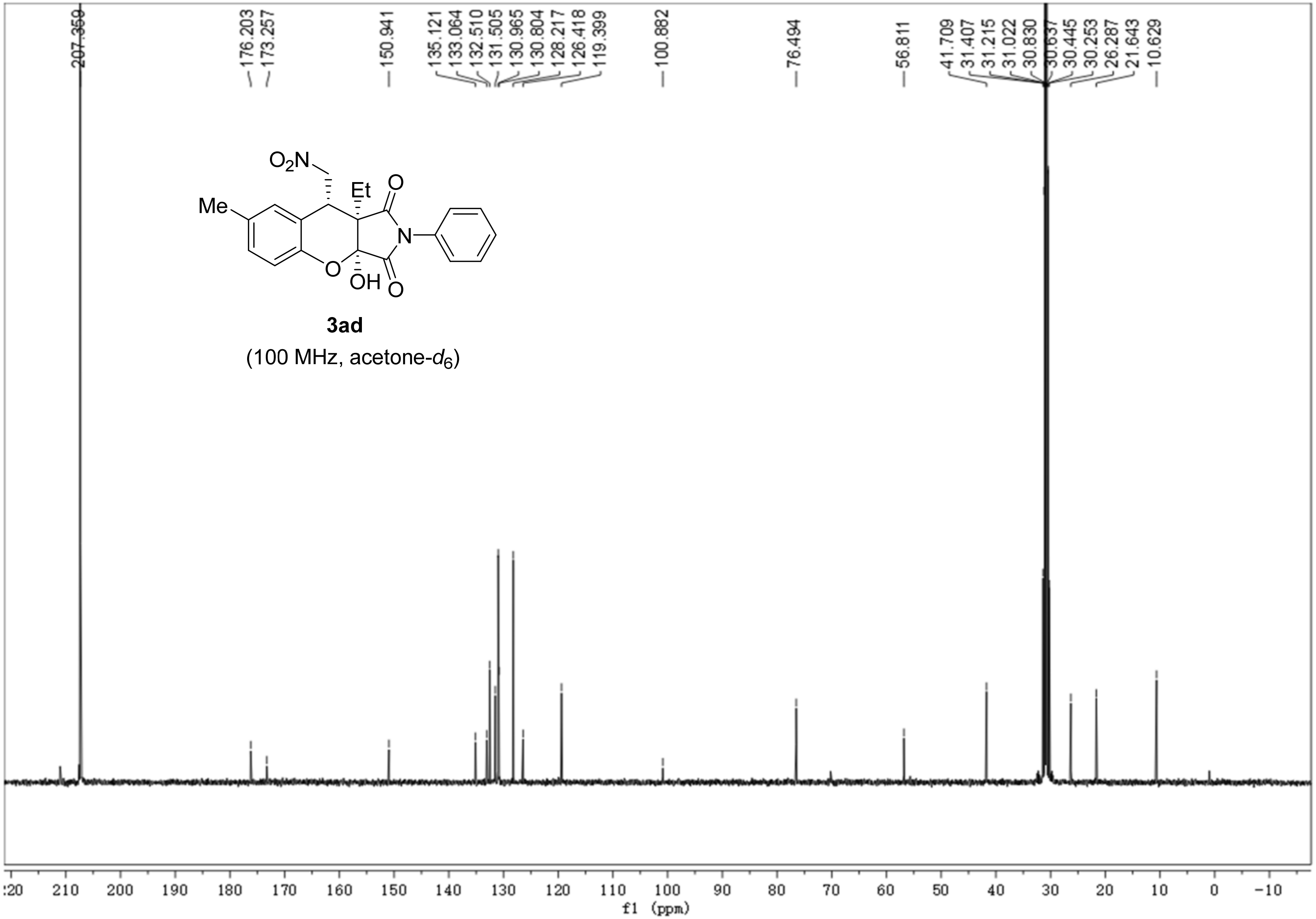

207.359
176.203
173.257
150.941
135.121
133.064
132.510
131.505
130.965
130.804
128.217
126.418
119.399
100.882
76.494
56.811
41.709
31.407
31.215
31.022
30.830
30.637
30.445
30.253
26.287
21.643
10.629
O_2N
Et
Me
O
N
OH
3ad
(100 MHz, acetone-d_6)
f1 (ppm)

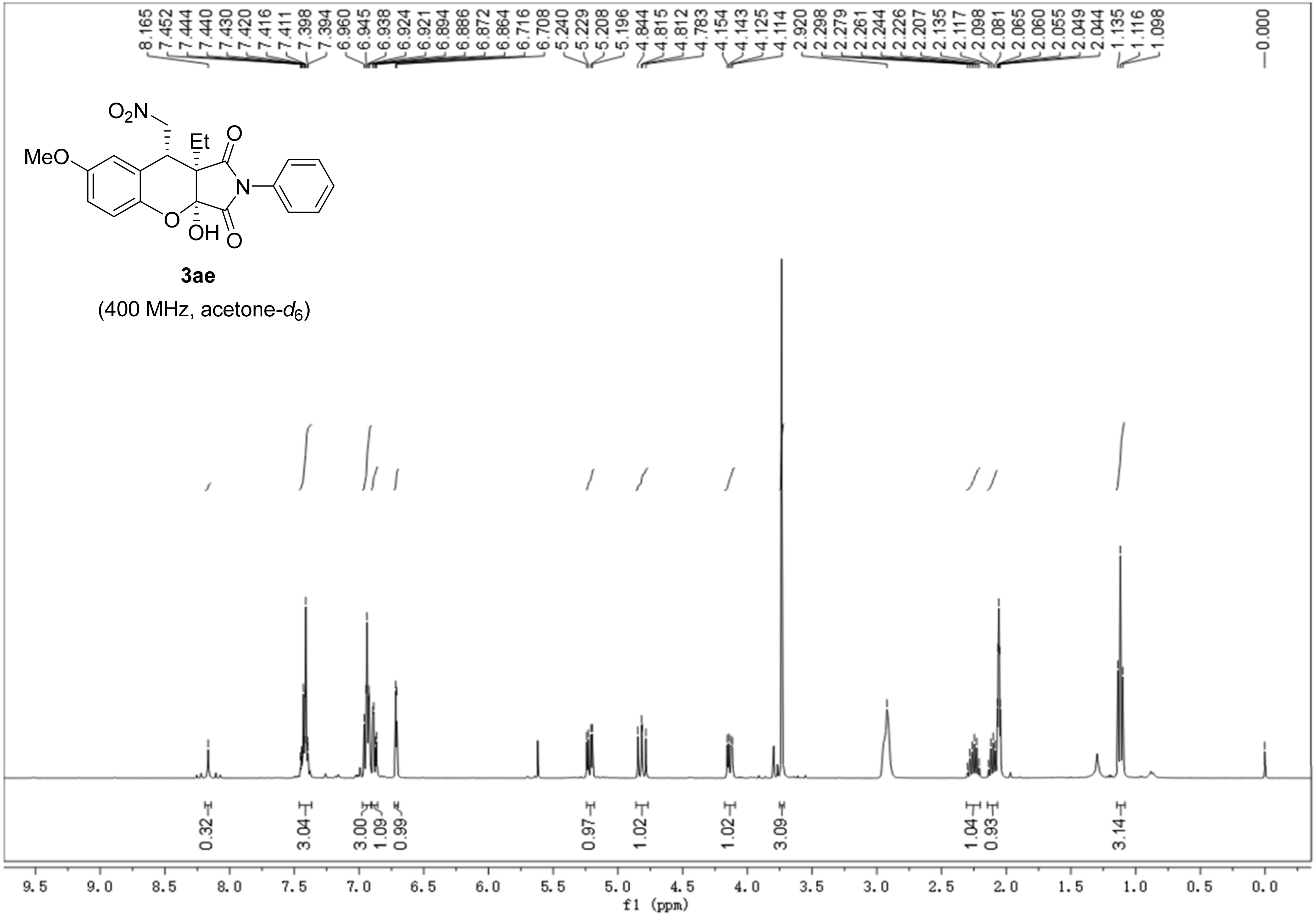
8.165 7.452 7.444 7.440 7.430 7.420 7.416 7.411 7.398 7.394 6.960 6.945 6.938 6.924 6.921 6.894 6.886 6.872 6.864 6.716 6.708 5.240 5.229 5.208 5.196 4.844 4.815 4.812 4.783 4.154 4.143 4.125 4.114 2.920 2.298 2.279 2.261 2.244 2.226 2.207 2.135 2.117 2.098 2.081 2.065 2.060 2.055 2.049 2.044 1.135 1.116 1.098 -0.000
O_2N
Et
MeO
O
N
OH
3ae
(400 MHz, acetone-d_6)
0.32 3.04 3.00 1.09 0.99 0.97 1.02 1.02 3.09 1.04 0.93 3.14
9.5 9.0 8.5 8.0 7.5 7.0 6.5 6.0 5.5 5.0 4.5 4.0 3.5 3.0 2.5 2.0 1.5 1.0 0.5 0.0
f1 (ppm)

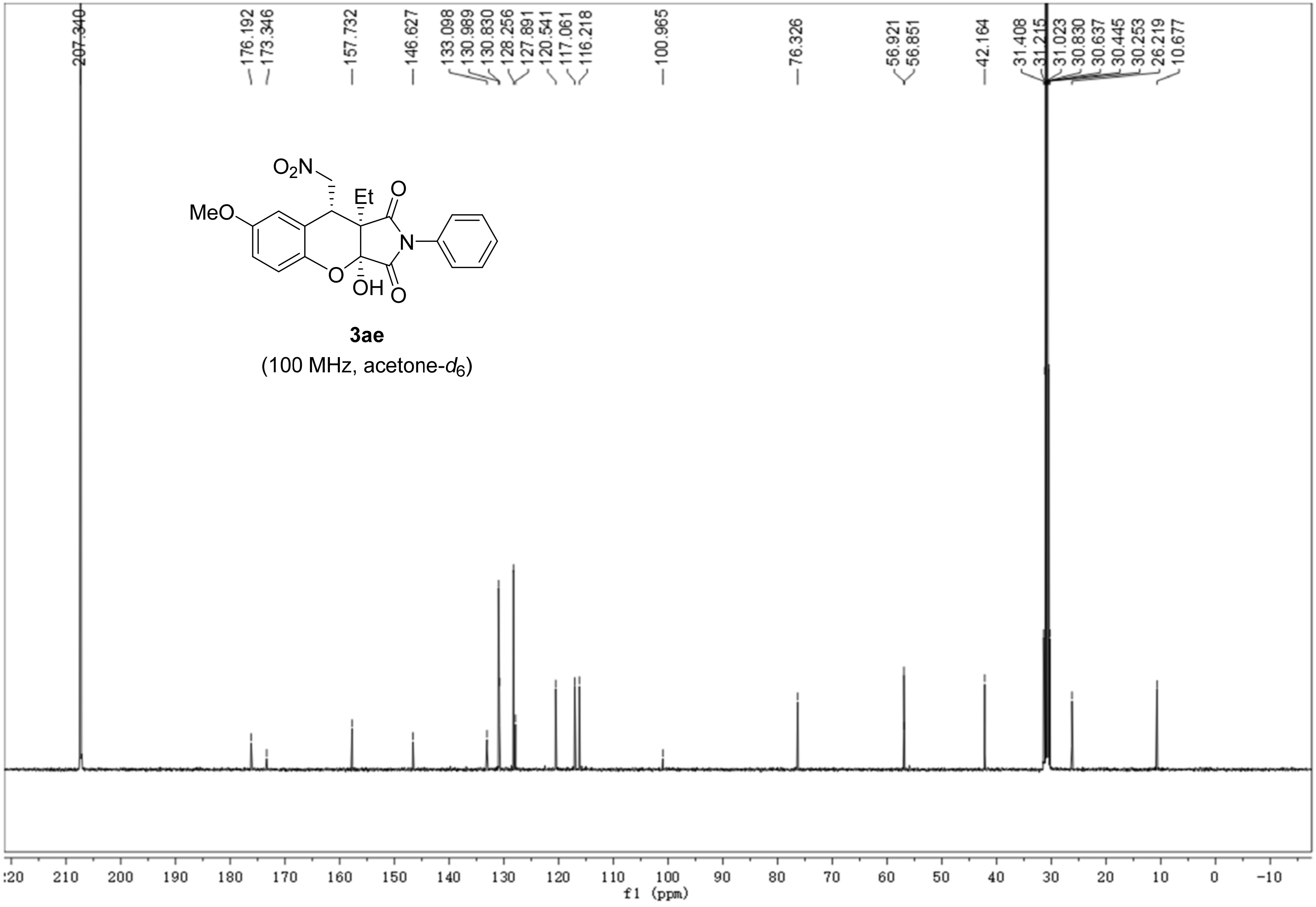
207.340
176.192
173.346
157.732
146.627
133.098
130.989
130.830
128.256
127.891
120.541
117.061
116.218
100.965
76.326
56.921
56.851
42.164
31.408
31.215
31.023
30.830
30.637
30.445
30.253
26.219
10.677
O_2N
Et
MeO
O
N
OH
3ae
(100 MHz, acetone-d_6)
f1 (ppm)

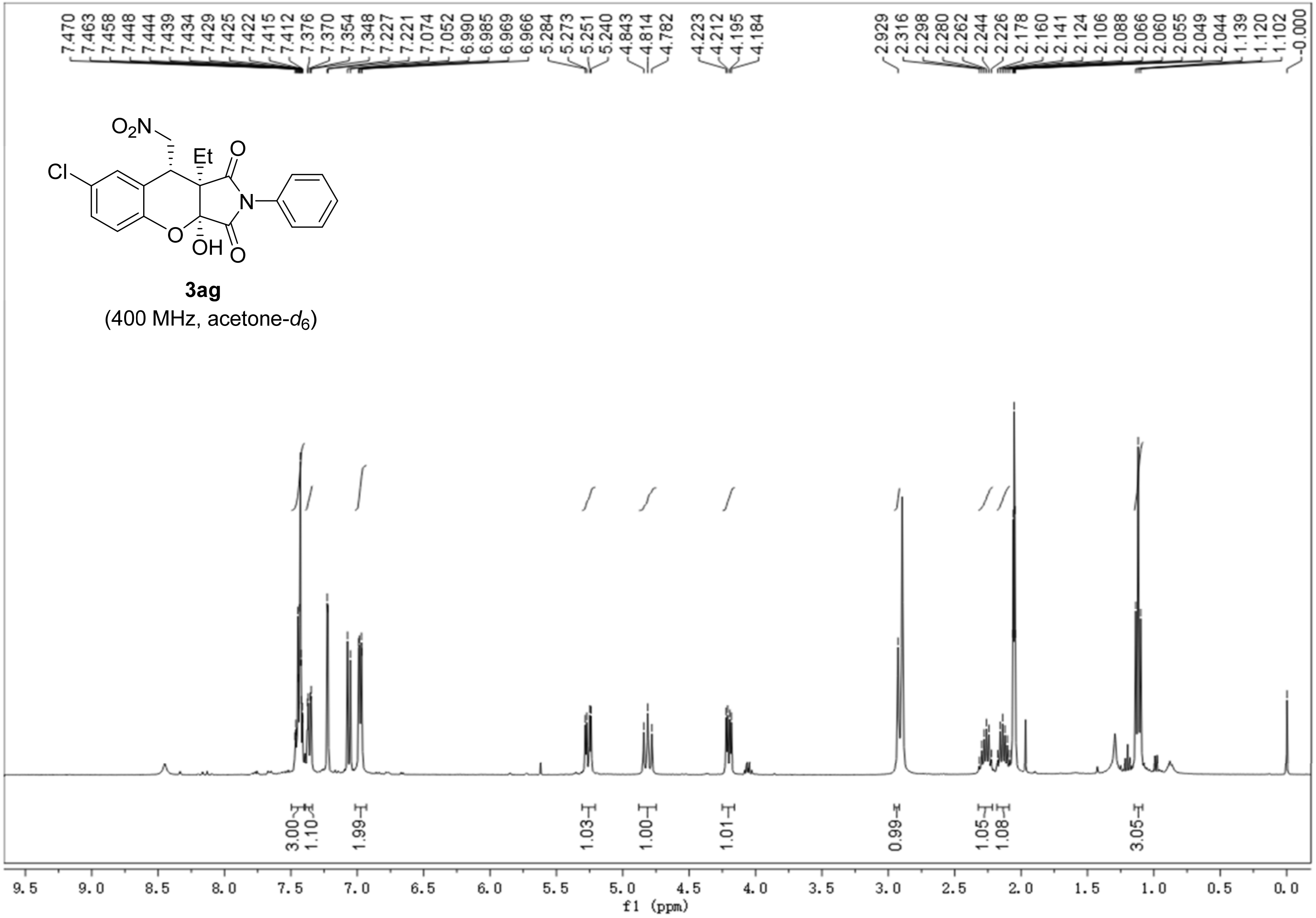
O2N
Et
Cl
N
O
OH
O
O
3ag
(400 MHz, acetone-d_6)
f1 (ppm)

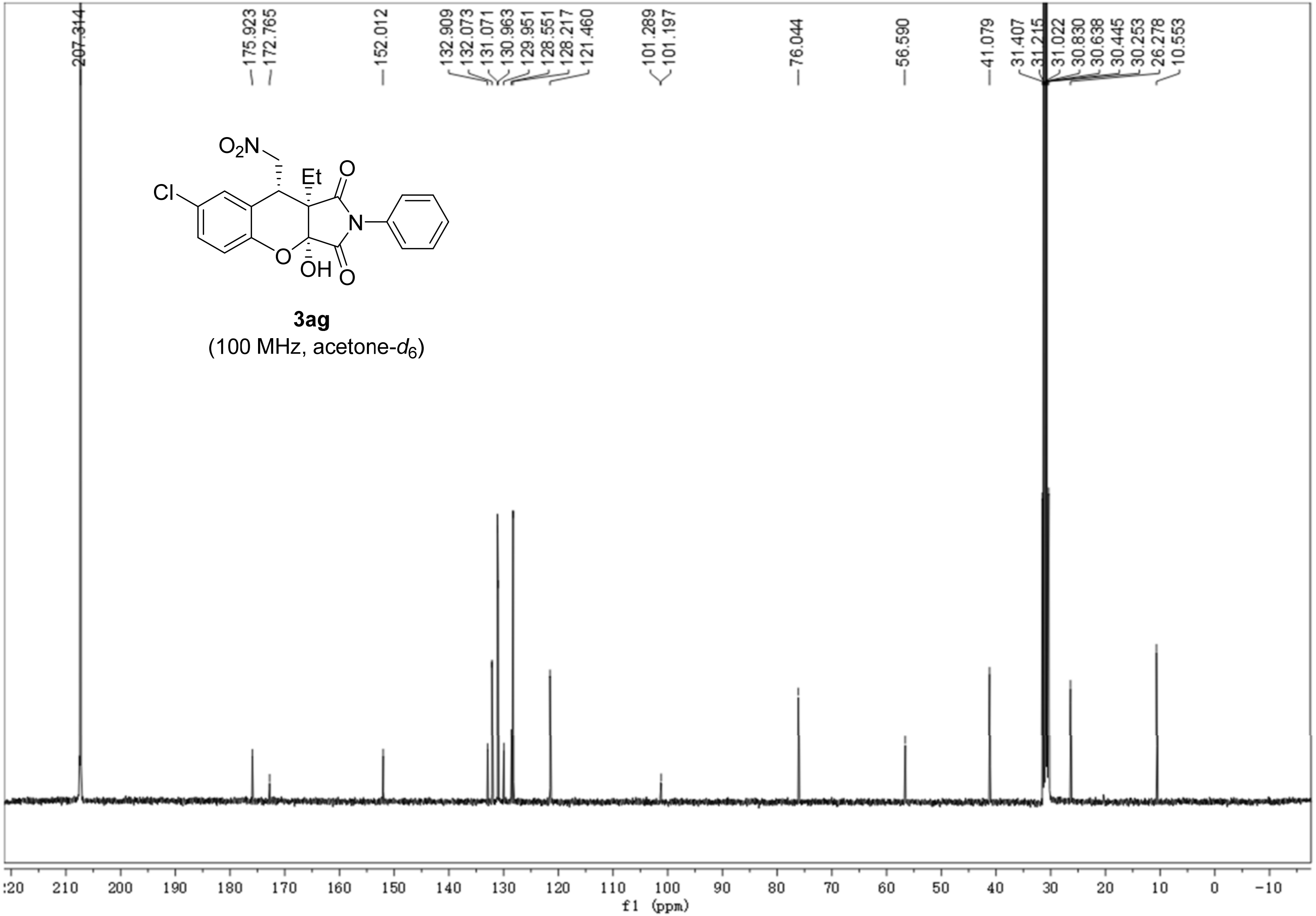

3ag
(100 MHz, acetone-d_6)

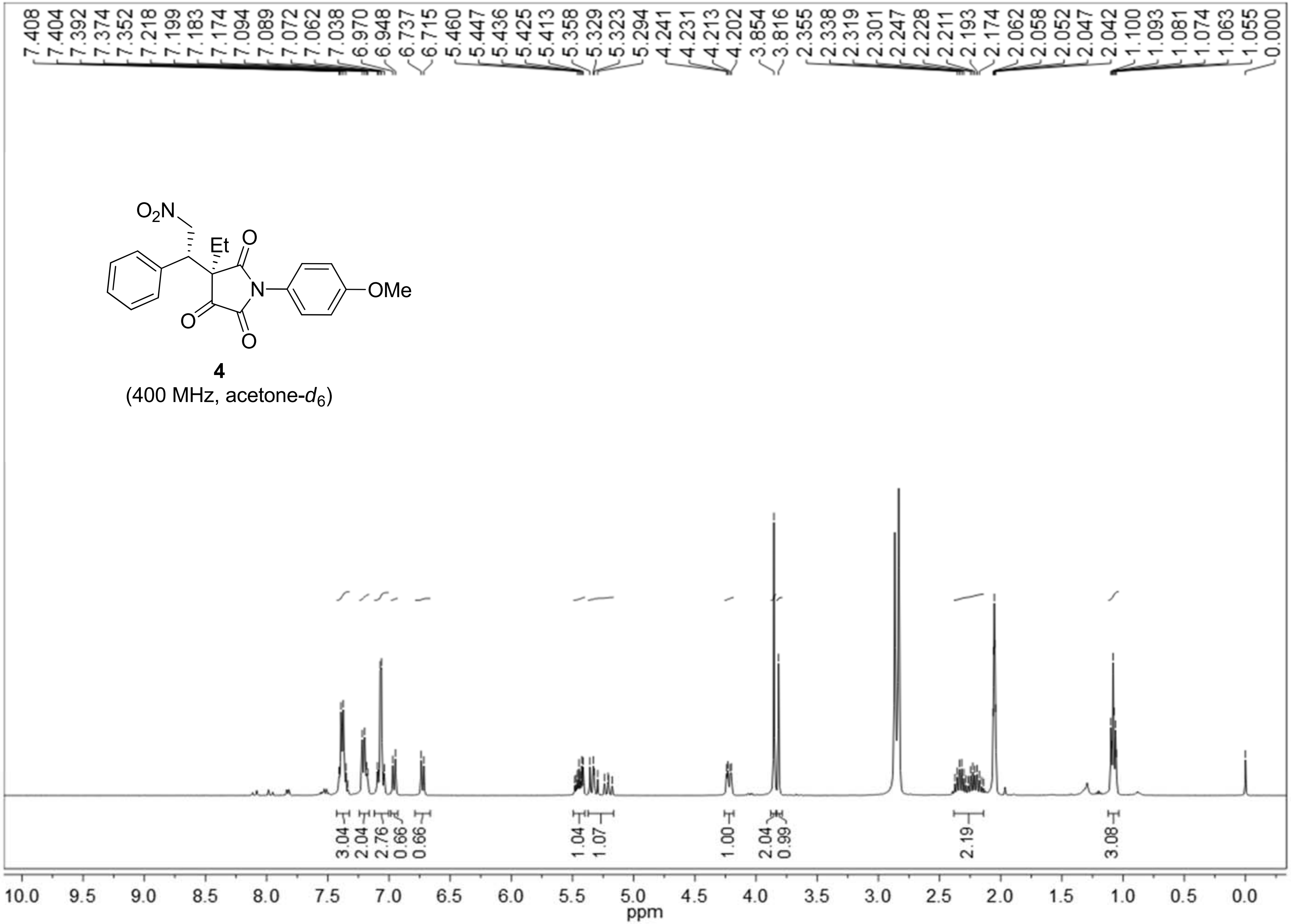
7.408 7.404 7.392 7.374 7.352 7.218 7.199 7.183 7.174 7.094 7.089 7.072 7.062 7.038 6.970 6.948 6.737 6.715 5.460 5.447 5.436 5.425 5.413 5.358 5.329 5.323 5.294 4.241 4.231 4.213 4.202 3.854 3.816 2.355 2.338 2.319 2.301 2.247 2.228 2.211 2.193 2.174 2.062 2.058 2.052 2.047 2.042 1.100 1.093 1.081 1.074 1.063 1.055 0.000
O2N
Et
O
N
OMe
O
O
4
(400 MHz, acetone-d6)
3.04 2.04 2.76 0.66 0.66 1.04 1.07 1.00 2.04 0.99 2.19 3.08
10.0 9.5 9.0 8.5 8.0 7.5 7.0 6.5 6.0 5.5 5.0 4.5 4.0 3.5 3.0 2.5 2.0 1.5 1.0 0.5 0.0
ppm

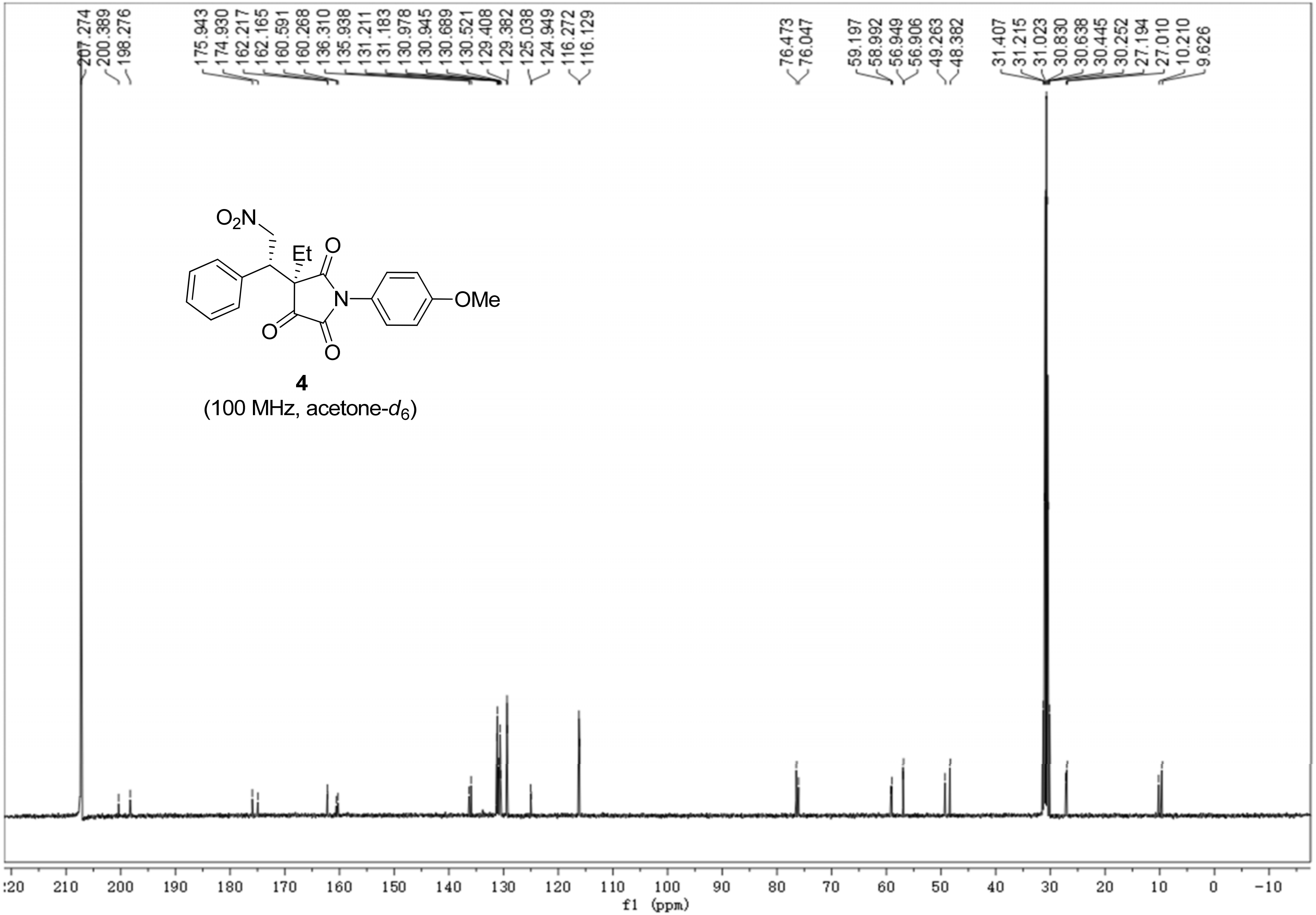
207.274
200.389
198.276
175.943
174.930
162.217
162.165
160.591
160.268
136.310
135.938
131.211
131.183
130.978
130.945
130.689
130.521
129.408
129.382
125.038
124.949
116.272
116.129
76.473
76.047
59.197
58.992
56.949
56.906
49.263
48.382
31.407
31.215
31.023
30.830
30.638
30.445
30.252
27.194
27.010
10.210
9.626
O_2N
Et
O
N
OMe
O
O
4
(100 MHz, acetone-d_6)
f1 (ppm)

2. X-ray single-crystal data for product 3ca

The single crystal of **3jca** was cultured in a mixture of dichloromethane and ethanol, the single crystal data see Table S1.

Table S1 Crystal data and structure refinement for **3ca**

Identification code	1_a (CCDC 2180149)	
Empirical formula	C21 H20 N2 O6	
Formula weight	396.39	
Temperature	293(2) K	
Wavelength	1.54178 Å	
Crystal system	Orthorhombic	
Space group	$P2_12_12_1$	
Unit cell dimensions	a = 8.3791(2) Å	α= 90°.
	b = 12.7051(3) Å	β= 90°.
	c = 17.7169(5) Å	γ = 90°.
Volume	1886.09(8) $Å^3$	
Z	4	
Density (calculated)	1.396 Mg/m^3	
Absorption coefficient	0.863 mm^{-1}	
F(000)	832	
Crystal size	0.200 x 0.200 x 0.200 mm^3	
Theta range for data collection	4.282 to 66.854°.	
Index ranges	-9<=h<=7, -15<=k<=15, -19<=l<=21	
Reflections collected	11732	
Independent reflections	3325 [R(int) = 0.0349]	
Completeness to theta = 66.854°	99.8 %	
Absorption correction	Semi-empirical from equivalents	
Refinement method	Full-matrix least-squares on F^2	
Data / restraints / parameters	3325 / 0 / 264	
Goodness-of-fit on F^2	1.095	
Final R indices [I>2sigma(I)]	R1 = 0.0361, wR2 = 0.0864	
R indices (all data)	R1 = 0.0429, wR2 = 0.0921	
Absolute structure parameter	0.02(11)	
Extinction coefficient	n/a	
Largest diff. peak and hole	0.147 and -0.156 e.$Å^{-3}$	

3. Copies of HPLC chromatograms

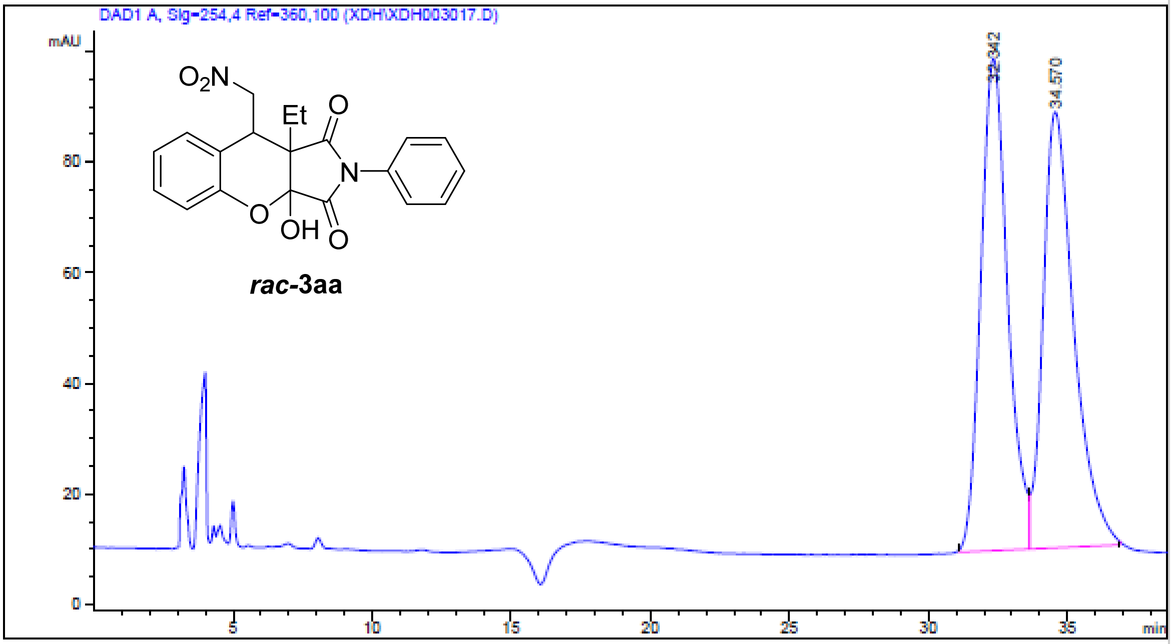

Peak #	RetTime [min]	Type	Width [min]	Area [mAU*s]	Height [mAU]	Area %
1	32.342	BV	0.9923	5980.17090	88.96080	49.5508
2	34.570	VB	1.1161	6088.60791	78.74212	50.4492

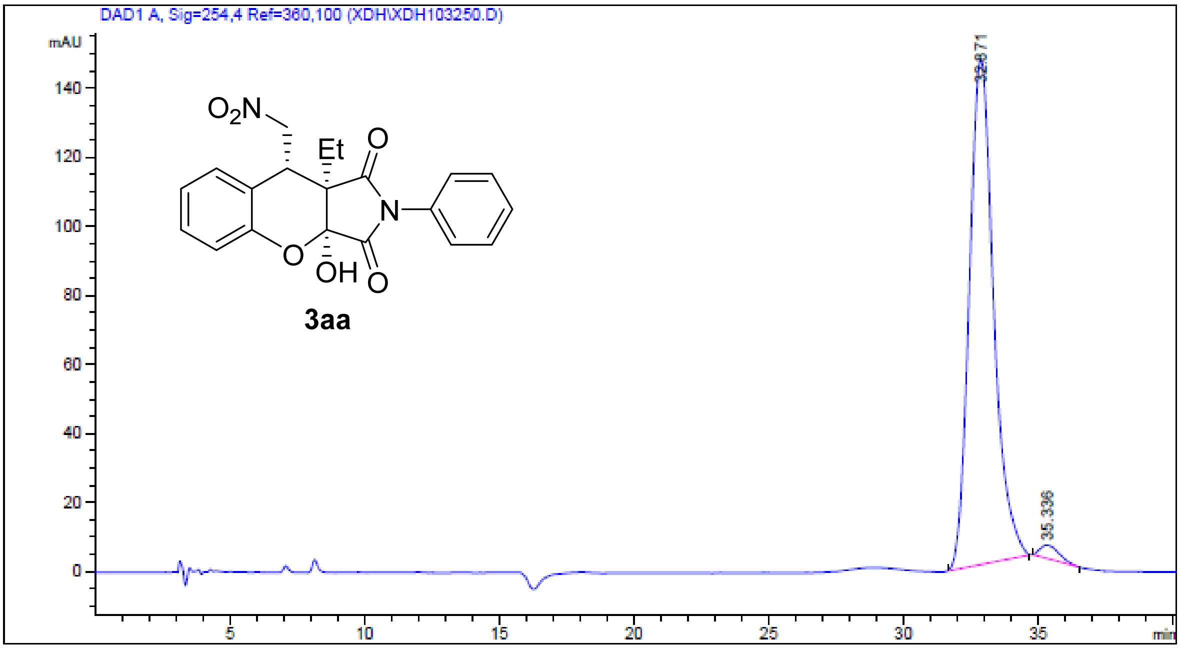

Peak #	RetTime [min]	Type	Width [min]	Area [mAU*s]	Height [mAU]	Area %
1	32.871	BB	0.9511	9159.84375	146.17348	97.9252
2	35.336	BB	0.5877	194.07497	3.89231	2.0748

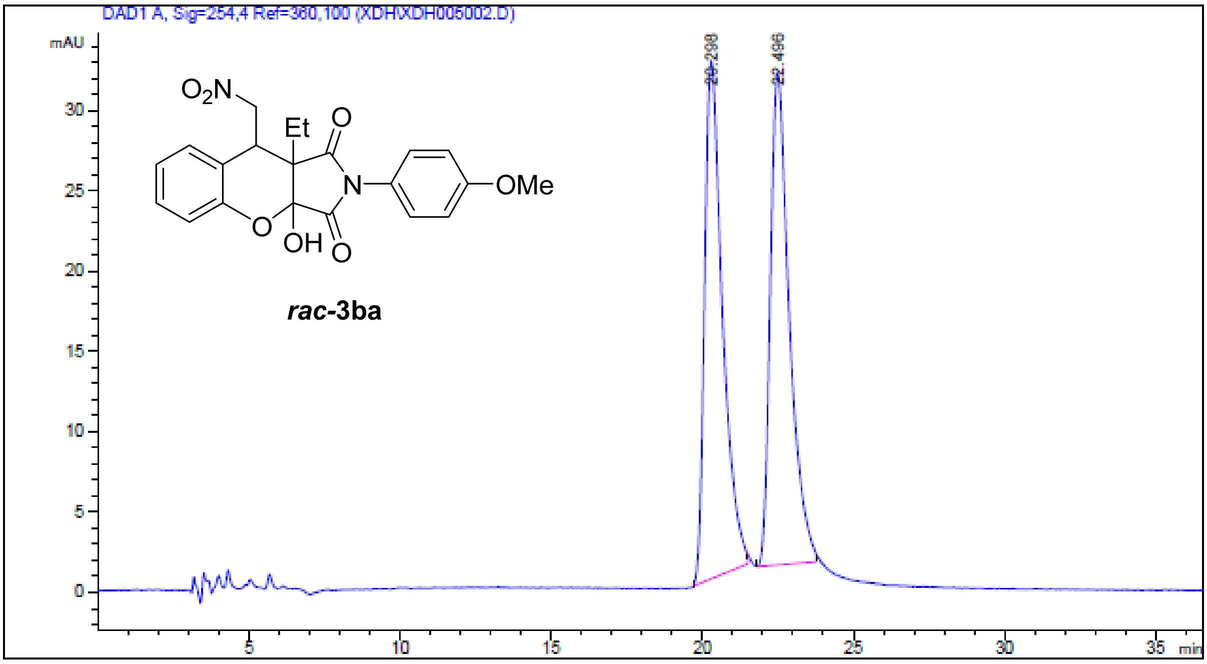

Peak #	RetTime [min]	Type	Width [min]	Area [mAU*s]	Height [mAU]	Area %
1	20.298	BB	0.6028	1329.76172	32.26576	49.8705
2	22.496	BB	0.6486	1336.66785	30.65999	50.1295

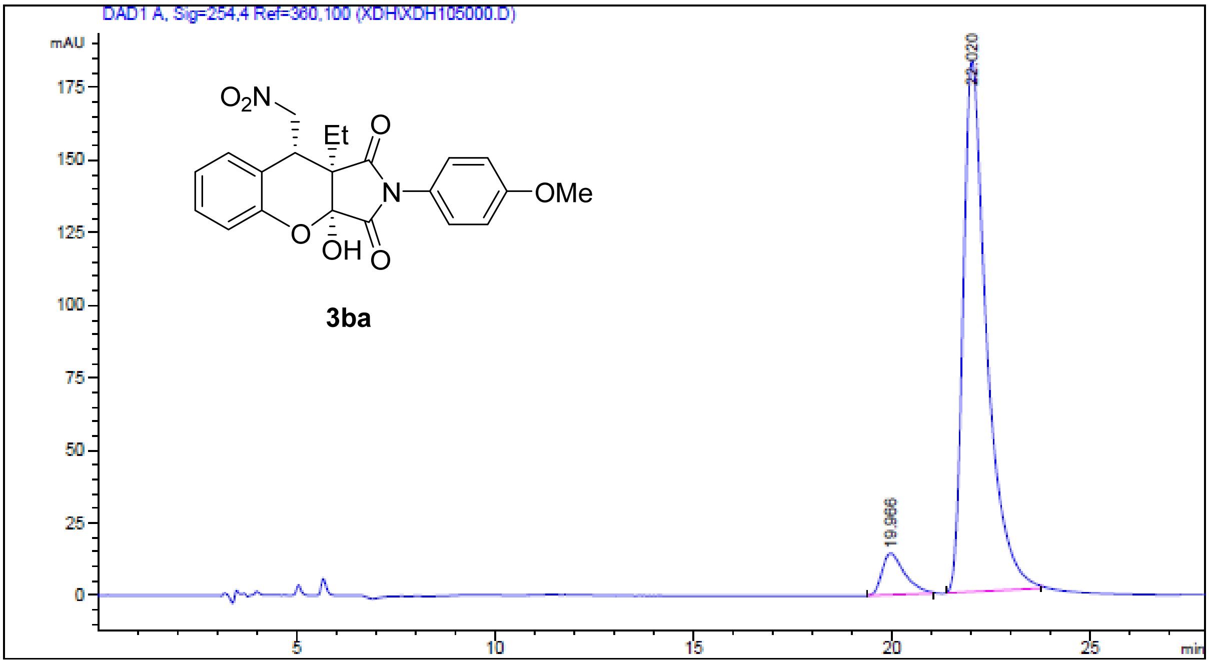

Peak #	RetTime [min]	Type	Width [min]	Area [mAU*s]	Height [mAU]	Area %
1	19.966	BB	0.5765	571.13934	14.28339	7.0508
2	22.020	BB	0.6102	7529.22217	182.94466	92.9492

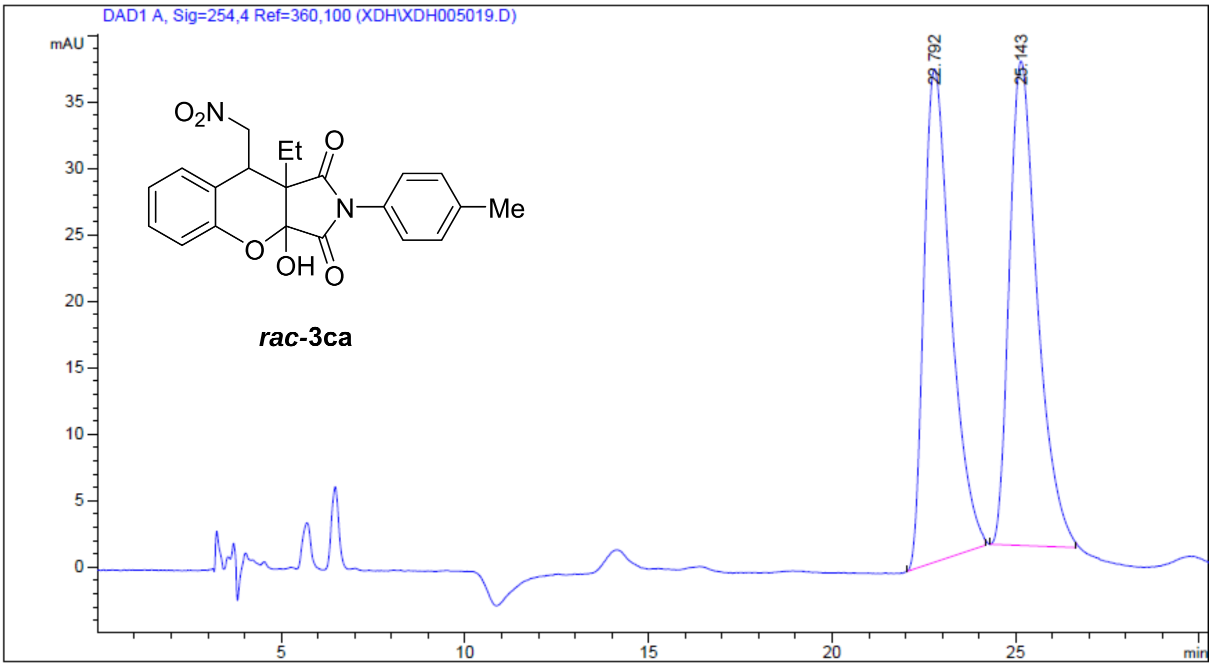

Peak #	RetTime [min]	Type	Width [min]	Area [mAU*s]	Height [mAU]	Area %
1	22.792	BB	0.7573	1899.81128	37.10545	49.5528
2	25.143	BB	0.7673	1934.10120	36.43593	50.4472

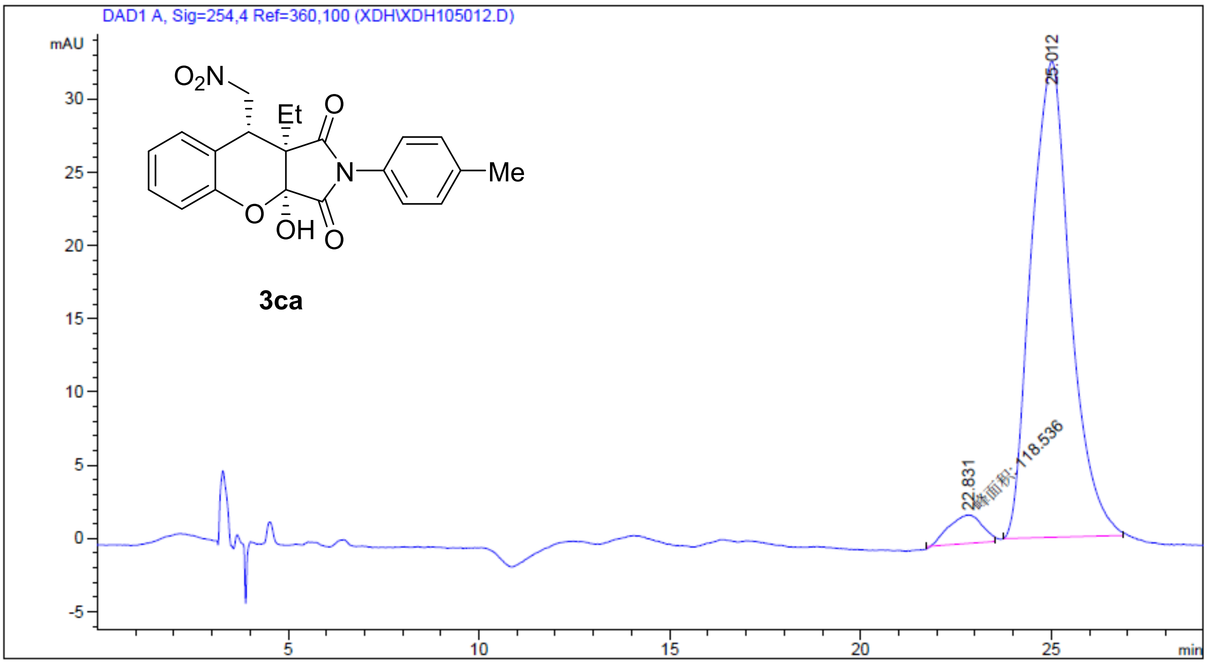

Peak #	RetTime [min]	Type	Width [min]	Area [mAU*s]	Height [mAU]	Area %
1	22.831	MM	1.0193	118.53577	1.93810	4.8570
2	25.012	BB	0.9370	2321.98022	32.52229	95.1430

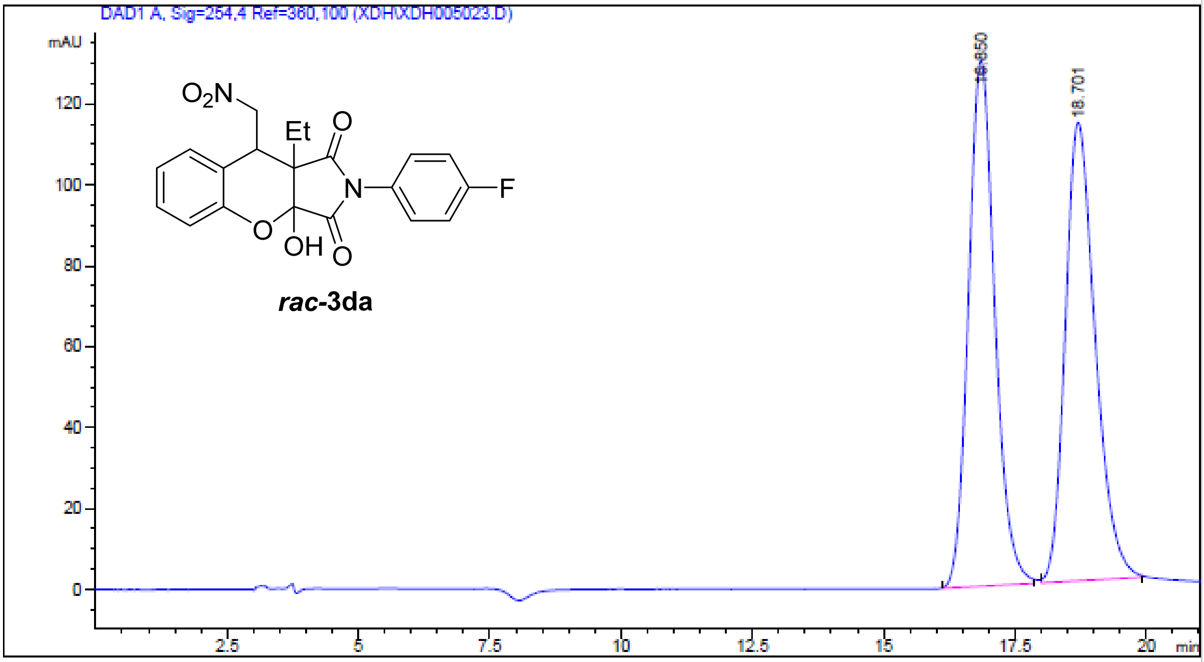

Peak #	RetTime [min]	Type	Width [min]	Area [mAU*s]	Height [mAU]	Area %
1	16.850	BB	0.5329	4538.41650	130.11980	50.1982
2	18.701	BB	0.6016	4502.57471	113.31600	49.8018

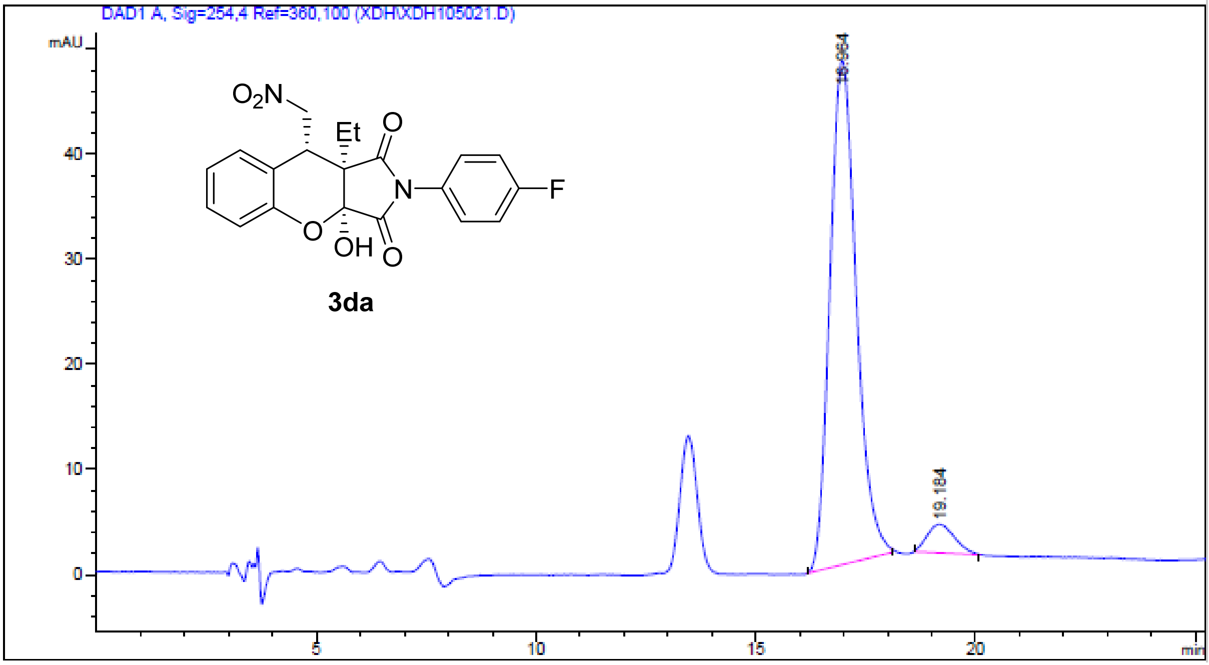

Peak #	RetTime [min]	Type	Width [min]	Area [mAU*s]	Height [mAU]	Area %
1	16.964	BB	0.6420	2008.76904	48.02846	94.4530
2	19.184	BB	0.5171	117.96961	2.73542	5.5470

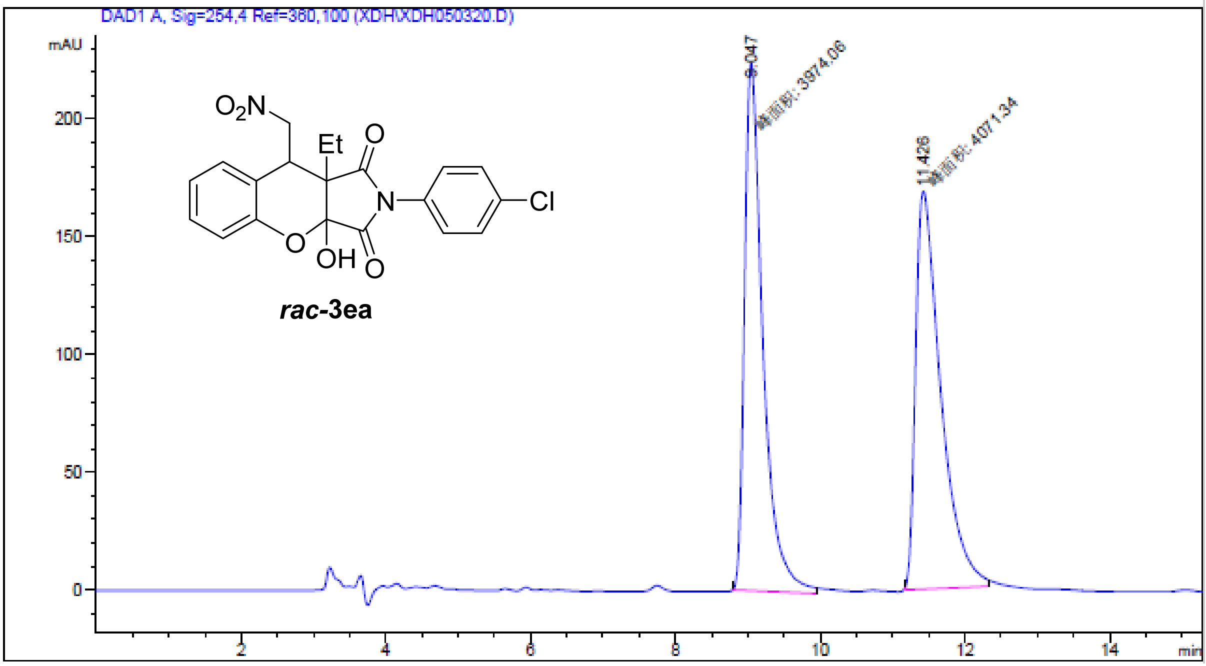

Peak #	RetTime [min]	Type	Width [min]	Area [mAU*s]	Height [mAU]	Area %
1	9.047	MM	0.2956	3974.06348	224.08592	49.3955
2	11.426	MM	0.4018	4071.34033	168.89180	50.6045

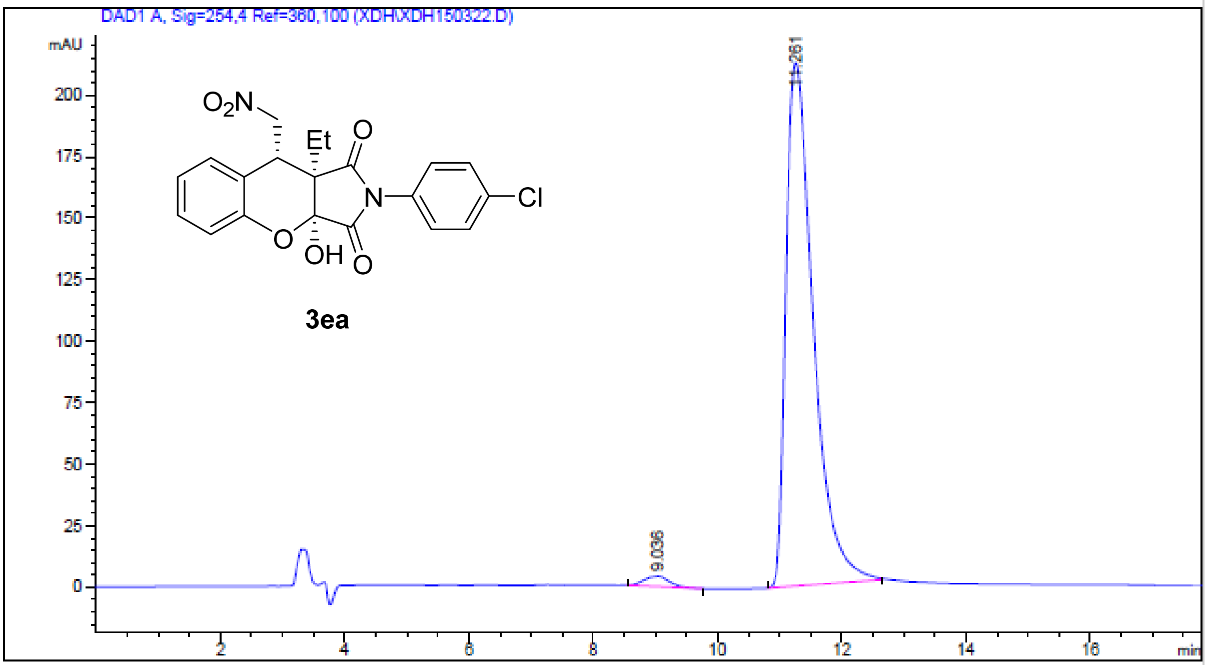

Peak #	RetTime [min]	Type	Width [min]	Area [mAU*s]	Height [mAU]	Area %
1	9.036	BB	0.4219	117.15128	4.19438	1.7706
2	11.261	BB	0.4685	6499.39746	212.72102	98.2294

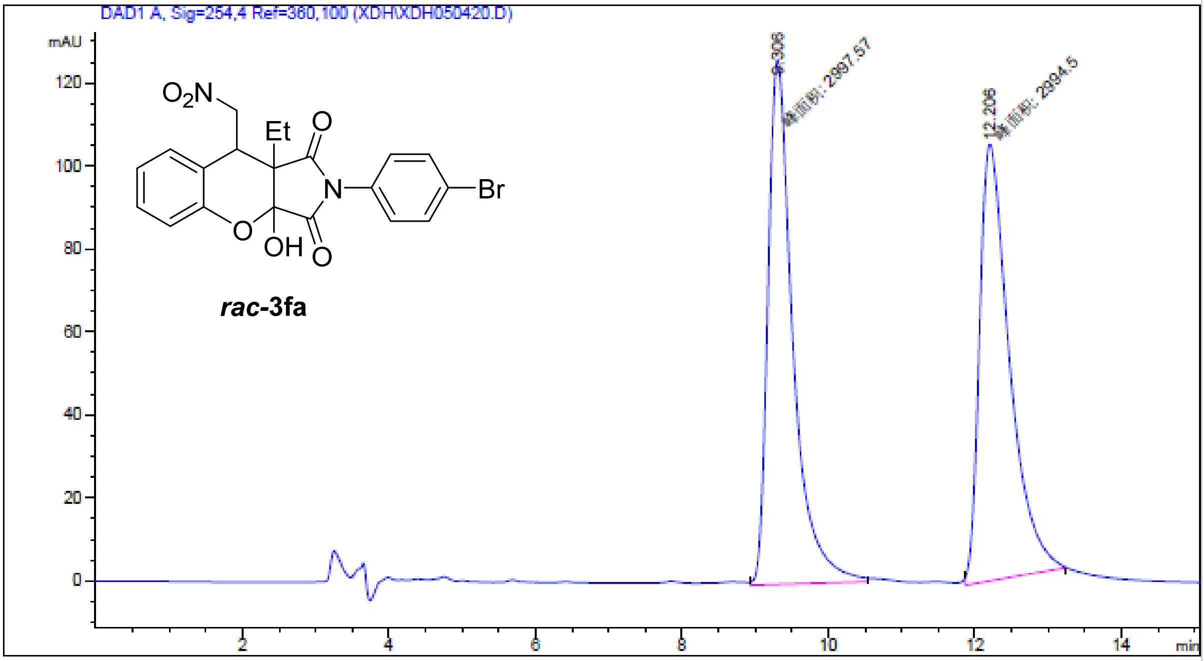

Peak #	RetTime [min]	Type	Width [min]	Area [mAU*s]	Height [mAU]	Area %
1	9.306	MM	0.3952	2997.57080	126.42984	50.0256
2	12.206	MM	0.4741	2994.50366	105.27594	49.9744

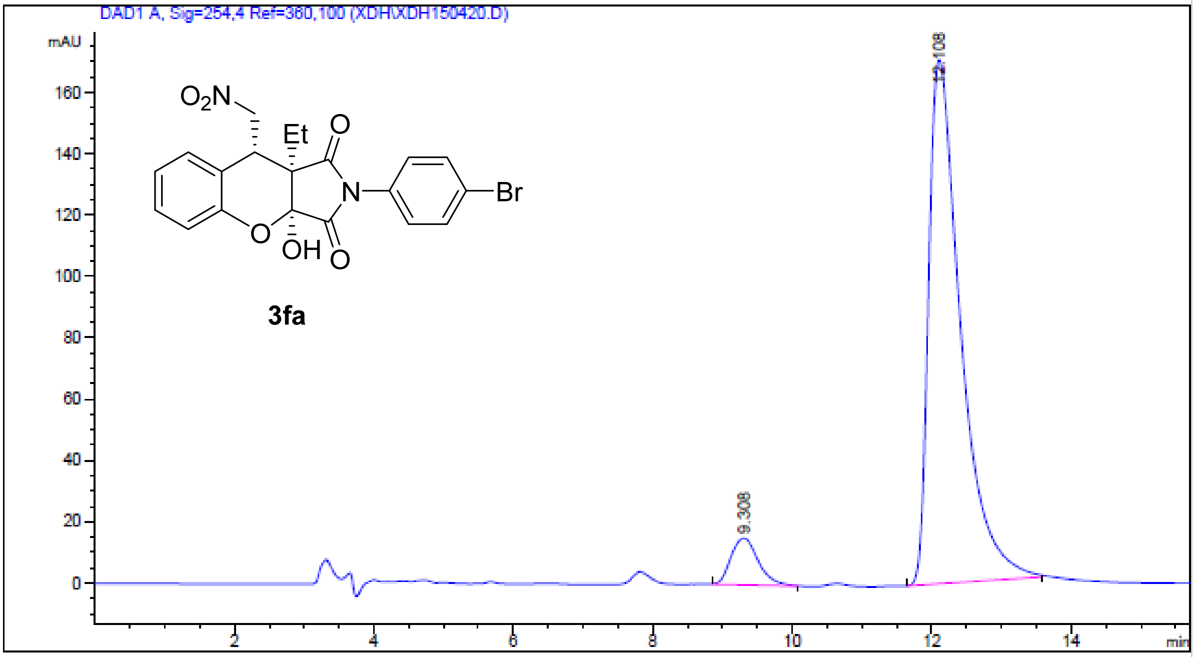

Peak #	RetTime [min]	Type	Width [min]	Area [mAU*s]	Height [mAU]	Area %
1	9.308	BB	0.4321	414.33841	15.29143	7.0695
2	12.108	BB	0.4778	5446.61279	170.89473	92.9305

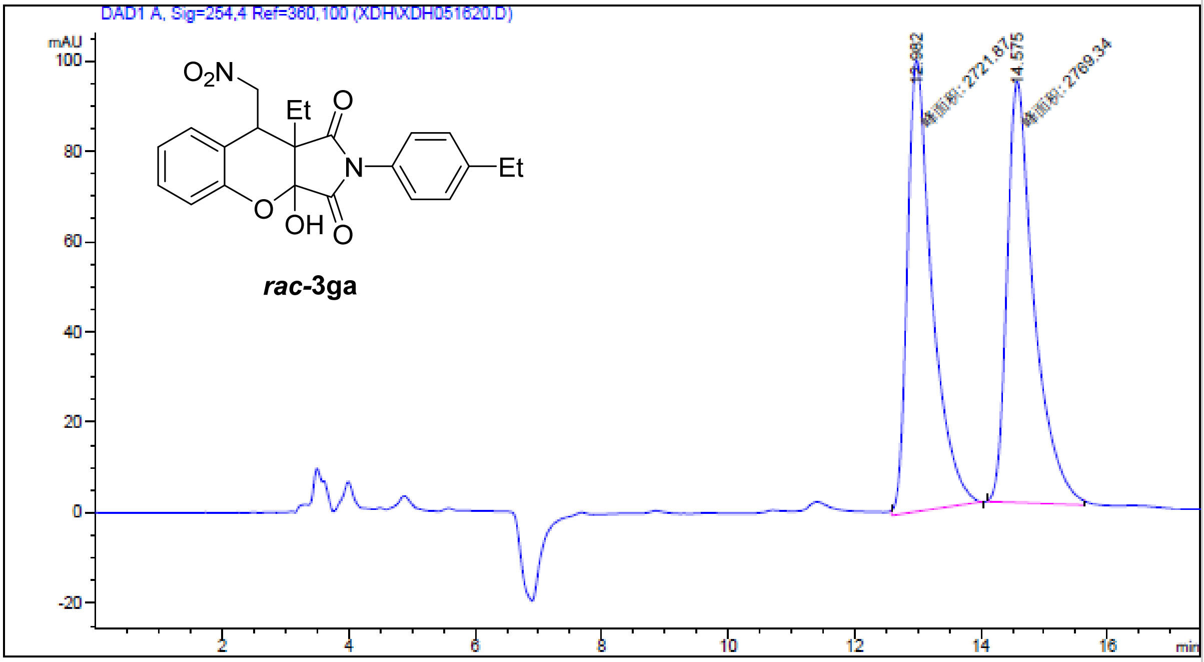

Peak #	RetTime [min]	Type	Width [min]	Area [mAU*s]	Height [mAU]	Area %
1	12.982	MM	0.4540	2721.86816	99.93094	49.5678
2	14.575	MM	0.4949	2769.33862	93.25630	50.4322

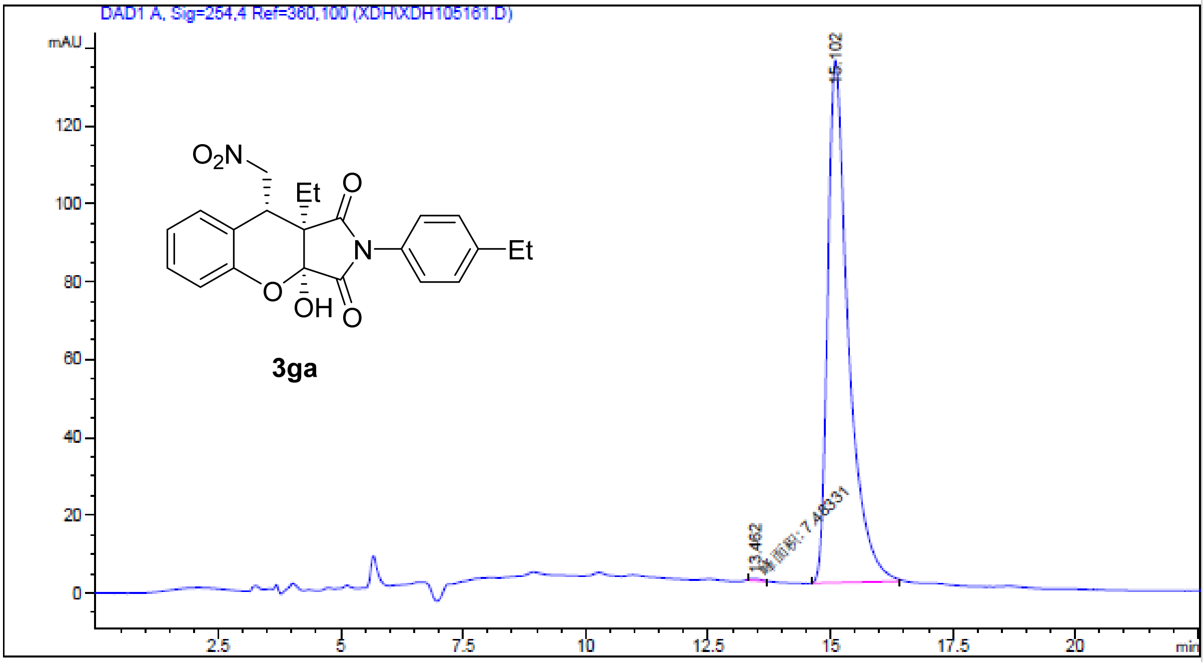

Peak #	RetTime [min]	Type	Width [min]	Area [mAU*s]	Height [mAU]	Area %
1	13.462	MM	0.2671	7.48331	4.66968e-1	0.1897
2	15.102	BB	0.4303	3938.19531	134.22505	99.8103

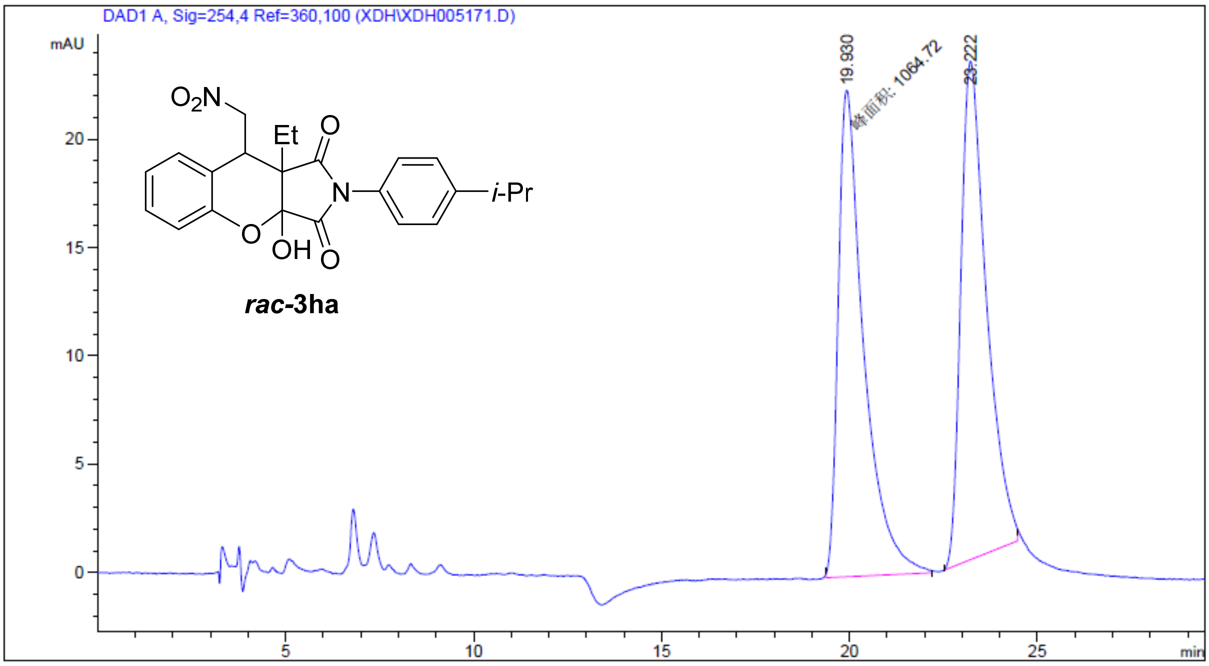

Peak #	RetTime [min]	Type	Width [min]	Area [mAU*s]	Height [mAU]	Area %
1	19.930	MM	0.7902	1064.71912	22.45747	49.4075
2	23.222	BB	0.6890	1090.25598	23.00284	50.5925

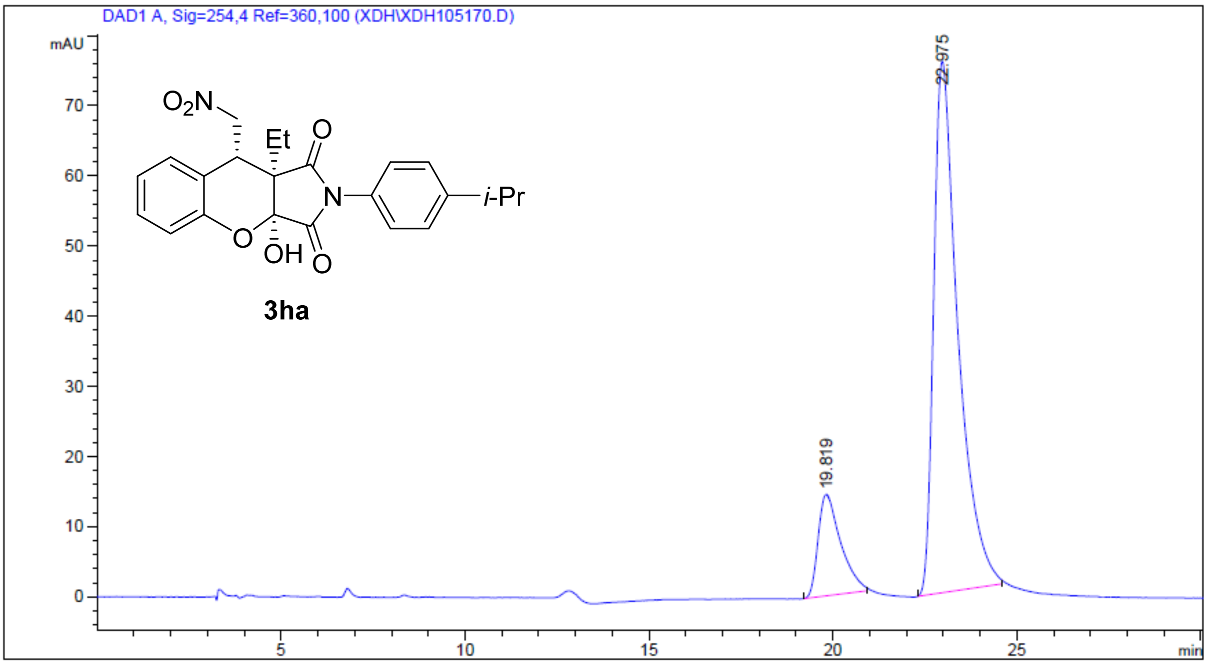

Peak #	RetTime [min]	Type	Width [min]	Area [mAU*s]	Height [mAU]	Area %
1	19.819	BB	0.6193	615.50854	14.49920	15.0384
2	22.975	BB	0.6675	3477.40991	75.76347	84.9616

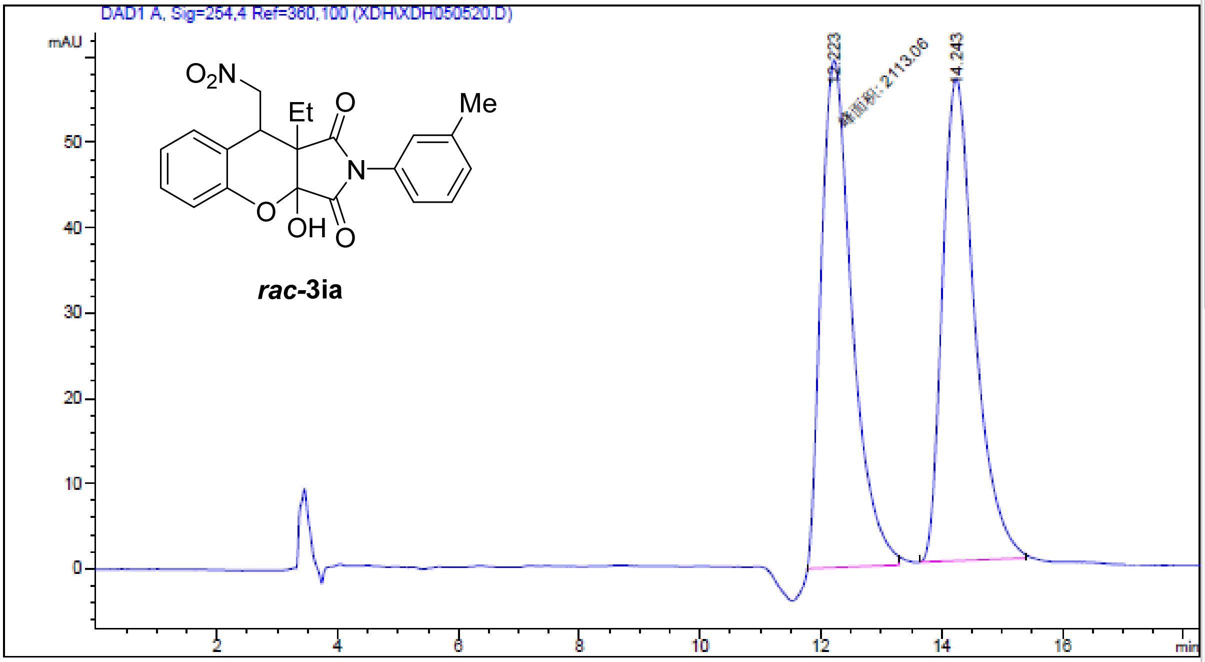

Peak #	RetTime [min]	Type	Width [min]	Area [mAU*s]	Height [mAU]	Area %
1	12.223	MM	0.5920	2113.05688	59.48728	50.0819
2	14.243	BB	0.5709	2106.14258	56.50469	49.9181

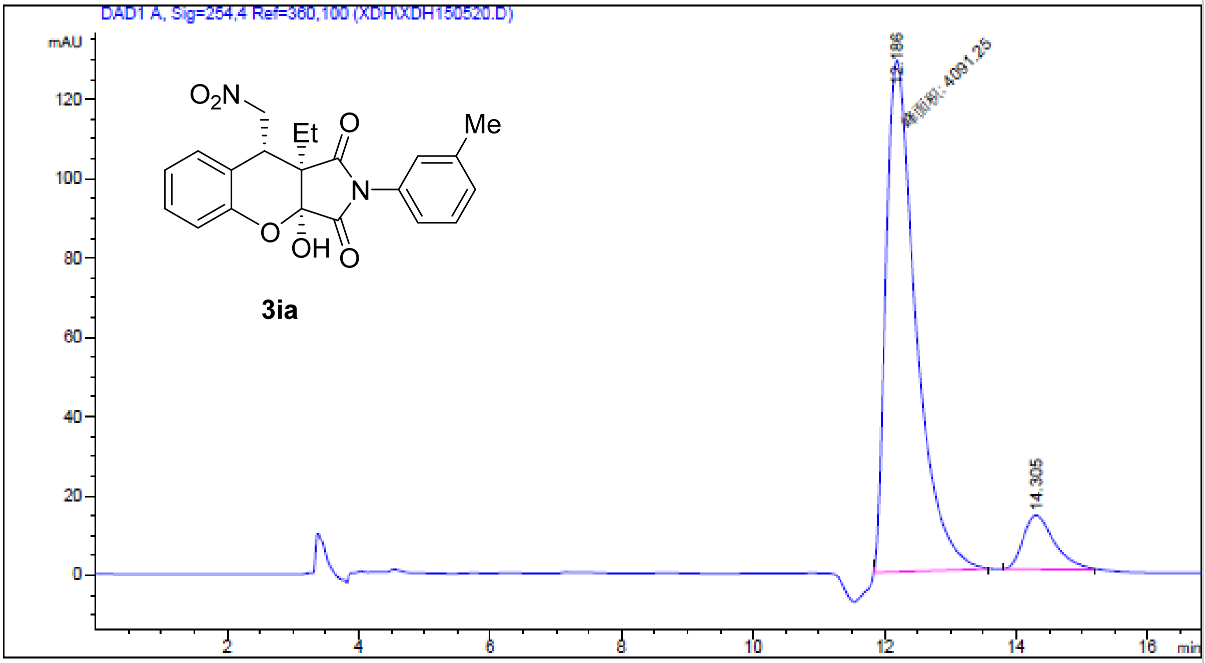

Peak #	RetTime [min]	Type	Width [min]	Area [mAU*s]	Height [mAU]	Area %
1	12.186	MM	0.5285	4091.24536	129.01772	90.0267
2	14.305	BB	0.4892	453.23303	13.65744	9.9733

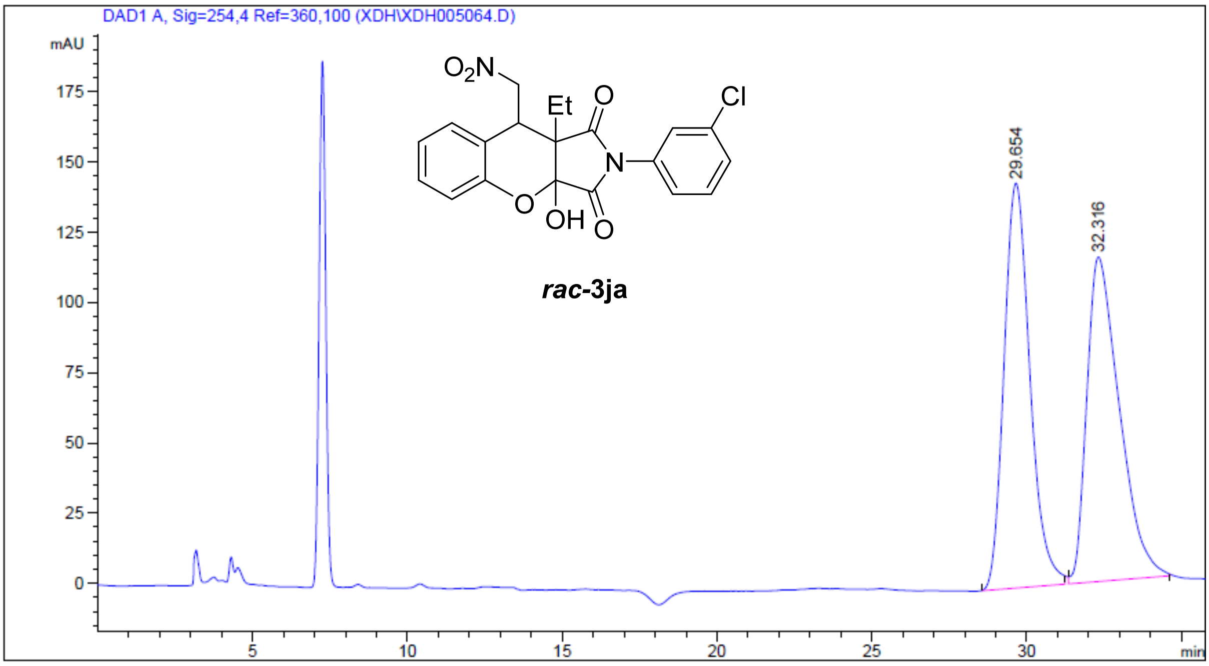

Peak #	RetTime [min]	Type	Width [min]	Area [mAU*s]	Height [mAU]	Area %
1	29.654	BB	0.8936	8373.10547	144.17351	49.7571
2	32.316	BB	1.0716	8454.86523	115.55464	50.2429

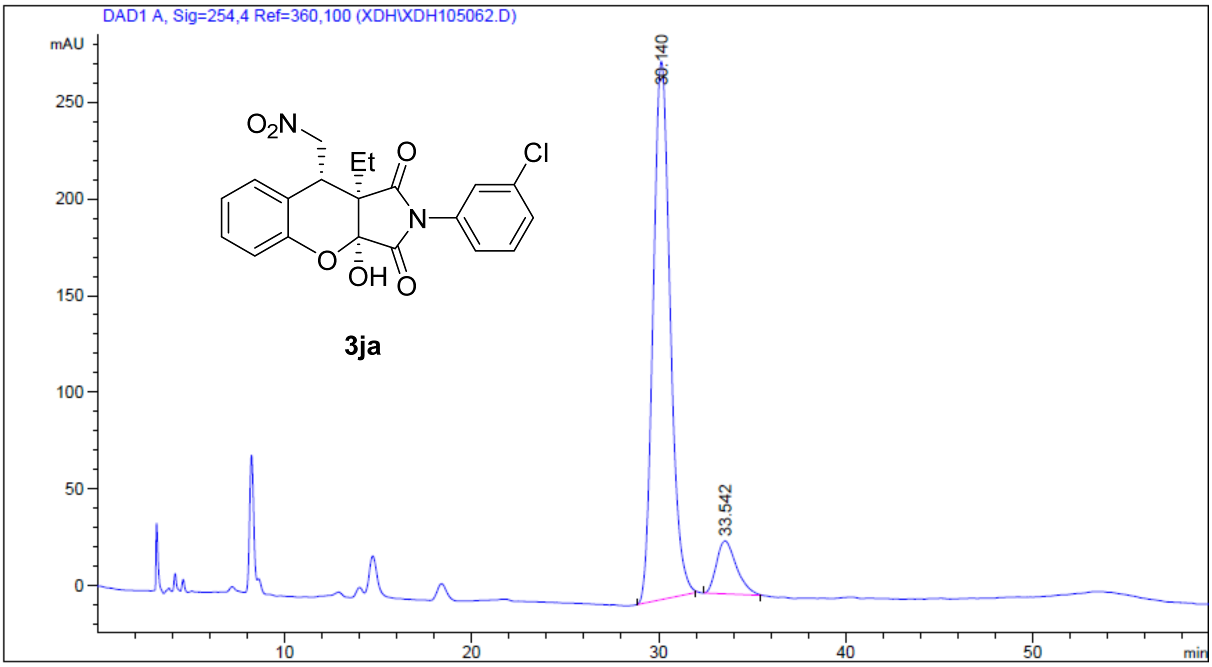

Peak #	RetTime [min]	Type	Width [min]	Area [mAU*s]	Height [mAU]	Area %
1	30.140	BB	0.9743	1.76262e4	278.53522	89.6630
2	33.542	BB	1.0151	2032.08240	27.53030	10.3370

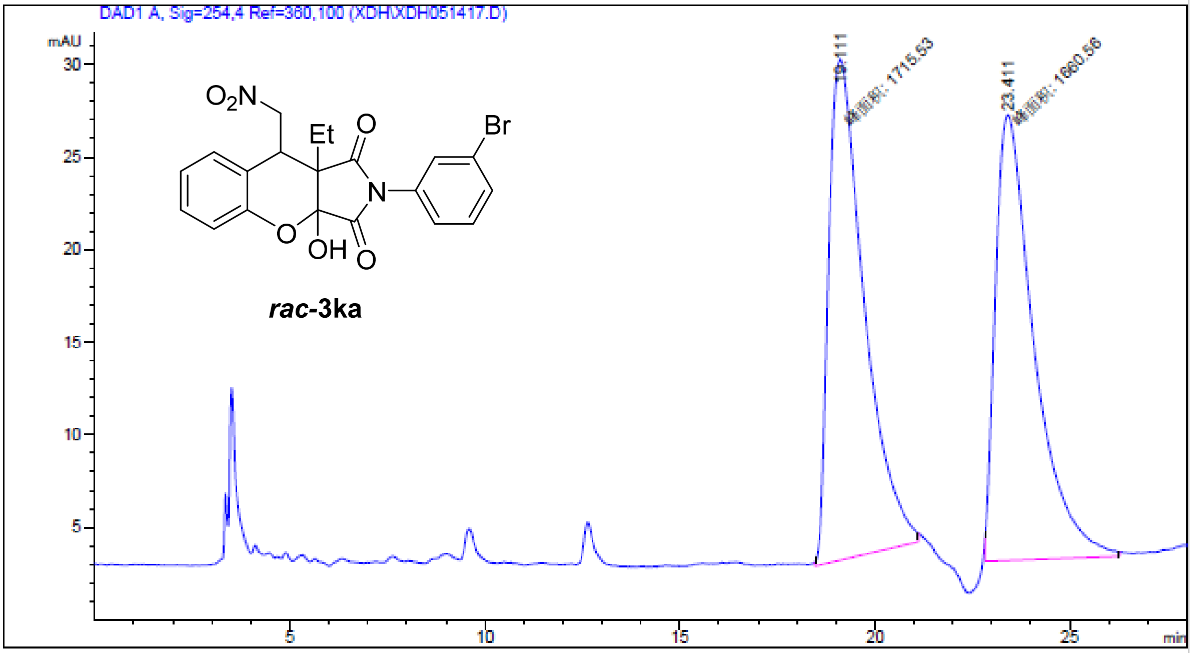

Peak #	RetTime [min]	Type	Width [min]	Area [mAU*s]	Height [mAU]	Area %
1	19.111	MM	1.0563	1715.53442	27.06934	50.8141
2	23.411	MM	1.1507	1660.56433	24.05223	49.1859

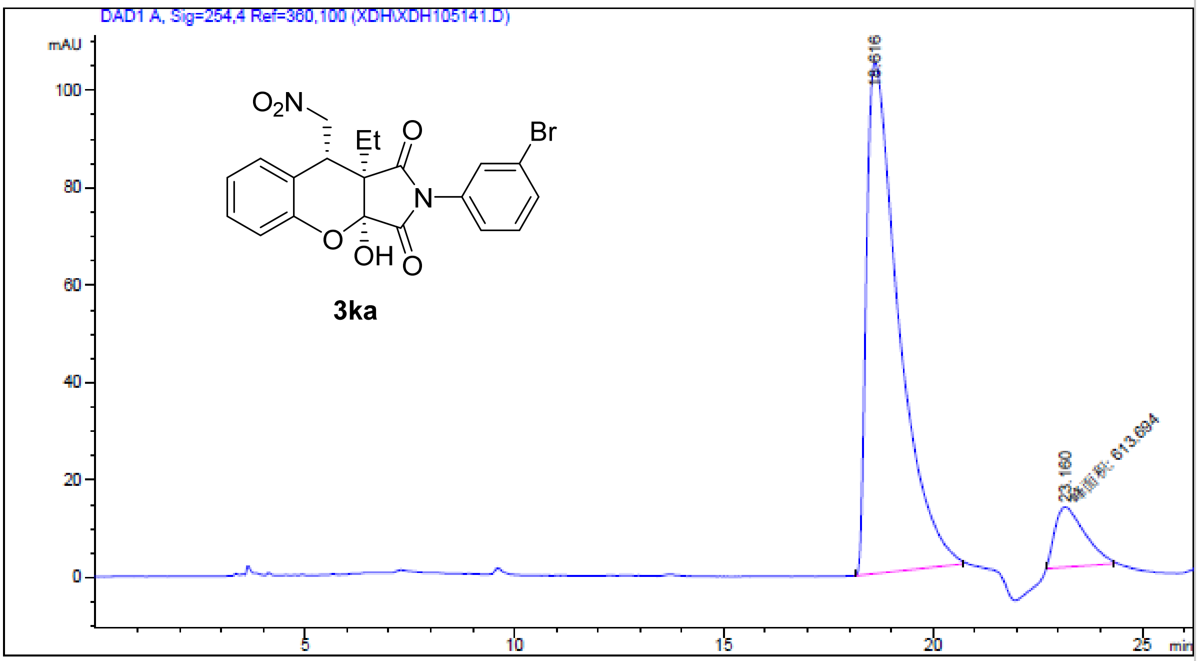

Peak #	RetTime [min]	Type	Width [min]	Area [mAU*s]	Height [mAU]	Area %
1	18.616	BB	0.7989	5620.18506	104.92124	90.1555
2	23.160	MM	0.6054	613.69373	12.35455	9.8445

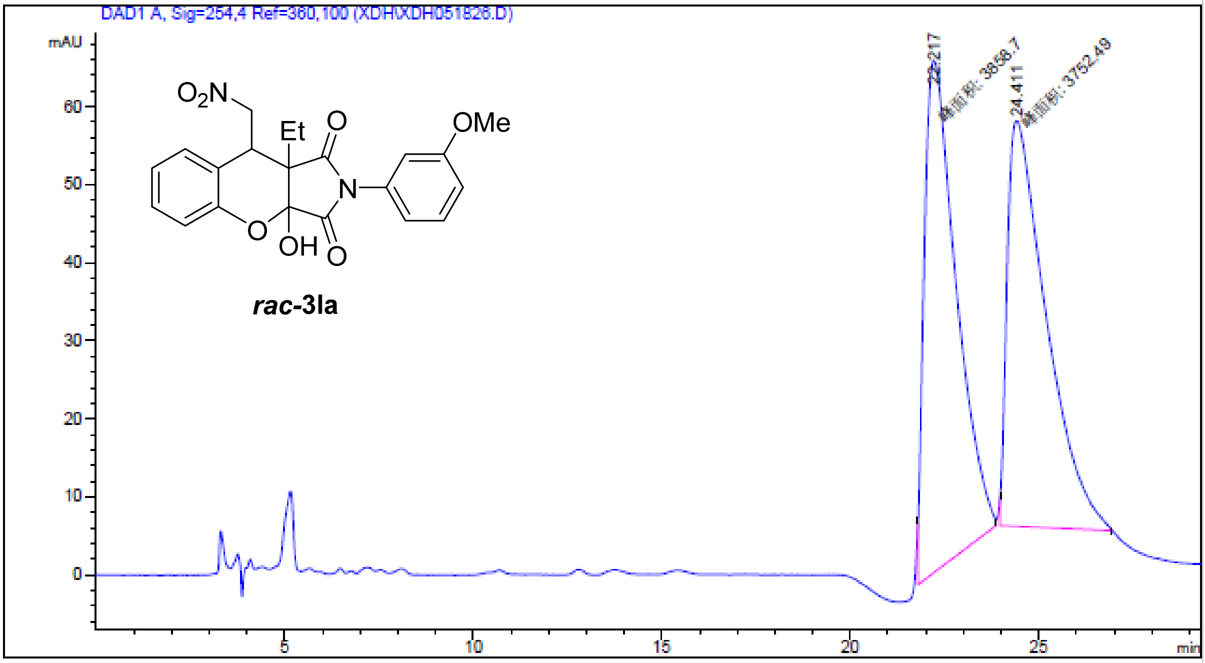

Peak #	RetTime [min]	Type	Width [min]	Area [mAU*s]	Height [mAU]	Area %
1	22.217	MM	0.9794	3858.70410	65.66515	50.6977
2	24.411	MM	1.2034	3752.49487	51.97158	49.3023

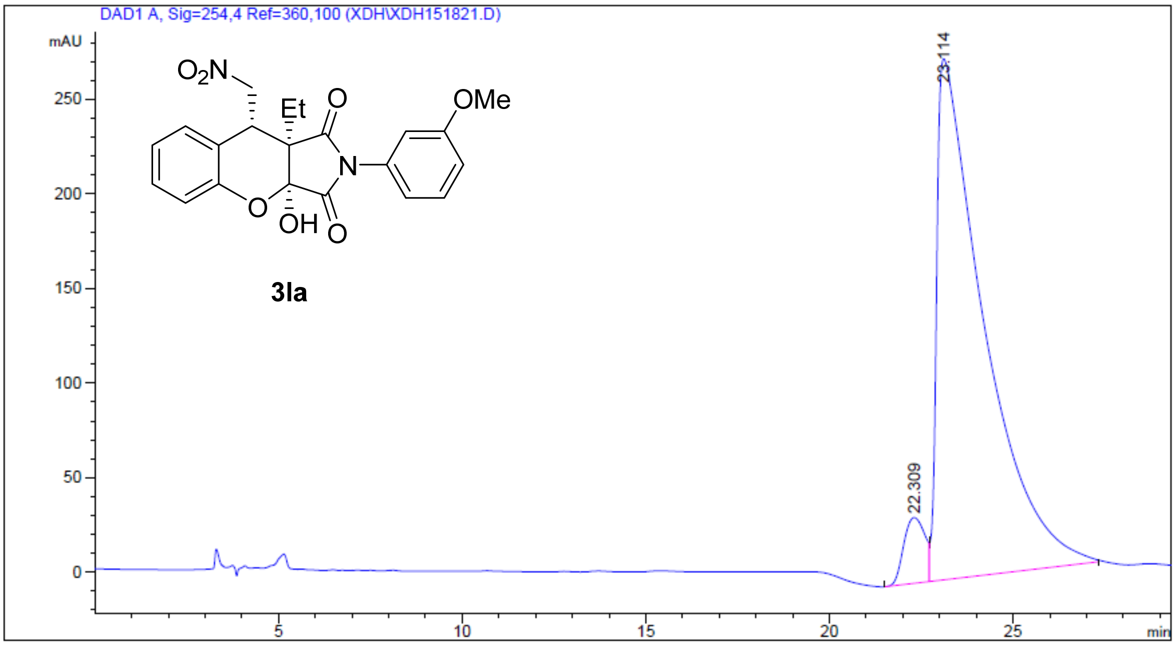

Peak #	RetTime [min]	Type	Width [min]	Area [mAU*s]	Height [mAU]	Area %
1	22.309	BV	0.6263	1381.82898	34.72271	5.3306
2	23.114	VB	1.1777	2.45405e4	275.38324	94.6694

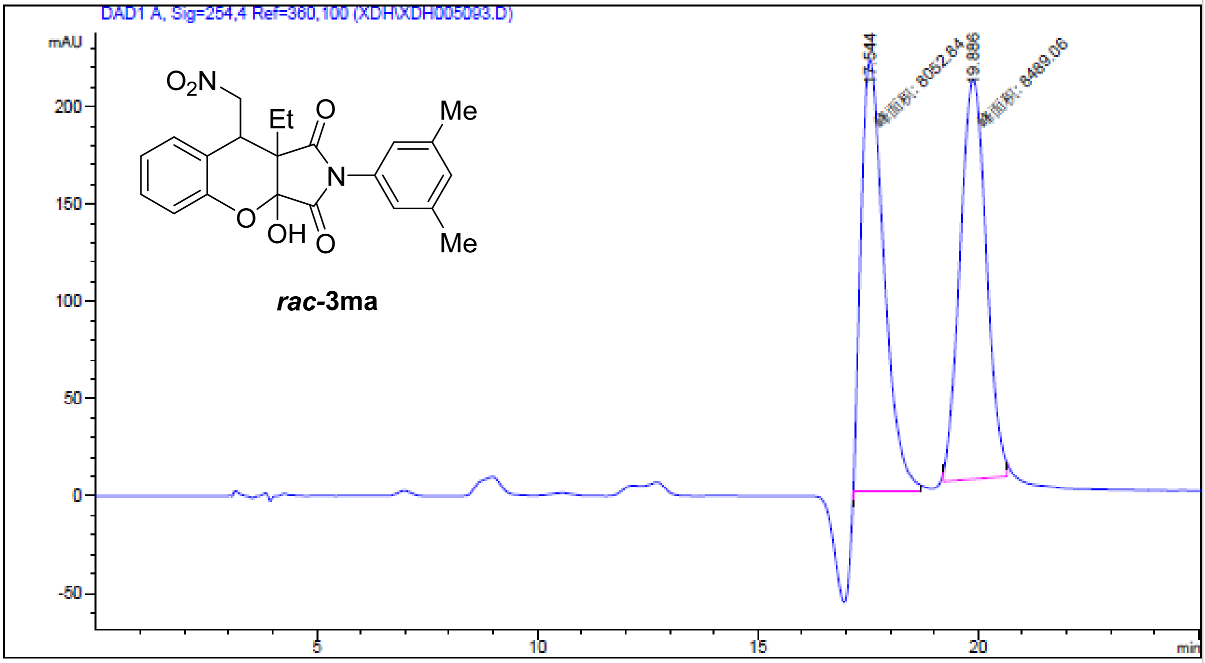

Peak #	RetTime [min]	Type	Width [min]	Area [mAU*s]	Height [mAU]	Area %
1	17.544	MM	0.6061	8052.84229	221.43750	48.6815
2	19.886	MM	0.6899	8489.05762	205.09290	51.3185

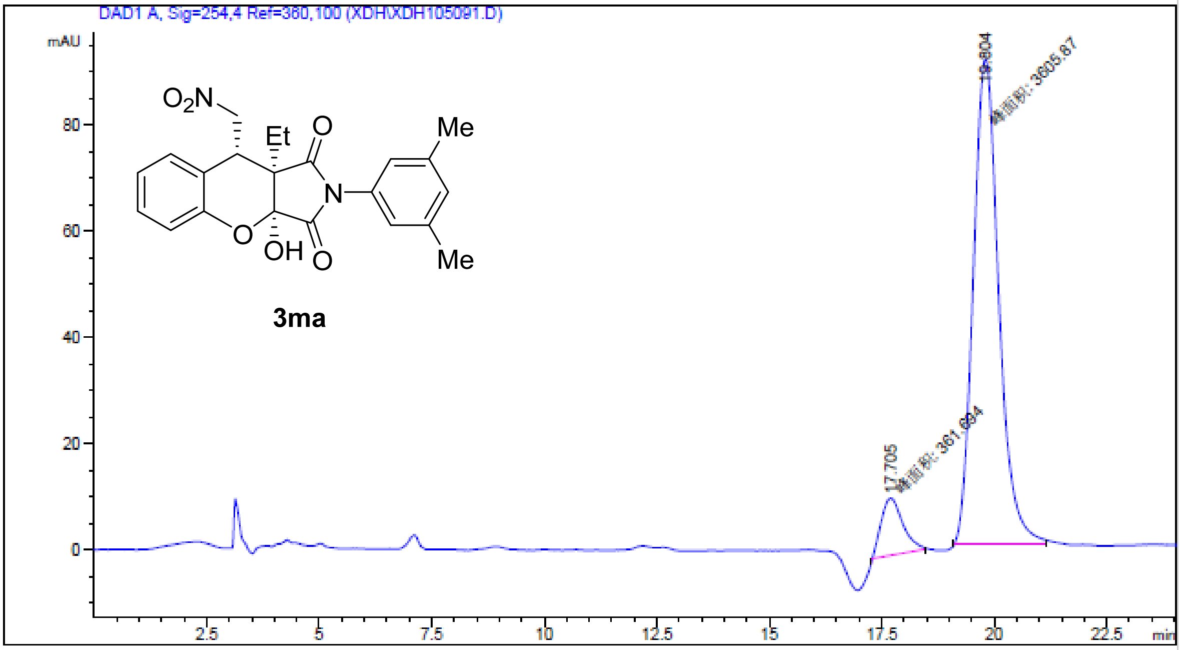

Peak #	RetTime [min]	Type	Width [min]	Area [mAU*s]	Height [mAU]	Area %
1	17.705	MM	0.5600	361.69357	10.76471	9.1163
2	19.804	MM	0.6568	3605.87134	91.49642	90.8837

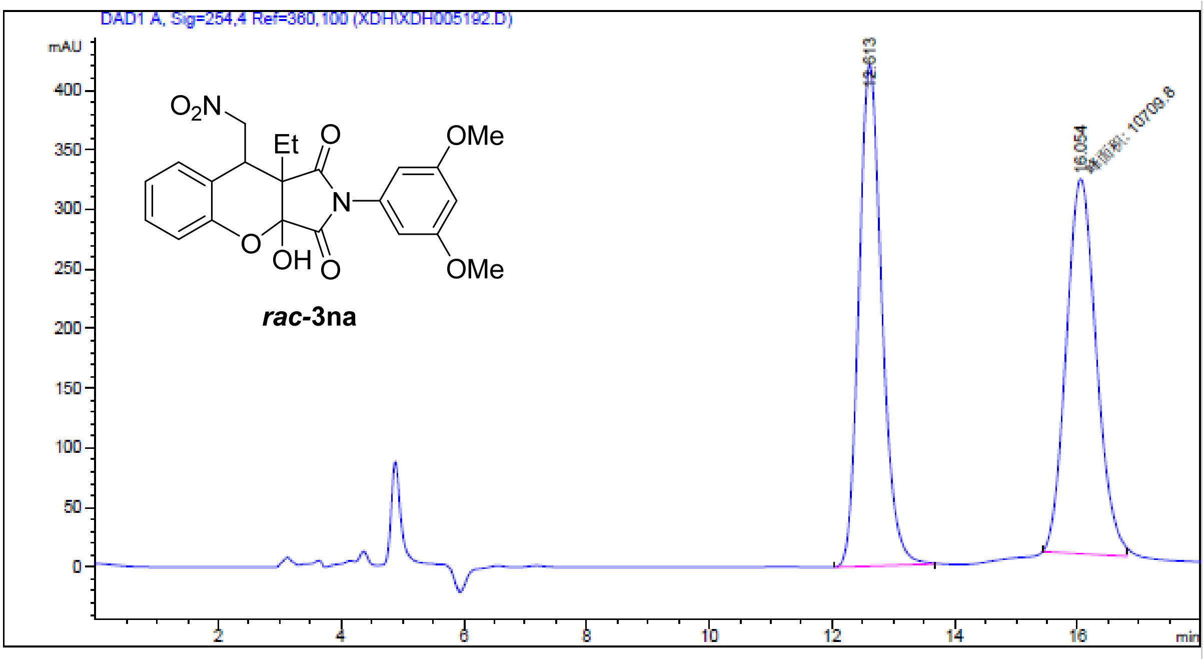

Peak #	RetTime [min]	Type	Width [min]	Area [mAU*s]	Height [mAU]	Area %
1	12.613	BB	0.4018	1.09574e4	420.83969	50.5713
2	16.054	MM	0.5679	1.07098e4	314.30490	49.4287

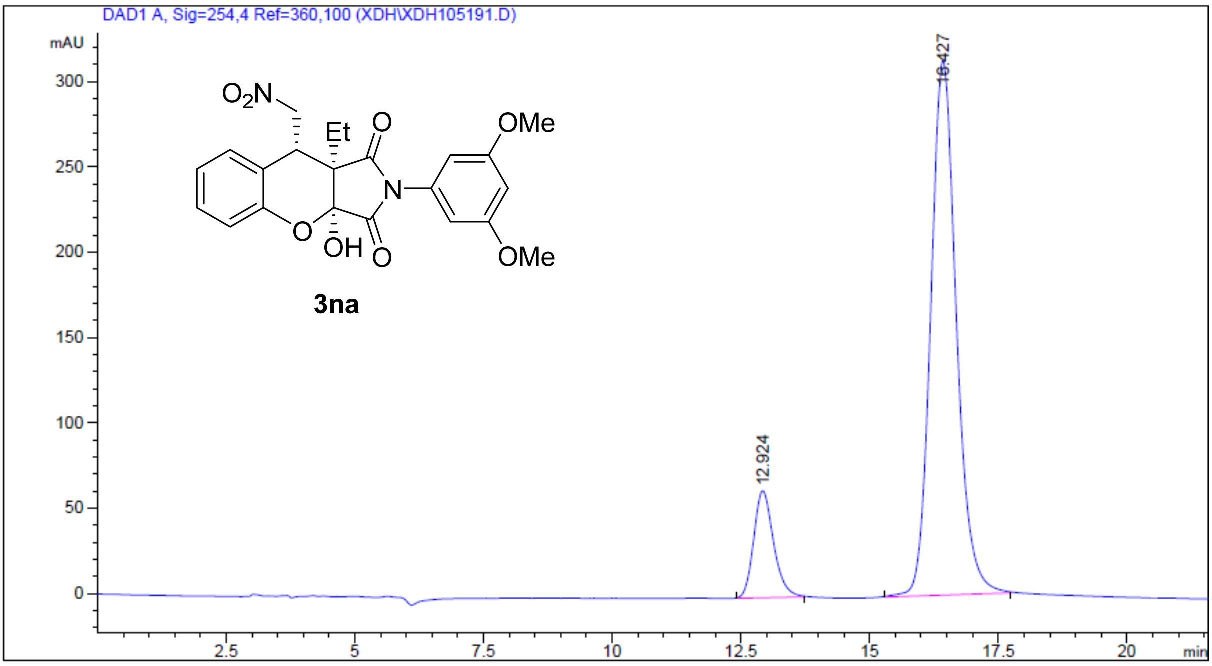

Peak #	RetTime [min]	Type	Width [min]	Area [mAU*s]	Height [mAU]	Area %
1	12.924	BB	0.4114	1684.10657	62.69512	13.4711
2	16.427	BB	0.5310	1.08175e4	313.13589	86.5289

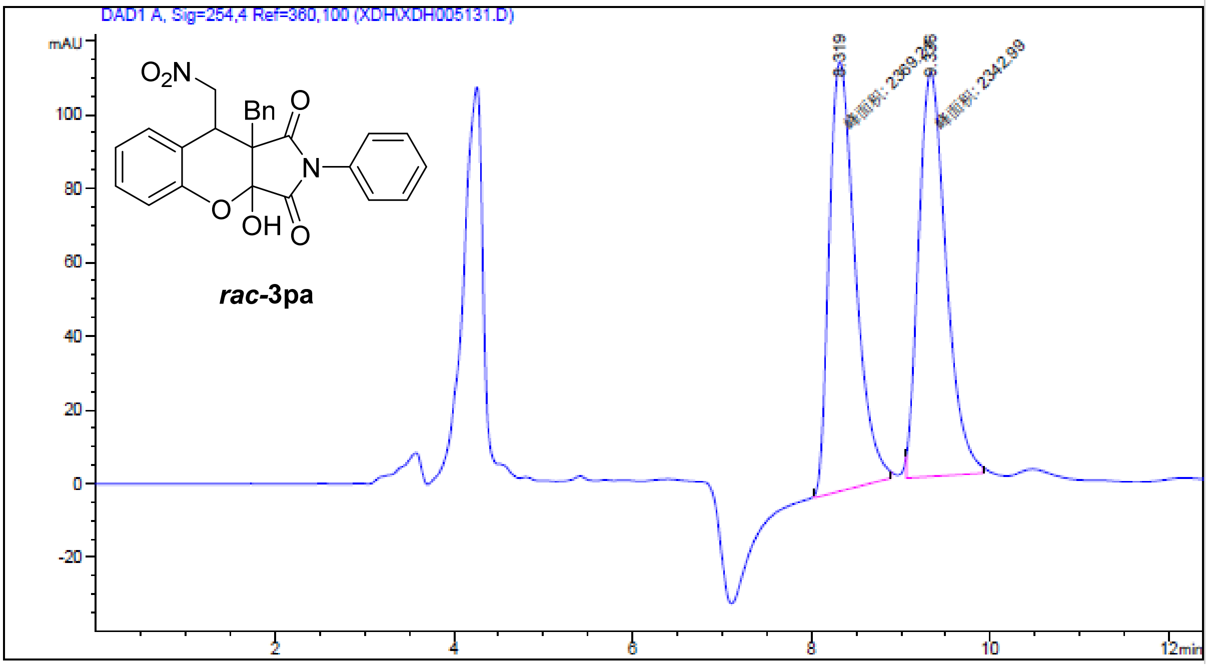

Peak #	RetTime [min]	Type	Width [min]	Area [mAU*s]	Height [mAU]	Area %
1	8.319	MM	0.3391	2369.20703	116.43809	50.2782
2	9.336	MM	0.3560	2342.99170	109.68989	49.7218

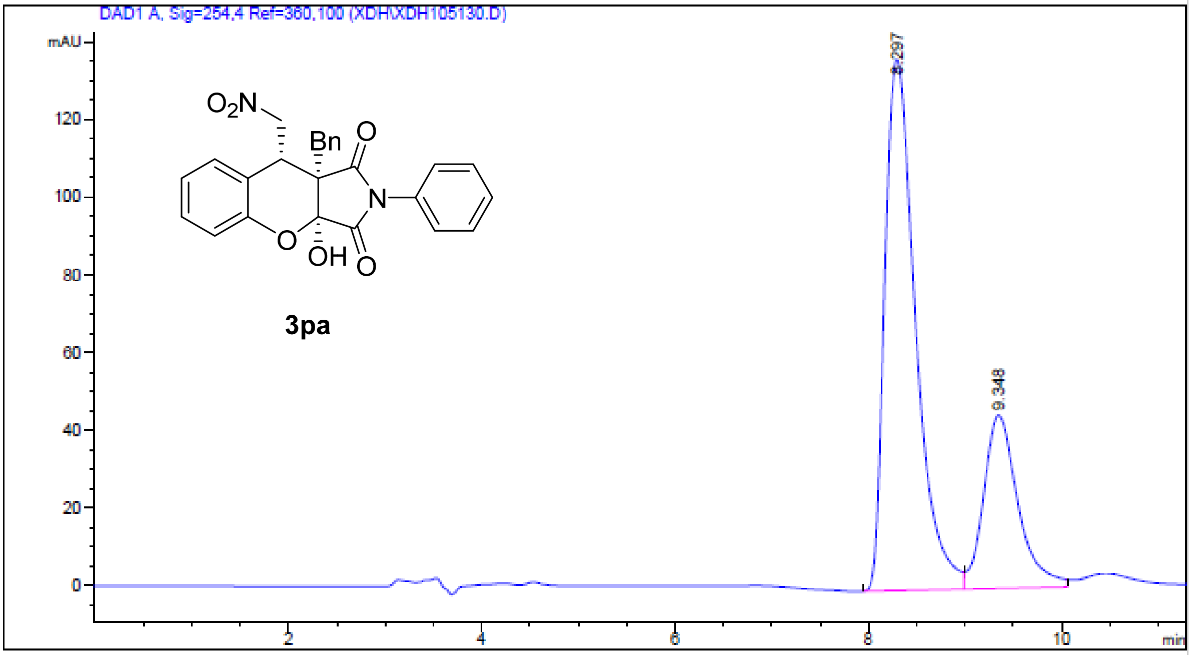

Peak #	RetTime [min]	Type	Width [min]	Area [mAU*s]	Height [mAU]	Area %
1	8.297	BV	0.3416	3017.20728	136.66194	73.1828
2	9.348	VB	0.3753	1105.63049	44.58657	26.8172

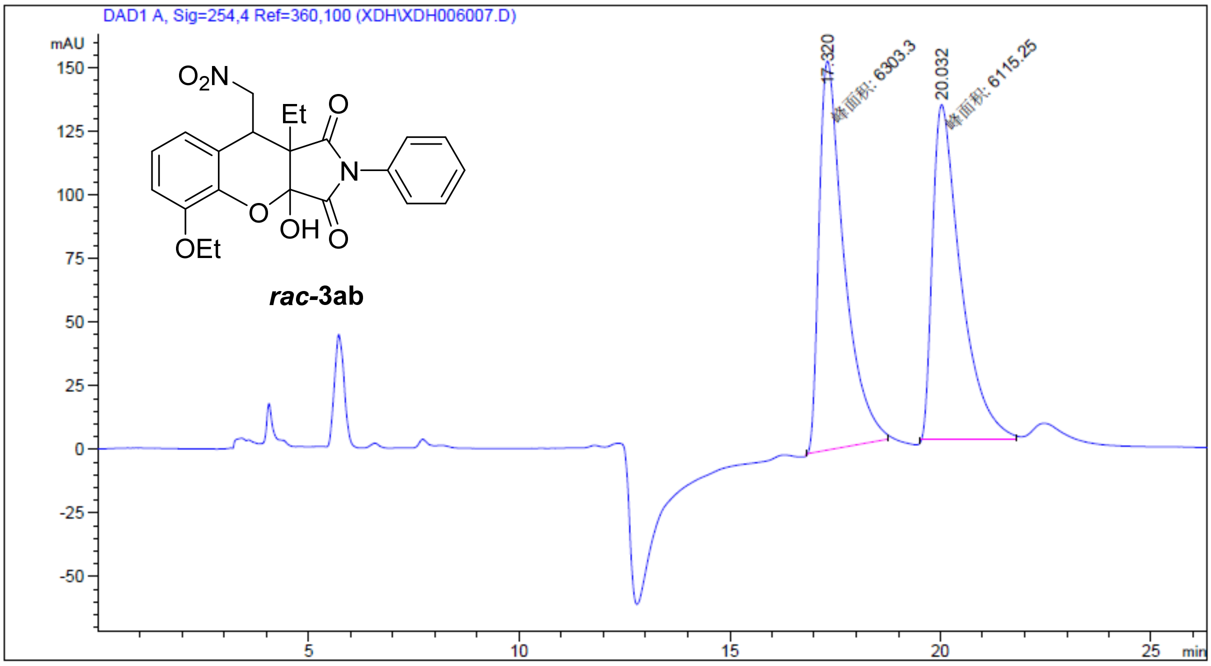

Peak #	RetTime [min]	Type	Width [min]	Area [mAU*s]	Height [mAU]	Area %
1	17.320	MM	0.6870	6303.29785	152.91806	50.7571
2	20.032	MM	0.7754	6115.24512	131.44543	49.2429

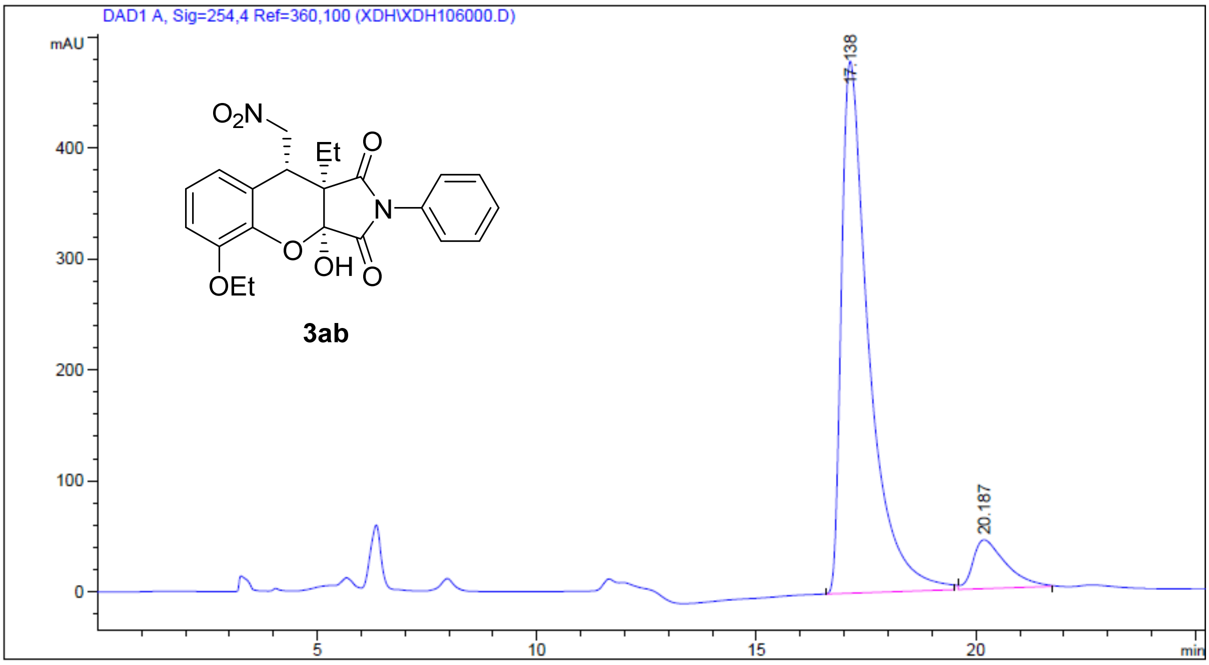

Peak #	RetTime [min]	Type	Width [min]	Area [mAU*s]	Height [mAU]	Area %
1	17.138	BB	0.6398	2.08640e4	479.35590	90.0680
2	20.187	BB	0.7513	2300.71606	44.05249	9.9320

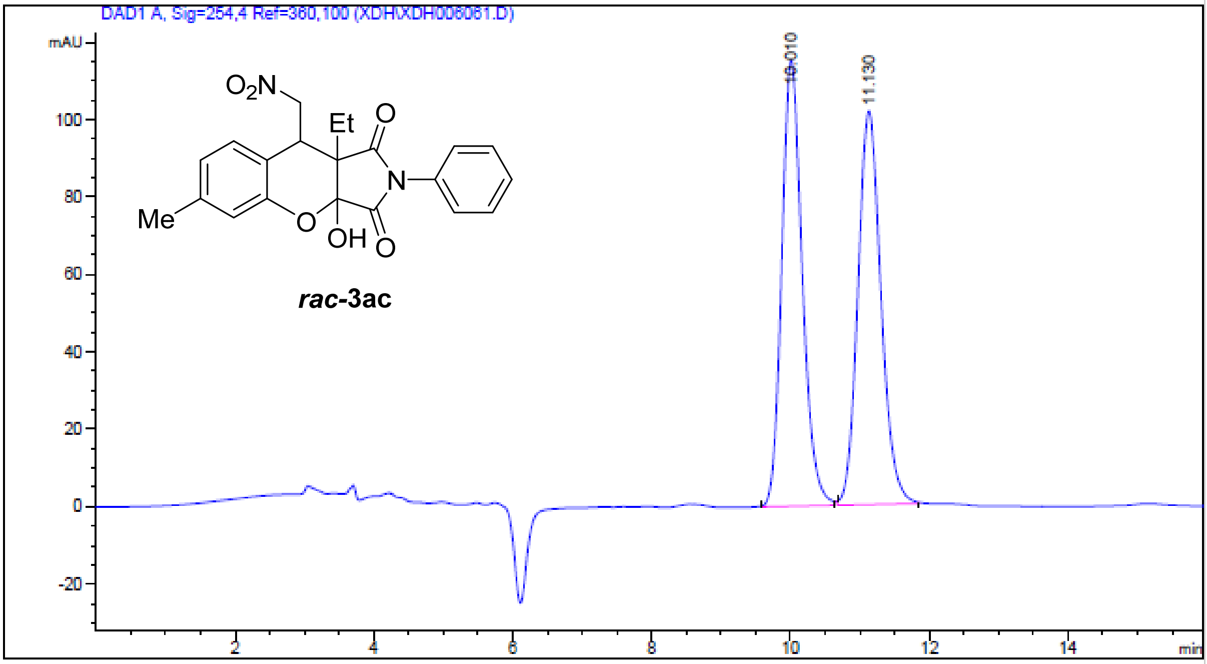

Peak #	RetTime [min]	Type	Width [min]	Area [mAU*s]	Height [mAU]	Area %
1	10.010	BB	0.3140	2370.31323	115.20665	50.1794
2	11.130	BB	0.3560	2353.36206	101.68330	49.8206

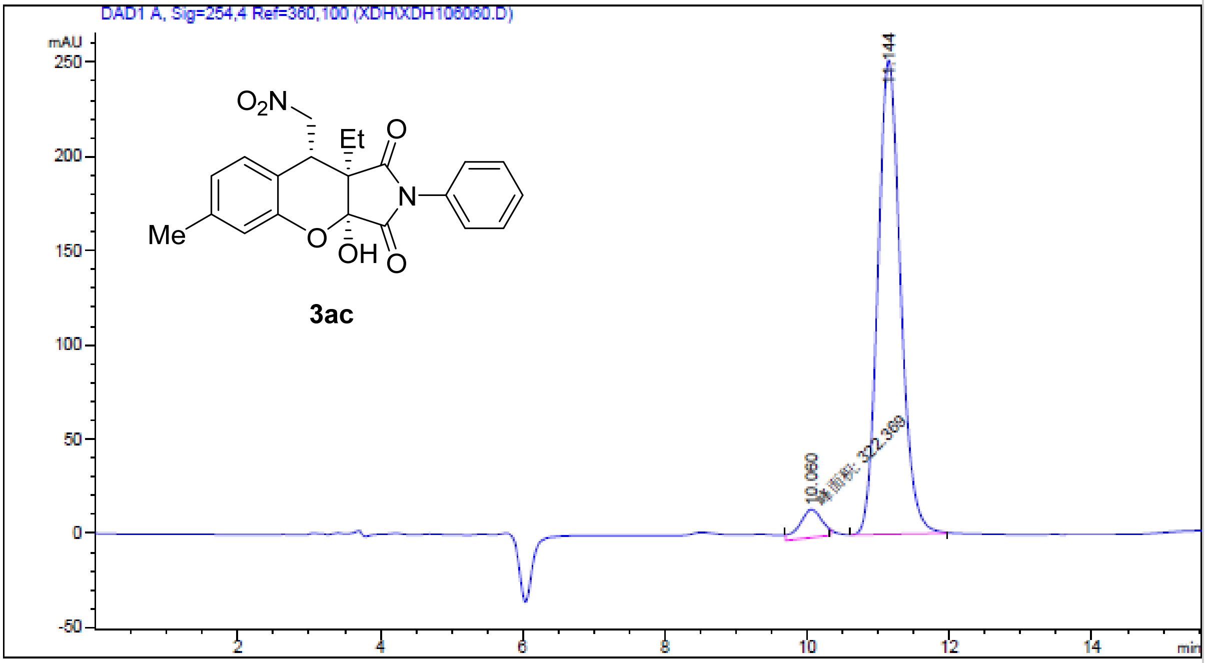

Peak #	RetTime [min]	Type	Width [min]	Area [mAU*s]	Height [mAU]	Area %
1	10.060	MM	0.3597	322.36893	14.93748	5.4618
2	11.144	VB	0.3407	5579.86670	251.59558	94.5382

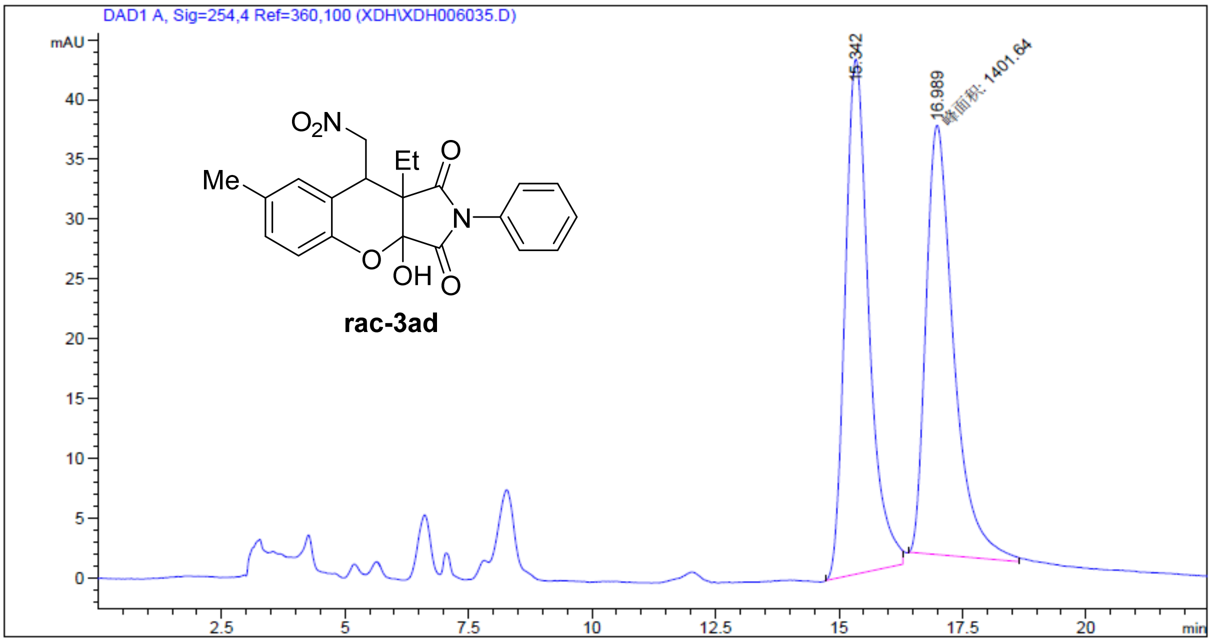

Peak #	RetTime [min]	Type	Width [min]	Area [mAU*s]	Height [mAU]	Area %
1	15.342	BB	0.5036	1437.82056	43.06126	50.6371
2	16.989	MM	0.6507	1401.63965	35.89898	49.3629

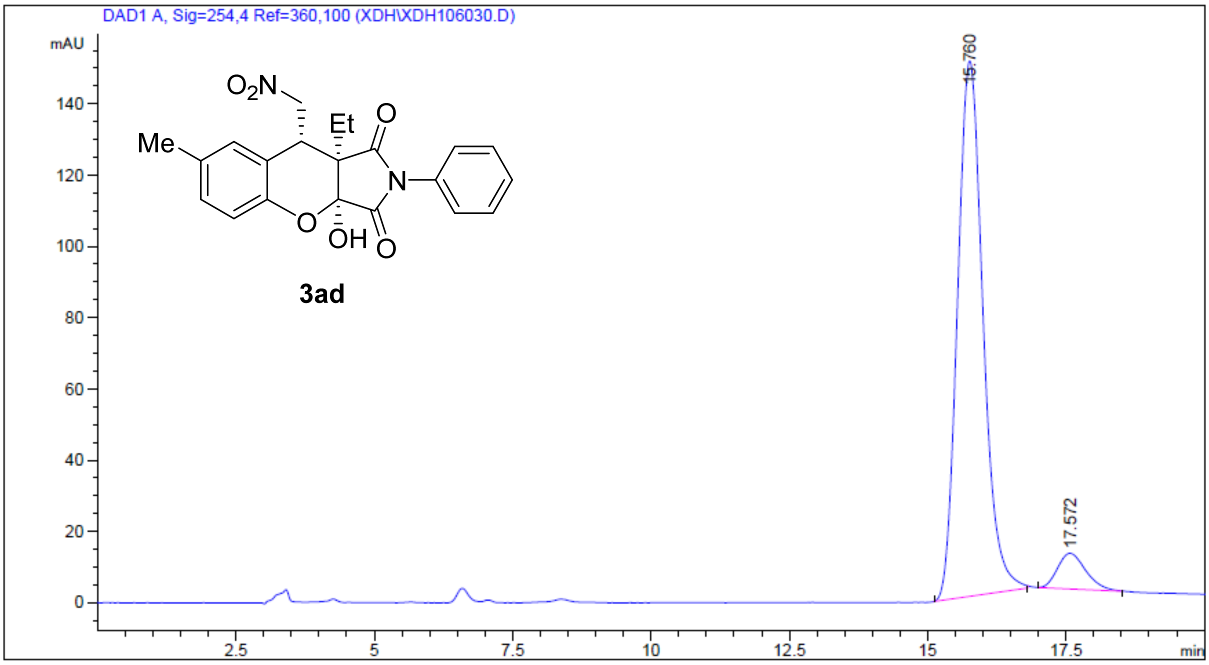

Peak #	RetTime [min]	Type	Width [min]	Area [mAU*s]	Height [mAU]	Area %
1	15.760	BB	0.4953	4806.25342	150.22047	93.0919
2	17.572	BB	0.5331	356.65659	10.02476	6.9081

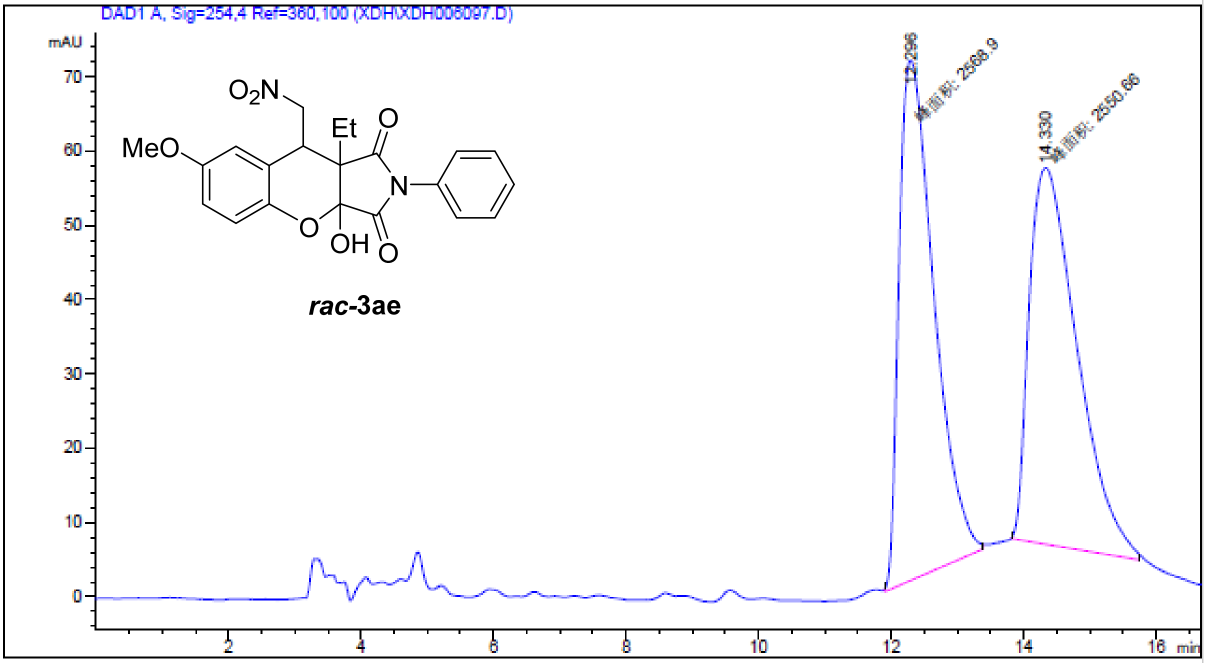

Peak #	RetTime [min]	Type	Width [min]	Area [mAU*s]	Height [mAU]	Area %
1	12.296	MM	0.6110	2568.89526	70.07661	50.1781
2	14.330	MM	0.8375	2550.66016	50.76049	49.8219

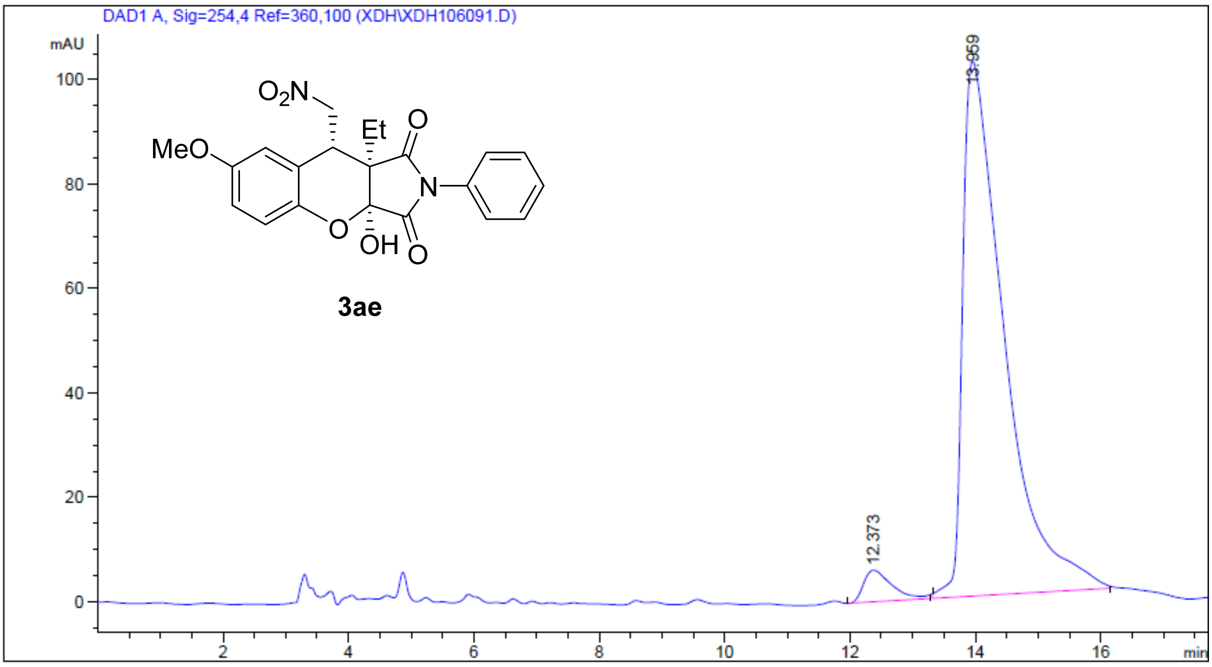

Peak #	RetTime [min]	Type	Width [min]	Area [mAU*s]	Height [mAU]	Area %
1	12.373	BB	0.4383	191.32764	6.06104	3.8998
2	13.959	BB	0.6491	4714.82080	102.42185	96.1002

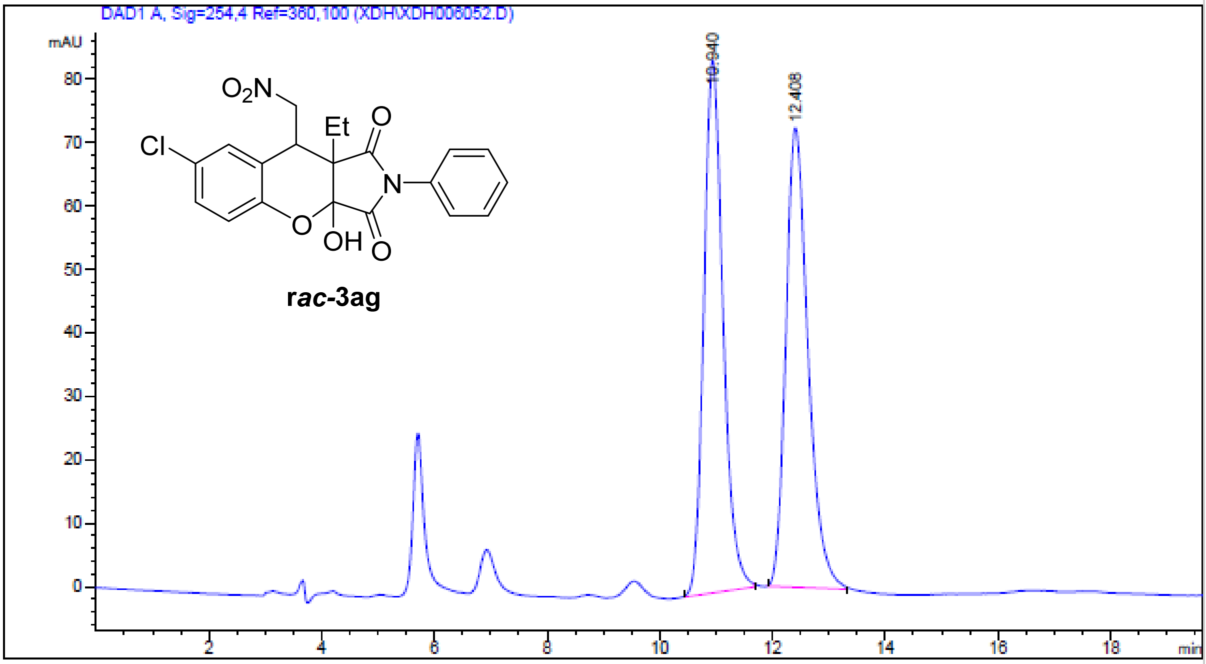

Peak #	RetTime [min]	Type	Width [min]	Area [mAU*s]	Height [mAU]	Area %
1	10.940	BB	0.3625	1990.13721	83.96015	50.0642
2	12.408	BB	0.4179	1985.03540	72.40098	49.9358

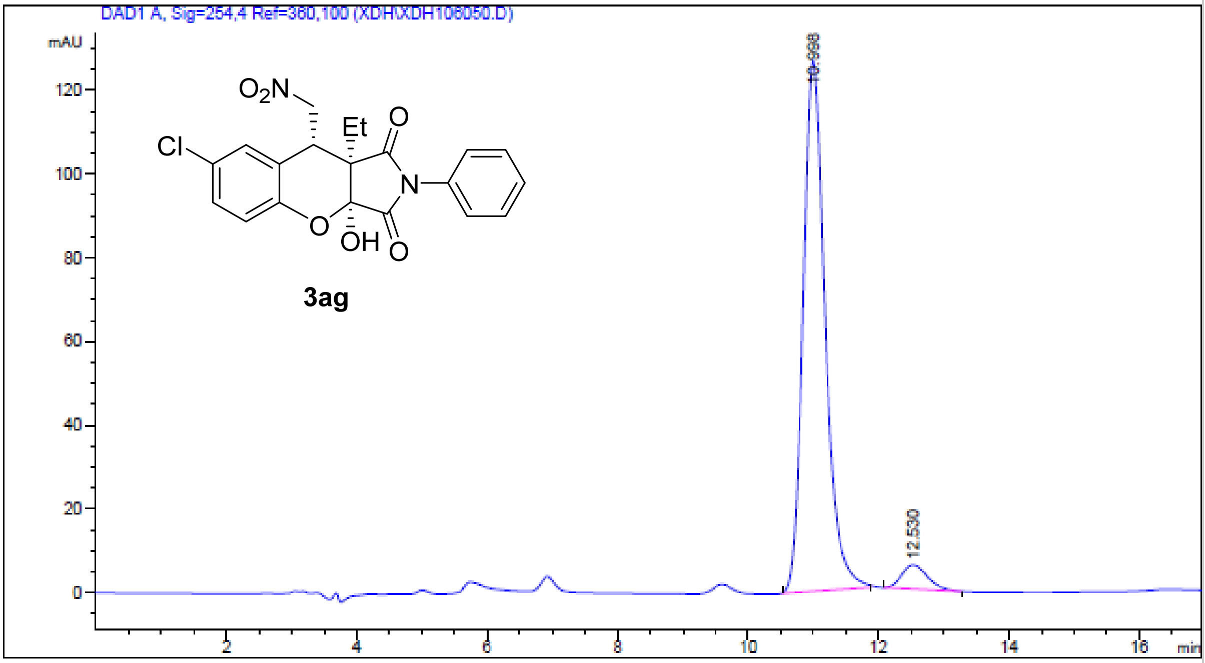

Peak #	RetTime [min]	Type	Width [min]	Area [mAU*s]	Height [mAU]	Area %
1	10.998	BB	0.3567	2967.54053	126.95973	94.9329
2	12.530	BB	0.3929	158.39273	5.75795	5.0671
